# The origin of Rhinocerotoidea and phylogeny of Ceratomorpha (Mammalia, Perissodactyla)

**DOI:** 10.1038/s42003-020-01205-8

**Published:** 2020-09-14

**Authors:** Bin Bai, Jin Meng, Chi Zhang, Yan-Xin Gong, Yuan-Qing Wang

**Affiliations:** 1 Key Laboratory of Vertebrate Evolution and Human Origins of Chinese Academy of Sciences, Institute of Vertebrate Paleontology and Paleoanthropology, Chinese Academy of Sciences, Beijing, 100044 China; 2 CAS Center for Excellence in Life and Paleoenvironment, Beijing, 100044 China; 3 Division of Paleontology, American Museum of Natural History, New York, NY 10024 USA; 4 Earth and Environmental Sciences, Graduate Center, City University of New York, New York, NY 10016 USA; 5 College of Earth and Planetary Sciences, University of Chinese Academy of Sciences, Beijing, 100049 China

**Keywords:** Palaeontology, Phylogenetics, Taxonomy

## Abstract

Rhinoceroses have been considered to have originated from tapiroids in the middle Eocene; however, the transition remains controversial, and the first unequivocal rhinocerotoids appeared about 4 Ma later than the earliest tapiroids of the Early Eocene. Here we describe 5 genera and 6 new species of rhinoceroses recently discovered from the early Eocene to the early middle Eocene deposits of the Erlian Basin of Inner Mongolia, China. These new materials represent the earliest members of rhinocerotoids, forstercooperiids, and/or hyrachyids, and bridge the evolutionary gap between the early Eocene ceratomorphs and middle Eocene rhinocerotoids. The phylogenetic analyses using parsimony and Bayesian inference methods support their affinities with rhinocerotoids, and also illuminate the phylogenetic relationships and biogeography of Ceratomorpha, although some discrepancies are present between the two criteria. The nearly contemporary occurrence of various rhinocerotoids indicates that the divergence of different rhinocerotoid groups occurred no later than the late early Eocene, which is soon after the split between the rhinocerotoids and the tapiroids in the early early Eocene. However, the Bayesian tip-dating estimate suggests that the divergence of different ceratomorph groups occurred in the middle Paleocene.

## Abstract (web summary)

Though modern species are on the brink of extinction, rhinos and tapirs have a diverse fossil record. Six new species of fossil rhinos reported by Bin Bai et al. show that rhinos evolved even earlier than previously thought, perhaps linked to the close, humid environments of the Early Eocene Climatic Optimum.

## Introduction

Both morphological and molecular studies support the idea that Rhinocerotoidea and Tapiroidea form a monophyletic group Ceratomorpha^1–3^. The ceratomorphs have abundant, diverse fossil records in the Cenozoic; however, extant ceratomorphs are reduced to five genera and on the brink of extinction. Furthermore, despite a long research history and numerous fossils, the phylogeny and evolutionary history of Ceratomorpha still remain contentious. Previous phylogenetic analyses have either focused on tapiroids^4,5^ or rhinocerotoids^6^ without the combination of both groups. Analyses that have contained both tapiroids and rhinocerotoids are still limited in taxa and character selection^1,7^, so that relationships within Ceratomorpha were not well resolved and many controversies still remained^1,4,5,8–11^.

**Fig. 1:**
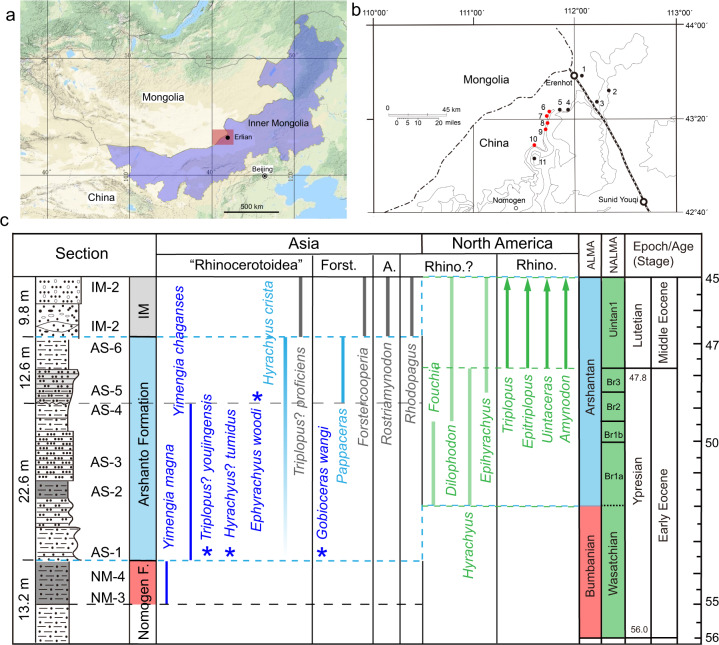
Fossil localities and distributions of early rhinocerotoids. **a** The location of the Erlian Basin of Inner Mongolia, China; **b** Paleogene fossil localities in the Erlian Basin. 1, Houldjin; 2, Arshanto; 3, Irdin Manha; 4, Daoteyin Obo; 5, Duheminboerhe; 6, Nuhetingboerhe; 7, Wulanboerhe; 8, Huheboerhe; 9, Chaganboerhe; 10, Bayan Ulan; 11, Nom Khong. The red dots refer to the localities where new materials were found. **c** Distributions of early controversial and unequivocal rhinocerotoids and new material from the early Eocene and early–middle Eocene in the Erlian Basin. The dark blue bars and stars show the distributions of new rhinocerotoids from the Erlian Basin. The light blue, gray, and green bars represent previously described early rhinocerotoids (or controversial rhinocerotoids) known from Asia and North America. Abbreviations: A. Amynodontidae, Br. Bridgerian, Forst. Forstercooperiidae, Rhino. Rhinocerotoidea.

Rhinocerotoidea conventionally comprises Hyracodontidae, Amynodontidae, and Rhinocerotidae^10,12^, with paraceratheres (giant rhinos) recently treated as a separated family derived from Hyracodontidae^13^. Rhinocerotoids probably originated from ‘*Hyrachyus*’ (or Hyrachyidae), which spread from Eurasia to North America in the middle Eocene, and has usually been considered to be a transitional form from the tapiroids to rhinocerotoids^6,14–16^. However, the postcranial skeleton of *Hyrachyus* suggests that ‘*Hyrachyus*’ arose from tapiroids more primitive than *Heptodon*, and could not be an ancestor of *Triplopus*, which bears a specialized skeleton for fast running^14^. So *Hyrachyus* could not be ancestry to all rhinocerotoid groups. The earliest hyracodontids and amynodontids are represented by *Triplopus* and *Amynodon*/*Rostriamynodon*, respectively, from the early Uintan North American Land Mammal Age (NALMA)^17^ and/or Irdin Manhan Asian Land Mammal Age (ALMA)^18^ (Fig. 1). Rhinocerotidae also appeared in the early Uintan as represented by its sister group *Uintaceras*^19^. Recently, Wang et al.^11^ reported the earliest unequivocal rhinocerotoid *Pappaceras meiomenus* from the early–middle Eocene of Arshantan ALMA (Fig. 1c), which is slightly earlier than any other known rhinocerotoids and considered to be ancestral to later giant rhinos. But *Pappaceras* is already more derived than ‘*Hyrachyus*’, and possesses a combination of both paraceratheriid and amynodontid characters, suggesting a close relationship between these two families^11,20^. Except for *Pappaceras*, unequivocal rhinocerotoids have not been reported from the early Eocene or early–middle Eocene in either North America or Asia, although some relatively small ceratomorphs have been argued to be rhinocerotoids, such as *Fouchia*, *Dilophodon*, *Rhodopagus*, and *Yimengia*^21–23^ (Fig. 1c).

Here on the basis of new, diverse rhinocerotoid materials from the early Eocene to the early–middle Eocene in the Erlian Basin of Inner Mongolia, China (Fig. 1a, b), we describe five genera (including a new genus) and six new species that represent earliest members of rhinocerotoids, forstercooperiids, and/or hyrachyids (Fig. 1c). We further resurrect the genus *Ephyrachyus*, and erect a new species of *Ephyrachyus*. These new materials are unearthed from the upper part of the Nomogen Formation and the Arshanto Formation, which are considered to be the early Eocene Bumbanian and the early–middle Eocene Arshantan ALMA^24^, respectively. The Bumbanian is normally comparable with Wasatchian NALMA, and the Arshantan is comparable with Bridgerian plus the early Uintan NALMA based on the mammal fauna correlation and the recent paleomagnetic analyses^25,26^. These new diverse rhinocerotoids bridge the evolutionary gap between the early Eocene ceratomorphs and middle Eocene Uintan/Irdin Manhan rhinocerotoids, and suggest that divergence of different rhinocerotoid groups occurred no later than the late early Eocene in a relatively close, humid environment.

## Results

### Systematic paleontology

Perissodactyla Owen, 1848

Rhinocerotoidea Gray, 1825

Family incertae sedis

*Yimengia* Wang, 1988^27^

**Type.** *Yimengia yani* Wang, 1988^27^

**Included species.** *Y. laiwuensis*^28^, *Y. zdanskyi*^23^, *Y. magna* sp. nov., *Y. chaganense* sp. nov.

**Locality and horizon.** Early–middle Eocene; Guanzhuang Formation, Laiwu and Xintai county, Shandong Province; Nomogen and Arshanto formation, Erlian Basin, Inner Mongolia.

**Diagnosis.** Differs from *Rhodopagus* in having P4 metaconule contacting the base of the protocone, M1–2 metacone less lingually appressed and more elongated without bulges at the base of the buccal side, M3 with a more distinct metacone, and centrocrista not confluent with the metaloph, p3–4 paraconid and hypoconid relatively lower, cristid obliqua more lingually slanted, p3 metaconid separated from the protoconid, p4 entoconid less distinct, and the lower molars with relatively longer trigonid, more transversely extended protoloph, and more lingually extended cristid obliqua with a relatively higher contact with the protolophid. Differs from *Minchenoletes* in having a more lingually placed metacone on M1–3, metaloph of M1–3 joining the ectoloph less forward, M3 metacone more reduced, and less distinct hypoconulids on lower molars. Differs from *Triplopus* (as represented by *T. cubitalus*) in having the metaconule not forming a loop with the protoloph on P3–4, a shorter metacone on P3–4, parastyles of upper molars less reduced, M3 metacone more distinct and less lingually appressed, cristid obliqua of p3–4 more lingually slanted, and protolophid more transversely extended on the lower molars.

*Yimengia* *magna* sp. nov.

**Fig. 2:**
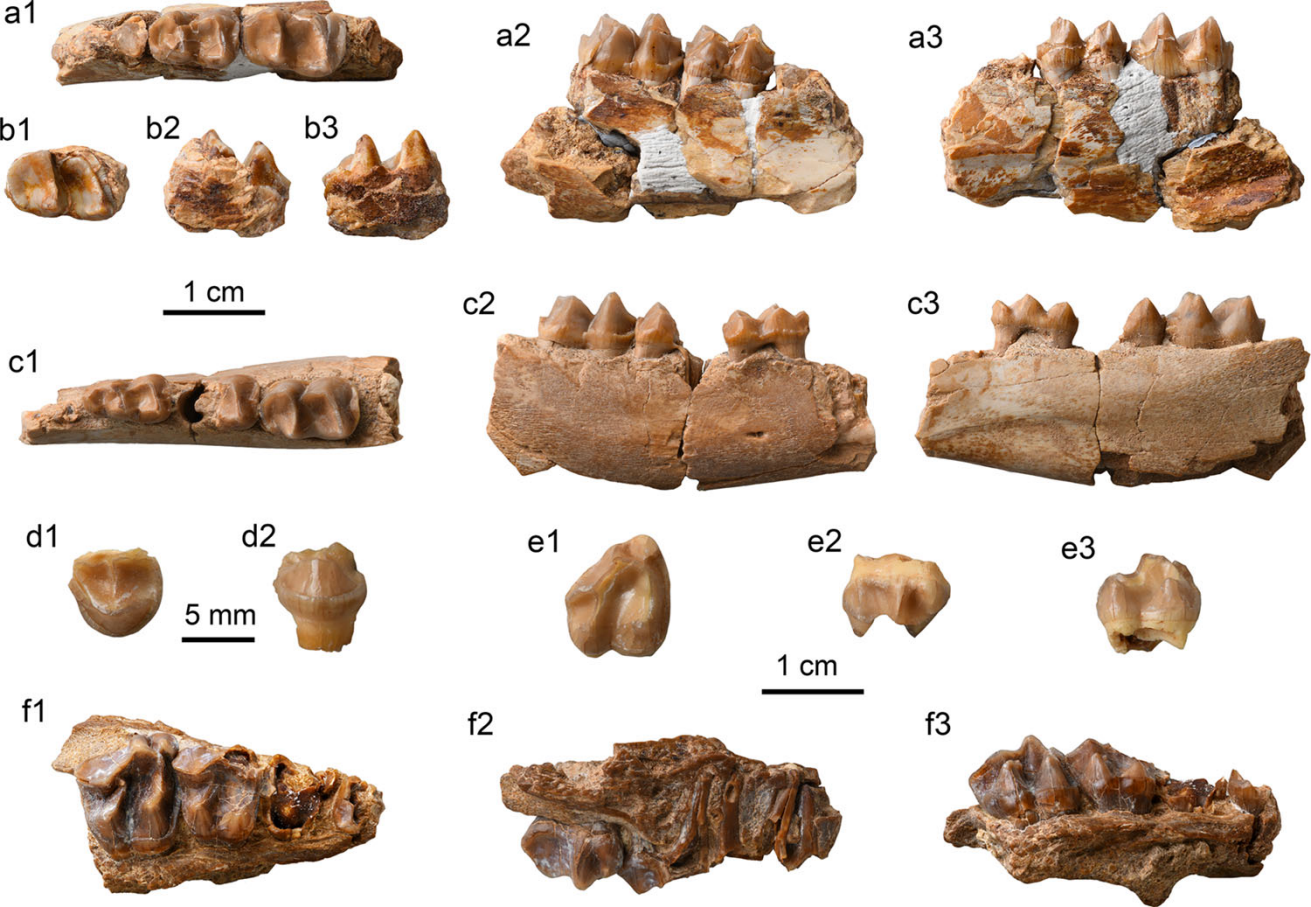
Specimens of *Yimengia magna* sp. nov. from the upper part of the Nomogen Formation of the Erlian Basin. **a**, **b** Right mandible with dp4-m1 (**a**), m3 (**b**) (IVPP V 26234, holotype) in occlusal (a1, b1), buccal (a2, b2), and lingual (a3, c3) views; **c** right mandible with dp3–m1 (IVPP V 26235) in occlusal (c1), buccal (c2), and lingual (c3) views; **d** partial left P4 (IVPP V 26238.1) in occlusal (d1) and lingual (d2) views; **e** right M3 (IVPP V 26238.2) in occlusal (e1), buccal (e2), and lingual (e3) views; **f** right maxilla with M1–2 (IVPP V 26241) in occlusal (f1), buccal (f2), and lingual (f3) views.

**Holotype.** IVPP V 26234, a right mandible with dp4, m1, and m3 (Fig. 2a, b).

**Referred specimens.** IVPP V 26235, an associated right mandible with dp3, talonid of dp4, m1 (Fig. 2c), and a left mandible with m1; V 26236, a right m3; V 26237, an associated left mandible with m1 and a right mandible with broken talonid of m1; V 26238, a left P4 with the ectoloph broken off (Fig. 2d), a fragmentary upper molar, and a right M3 (Fig. 2e); V 26239, a left m1/2; V 26240, a left mandible with fragmentary m1; V 26241, a right maxilla with M1–2 (Fig. 2f).

**Etymology.** The Latin ‘*magnus*’ means large, referring its relatively large size within the genus.

**Localities and horizons.** Early–early Eocene, upper part of the Nomogen Formation. V 26234: 0.2–0.3 m above the *Gomphos* bed, Nomogen Formation, Nuhetingboerhe. V 26235–V 26236: *Gomphos* bed, Nomogen Formation, Nuhetingboerhe; V 26237–V 26238: 0.5 m above the *Gomphos* bed, Nomogen Formation, Nuhetingboerhe; V 26239: *Gomphus* bed, Nomogen Formation, Wulanboerhe; V 26240: upper part of the Nomogen Formation, Huheboerhe; V 26241, *Gomphos* bed?, Nomogen Formation, Bayan Ulan.

### Diagnosis (Table 1 and Supplementary Note 1)

Differs from other species of *Yimengia* by a larger size, a slightly less lingually placed metacone with a weak rib or convexity on the buccal side on the upper molars, and m3 hypoconulid more developed; further differs from *Y. chaganense* by a larger, more buccally placed parastyle on the upper molars, and more distinct ribs on the anterior sides of the metaconid and protoconid on the lower molars; further differs from *Y. yani*, *Y. laiwuensis*, and *Y. zdanskyi* by a stronger cristid obliqua joining the protolophid in a high position on m1–2.

*Yimengia* *chaganense* sp. nov.

**Table 1:** Measurements of *Yimengia magna* and *Y. chaganense* (in mm).

	P4 L/W	M1 L/W	M2 L/W	M3 L/W	m1 L	m1 AW/PW
*Yimengia magna*						
V 26241		*9.4/9.5*	10.8/11.6			
V 26238				10.9/12.1		
V 26245.3					9.4	5.1/5.3
V 26247.1					8.3	5.6/5.7
*Yimengia chaganense*						
V 26242.1	6.3/8.4	8.9/9.9	*9.7/9.5*	*9.5/9.8*		
V 26242.2		9.0/9.6				
V 26234					10.7	6.0/*6.8*
V 26235					9.9	6.0/6.3
V 26239					10.1	6.4/6.7

Italic number: approximate measurements.

**Fig. 3:**
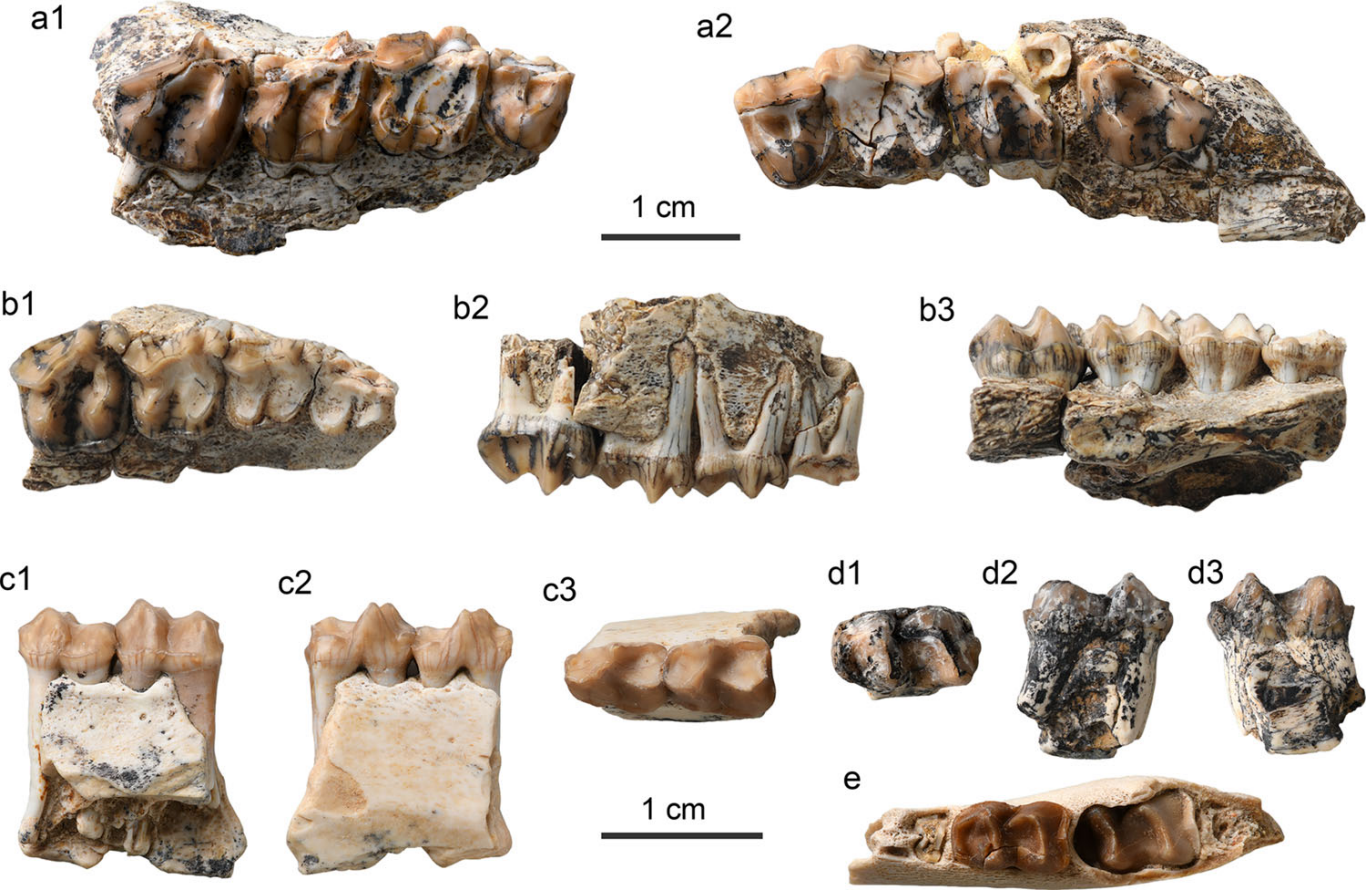
Specimens of *Yimengia chaganense* sp. nov. from the lower and middle parts of the Arshanto Formation of the Erlian Basin. **a** Right (a1) and left (a2) maxillae with P4–M3 (IVPP V 26242.1, holotype) in occlusal view; **b** right maxilla with DP2-DP4 and M1 (IVPP V 26242.2) in occlusal (b1), buccal (b2), and lingual (b3) views; **c** left mandible with p3–4 (IVPP V 26243) in buccal (c1), lingual (c2), and occlusal (c3) views; **d** right m1/2 (IVPP V 26245.3) in occlusal (d1), buccal (d2), and lingual (d3) views; **e** right mandible with dp4 and m1 in the alveolus (IVPP V 26247.1) in occlusal view.

**Holotype.** IVPP V 26242.1, associated left and right maxillae with P4–M3 with ectolophs partially broken off (Fig. 3a).

**Referred specimens** IVPP V 26242.2, associated juvenile left and right maxillae with DP2–4 and M1 (Fig. 3b); V 26243, a left mandible with p3–4 (Fig. 3c); V 26244, a left M1/2; V 26245.1, a left M1/2; V 26245.2, .3, a left M1/2 and m1 (Fig. 3d); V 26246.1-3, a left M1, a right M2, and a fragmentary M3; V 26247.1-3, an isolated left dp4, a right mandible with dp4 and m1 in the alveolus (Fig. 3e), and a right mandible with dp4–m1.

**Etymology.** The specific name ‘*chaganense*’ refers to Chaganboerhe, where the holotype was found.

**Localities and horizons.** Late early Eocene, low and middle parts of the Arshanto Formation. V 26242, lower horizon of the middle part of the Arshanto Formation, Chaganboerhe; V 26243, upper horizon of the middle part of the Arshanto Formation, Chaganboerhe; V 26244, lower part of the Arshanto F. (As2), Chaganboerhe; V 26245, upper horizon of the middle part of the Arshanto Formation, Huheboerhe; V 26246, lower part of the Arshanto F. (As2), Huheboerhe; V 26247, basal part of the Arshanto F., Nuhetingboerhe.

### Diagnosis (Table 1 and Supplementary Note 1)

Differs from other species of *Yimengia* by smaller parastyles on the upper molars; differs from *Y. magna* by a more lingually placed parastyle and a flat, more lingually placed metacone on M1–3, and more reduced m3 hypoconulid; differs from *Y. yani*, *Y. laiwuensis*, and *Y. zdanskyi* by a stronger cristid obliqua joining the protolophid in a high position on m1–2; further differs from *Y. yani* by a flat metacone on P4; further differs from *Y. laiwuensis* by a more distinct metaconid on p3.

### Comparisons

**Fig. 4:**
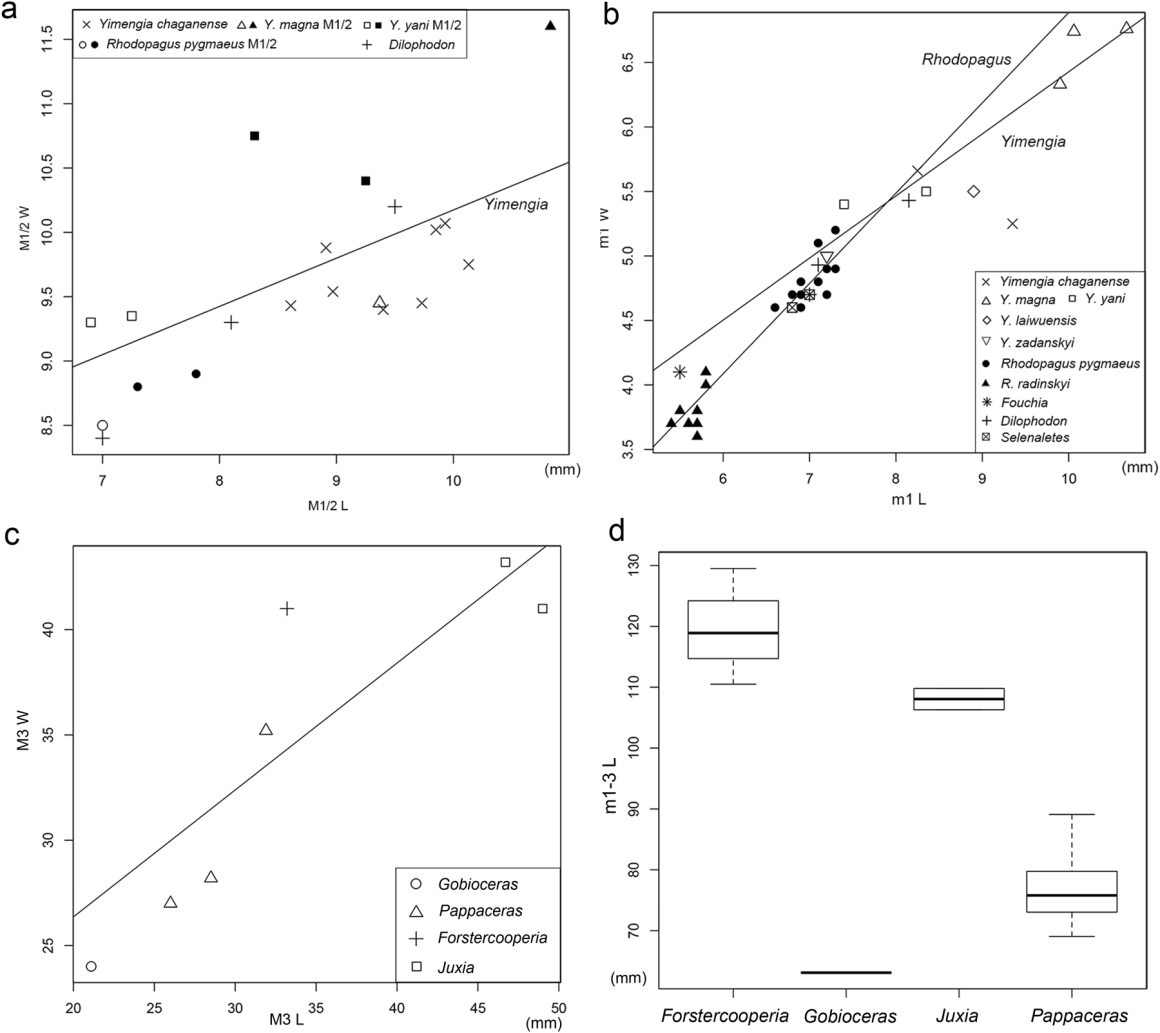
Scatter plots and box plot of dental proportions and length. *Yimengia*, *Rhodopagus*, and other early ceratomorphs (**a**, **b**), *Gobioceras*, *Pappaceras*, *Forstercooperia*, and *Juxia* (**c**, **d**). **a** Scatter plots of M1/2 proportion with the regression line for width as a function of length in *Yimengia*. **b** Scatter plots of m1 proportions with the regression line for width as a function of length in *Yimengia* and *Rhodopagus*. **c** Scatter plot of M3 proportion with the regression line for width as a function of length. **d** Box plot of m1–3 length. Box represents 25% and 75% quartiles, and the dotted line has a length of 1.5 times the interquartile range. *n* = 3, 1, 2, and 8 biologically independent samples, respectively.

These two new species are characterized by small to medium size among early ceratomorphs, a reduced parastyle and pinched paracone on M1–3, a flat metacone with relatively long postmetacrista on M1–2, M3 metacone short and strongly lingually depressed, cristid obliqua of p3–m3 strong and joining the protolophid in a relatively high position, and absence of m3 hypoconulid. Almost all characters of the new materials are similar to those of *Yimengia*, which was previously known by three species from the Guanzhang Formation, Shandong Province^27^. However, the type of *Yimengia*, *Y. yani*, has stronger parastyles on upper molars, a relatively wider M1 (Fig. 4a), a more distinct metacone rib on P4, and lower cristid obliqua on m1–3 than in the new taxa.

*Y. laiwuensis*^28^ and *Y. zdanskyi*^23,29^, which were originally assigned to *Rhodopagus*, were known only from the low jaws. *Y. laiwuensis* is different from Erlian specimens in having a less basined trigonid on p3 with a more reduced metaconid, and a relatively lower cristid obliqua on p3–m3. Wang^27^ further interpreted a left mandible with two molars of *Y. zdanskyi* (PMUM 3004) as m1–2 rather than m2–3^23^. *Y. zdanskyi* is mainly different from Erlian species in having a smaller size (Fig. 4b), and m1–2 with a more triangular trigonid and more reduced cristid obliqua.

*Yimengia* is considered to be closely related to *Rhodopagus*^27^, which is known from later Irdin Manha and Shara Murun faunas, as represented *Rhodopagus* *pygmaeus* and *R. minimus*, respectively^30^. Although Lucas and Schoch^23^ regarded *R. pygmaeus* as a synonym of *R. minimus*, we have treated them here as separate species pending a discovery of more complete material of *R. minimus*. ‘*Rhodopagus*’ *minutissimus* from the middle Eocene of Andarak in Kyrgyzstan^31^ was later considered to be *Pataecops minutissimus*^32^. *Rhodopagus* (as represented by its best-known species *R. pygmaeus*) is mainly different from *Y. magna* and *Y. chaganense* in having a straight ectoloph on P2–4 with a relatively higher parastyle that occludes with the corresponding high, nearly straight, buccally aligned paralophid and cristid obliqua on p2–3. Further, after careful observation of abundant, nearly unworn lower molars of *R. pygmaeus* recently unearthed from the ‘Basal White’ of Erden Obo^18^, we notice that the ‘long anterior paralophid’ is actually composed of an anterior paralophid on the buccal half and a cingulum on the lingual half that rises from the anterobuccal cingulum and is nearly confluent with the real anterior paralophid. This configuration is usually obliterated and indistinct after wear.

Gabunia and Kukhaleishvili^33^ described *R. radinskyi* from the late early Eocene^32^ or early–middle Eocene Chakpaktas Svita^34^ in the Zaysan Basin, Kazakhstan. *R. radinskyi* resembles *Yimengia* in having a flat and relatively long metacone on M1–3 with postmetacrista slightly buccally deflected, and distinct cingula along the anterior border and lingual side of the M1–3 protocone. However, *R. radinskyi* is much smaller than *Yimengia* (Fig. 4b), and shares with *R. pygmaeus* in having (1) a high, straight P3–4 ectoloph, (2) continuous high longitudinal buccal ridges composed of the paralophid and cristid obliqua on p3–4^35^, and (3) strong parastyle on M1–3.

*Veragromovia*, which was unearthed from the middle Eocene Zaysan Basin of Kazakhstan^36^, has also been considered to be a junior synonym of *Helaletes*^30^. But the genus was later resurrected and assigned to the Rhodopagidae^35^. M3 of *Veragromovia* is different from that of *Yimengia* in having a larger parastyle, a more reduced and slightly buccally deflected metacone, and a complete lingual cingulum.

Lophialetids are common, endemic tapiroids distributed in the early and middle Eocene of Asia^30^. *Minchenoletes* and *Schlosseria* have been reported from the Nomogen and Arshanto formations, respectively^37,38^, and the size of *Y. magna* is intermediate between them. The early Eocene *Y. magna* strikingly show some similarities with contemporary *Minchenoletes* and later *Schlosseria* in having a flat, long metacone on M1–2 and a strong cristid obliqua on m1–3. *Y. magna* is further similar to *Minchenoletes* in having a pinched paracone on M1–3, and relatively more anteriorly directed cristid obliqua on m1. However, both *Minchenoletes* and *Schlosseria* differ from *Yimengia* by having a more buccally placed metacone on M1–3, M1–3 metaloph joining the ectoloph relatively far forward, more elongated M3 metacone, and more distinct hypoconulids on lower molars.

The conventional lophialetid *Breviodon minutus* (=*B. acares*) from the Arshanto and Irdin Manha formations is similar to *Y. chaganense* in size, but its molar morphology is generally like those in *Schlosseria* and *Lophialetes*^30,31,39^ and in turn differs from *Yimengia*. *Breviodon* further differs from *Yimengia* in lacking p1–2, and thus having the premolar series relatively shorter than the molar series. Another lophialetid *Parabreviodon*, initially assigned to Cf. *B. acares* by Radinsky^30^ and later erected as a new genus by Reshetov^40^, is known by a partial cranium (AMNH FM 81751) from the Arshanto Formation^30^. The upper cheek teeth of *Parabreviodon* mainly differs from those of *Yimengia* in being relatively shorter and wider, and in having a more convex metacone on P4–M3, protoloph and metaloph on P4 forming a V-shaped loop, and more buccally placed metacone on M1–3 with larger parastyle and a longer M3 metacone.

Three small ceratomorphs from North America, *Dilophodon*^41,42^, *Selenaletes*^43^, and *Fouchia*^22^, were known from early and middle Eocene. *Yimengia* mainly differs from them in the following combined characters: less molarized premolars (compared to *Dilophodon*), a flatter and more lingually placed metacone on M1–3 with an elongated postmetacrista (compared to *Dilophodon* and *Fouchia*), and a stronger cristid obliqua on m1–3 with a high joint on the protolophid.

It is not unexpected to note that *Yimengia* shows some similarities with the hyracodontid *Triplopus cubitalus*^9^ in having a relatively small parastyle, a pinched paracone, a lingually situated and relatively long, flat metacone on M1–2, reduced M3 metacone, a strong cristid obliqua anteriorly directed on m1–3, and reduced m3 hypoconulid. However, *T. cubitalus* differs from *Yimengia* in having a loop formed by the protoloph and metaloph on P3–4, a smaller parastyle on M1–3, a smaller and more lingually appressed metacone on M3, vertical cristid obliqua on p3–4 with longer paralophid, and more oblique protolophid and relatively higher cristid obliqua on m1–3.

Family incertae sedis

*Triplopus*? *youjingensis* sp. nov.

**Fig. 5:**
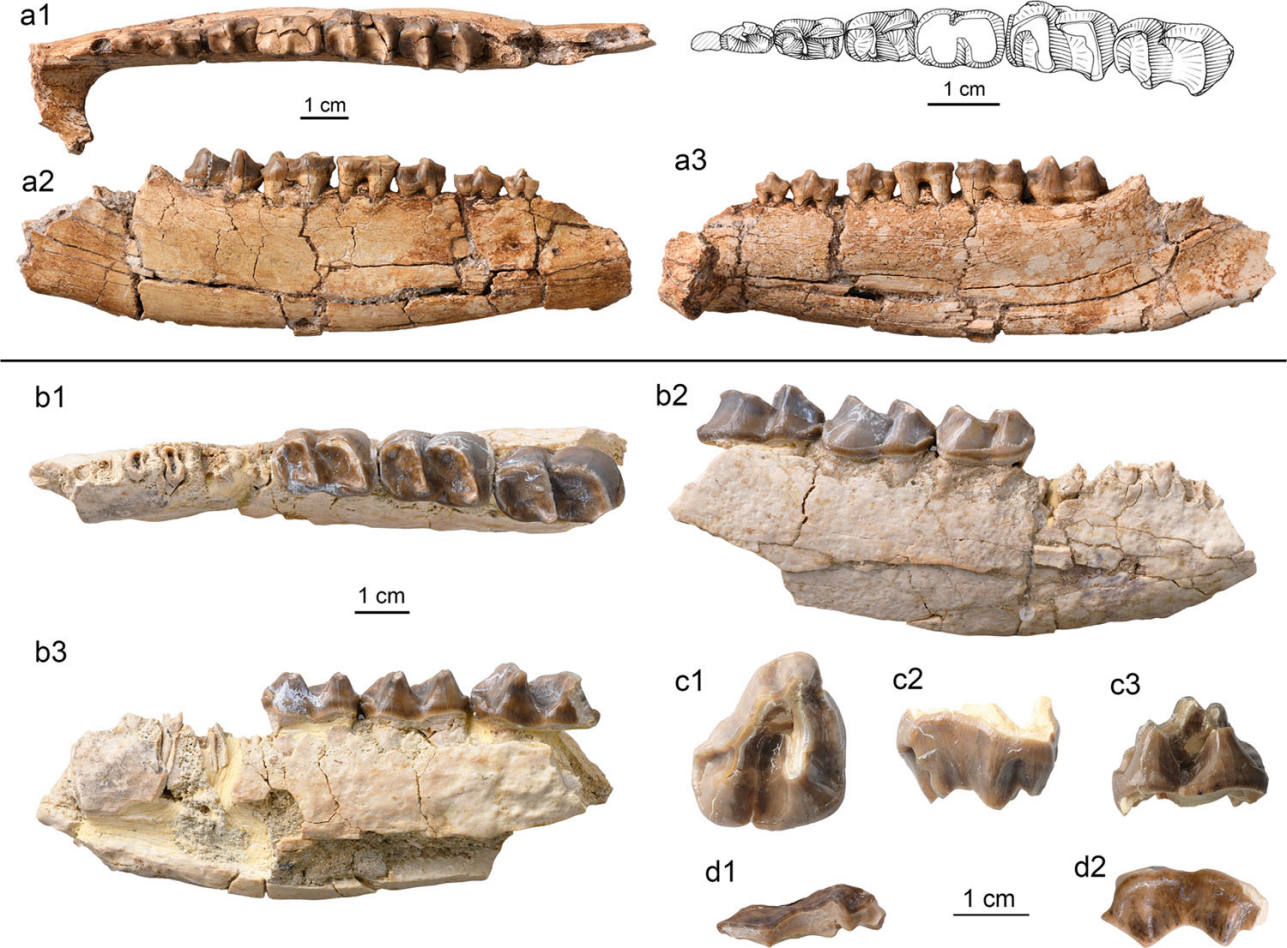
Specimens of *Triplopus*? *youjingensis* sp. nov. and *Gobioceras wangi* gen. et sp. nov. from the base of the Arshanto Formation in the Erlian Basin. **a** *T*.? *youjingensis*, right mandible with p2–m3 (IVPP V 26248, holotype) in occlusal (a1), buccal (a2), and lingual (a3) views; **b**–**d** *G. wangi*, **b** right mandible with m1–3 (IVPP V 26249, holotype) in occlusal (b1), buccal (b2), and lingual (b3) views; **c** right M3 (IVPP V 26250.1) in occlusal (c1), buccal (c2), and lingual (c3) views; **d** fragmentary M2 ectoloph (IVPP V 26250.2) in occlusal (d1) and buccal (d2) views.

**Holotype.** IVPP V 26248, a right mandible with p2–m3 (Fig. 5a).

**Etymology.** The specific name ‘youjing’ means ‘oil well’ in pinyin (phonetic transcription) of the Chinese language, referring to the oil company nearby the fossil locality.

**Locality and horizon** Late early Eocene, basal part of the Arshanto Formation, Nuhetingboerhe.

**Diagnosis (Table** **2****and Supplementary Note** 1**).** Medium-sized ‘hyracodontid’ with low crowned teeth; differs from other species of *Triplopus* by p3–4 with a rudimentary hypolophid, and the parallel protolophid and hypolophid nearly transversely extended on m1–3. Further differs from *T*.? *proficiens* by a more anteriorly directed cristid obliqua on m1–3. Further differs from North American *Triplopus* by a slightly more lingually directed paralophid on m1–2.

**Table 2:** Measurements of *Triplopus*? *youjingensis* (V 26248), *Gobioceras wangi* (V 26249), *Ephyrachyus woodi* (V 26252), and *Hyrachyus*? *tumidus* (V 26253.1) (in mm).

	p1 L/W	p2 L/W	p3 L	p3 AW/PW	p4 L	p4 AW/PW	m1 L	m1 AW/PW	m2 L	m2 AW/PW	m3 L	m3 AW/PW	m1–3 L	p1–4 L	p1–4/ m1–3
*T*.? *youjingensis*	*5.0/3.7*	7.8/4.3	*10.7*	5.7/6.2	10.5	6.9/*7.6*	13.5	8.4/8.43	15.9	*9.0/8.7*	17.5	9.3/9.2	46.4	*34.3*	0.74
*G. wangi*							18.4	11.7/12.4	20.9	12.8/13.6	23.6	13.3/13.4	63.1		
	P1 L/W	P2 L/W	P3L	P3W	P4L	P4W	M1L	M1W	M2L	M2W	M3L	M3 W	M1–3 L	P1–4 L	P1–4/M1–3
*E. woodi*	*5.7/3.8*	9.3/8.7	10.3	12.0	11.8	*12.9*	*16.1*	*15.5*	17.5	16.9	14.5	16.1	*43.2*	*38.1*	0.88
*H*.? *tumidus*			*13.4*	*16.5*	*14.8*				21.4						

Italic number: approximate measurements.

**Comparisons.** The lower jaw shows some characters associated with rhinocerotoids: relatively high paraconids on the lower check teeth, a strong cristid obliqua of m1–3 joining the protolophid in a relatively high position, and the lack of an m3 hypoconulid lobe. The strong cristid obliqua on m1–3 in the new specimen differs from the reduced, low cristid obliqua of the lower molars in *Hyrachyus*. Further, the relatively small size of the new material, the presence of p1, and the anterolingually extended paralophid on m1–2 are suggestive of *Triplopus* affinity.

In the Erlian Basin, *Triplopus*? *proficiens* has been reported from the overlying Irdin Manhan and Ulan Shireh formations^9,44^. *T*.? *proficiens* is more advanced than the new material in having more molarized premolars, more oblique protolophid and hypolophid on m1–3, and the cristid obliqua of p3–m3 more lingually directed. The convex posterior border of m3 in *T*.? *youjingensis* is more similar to that of *T*.? *proficiens* from the Irdin Manha Formation than to those from the Ulan Shireh Formation which have a straighter posterior border of m3. *Triplopus*? *progressus* known from the later Shara Murun Formation can be distinguished by its smaller size (M1–3 length = 35 mm)^9^.

*Triplopus*? *mergenensis* from the middle Eocene Mergen locality of Mongolia^45^ is distinguished from *T*.? *youjingensis* by larger size (m1–3 length = 70 mm), a more prominent hypolophid on p3–4, and a more transversely extended protolophid on m2–3. *T. ckhikvadzei* from the Zaysan Basin of Kazakhstan^46^ is mainly different from *T*.? *youjingensis* in having a larger size (m1–3 length = 57.5 mm), and in lacking p1. The p2–4 of *T. ckhikvadzei* is very similar to that of *T*.? *proficiens*^13^, and in turn different from that of *T*.? *youjingensis*.

Compared with North American *Triplopus*, *T*.? *youjingensis* is considerably larger than *T. cubitalis*, slightly larger than *T. obliquidens*, and smaller than *T. rhinocerinus*^9,47^. In morphology, *T*.? *youjingensis* is mainly different from North American *Triplopus* by the relatively lower crown height, more transversely extended protolophid and hypolophid on m1–3, and somewhat more lingually directed paralophid on m1–2. On the other hand, *T*.? *youjingensis* is similar to North American *Triplopus* in having the cristid obliqua of m1–3 joining the protolophid in a position slightly lingual to protoconid.

Compared with contemporary *Schlosseria* from the Arshanto Formation^30^, *T*.? *youjingensis* can be distinguished by much larger size, slightly more oblique protolophid, more lingually extended paralophid on m1–2, relatively more anteriorly extended cristid obliqua on m1–3, a reduced hypoconulid on m1–2, and the lack of m3 hypoconulid lobe. Further, the metaconid of p3–m3 in *Schlosseria* is more or less cuspate with a convex anterior surface, whereas that in *T*.? *youjingensis* is merged with the protolophid with a nearly flat anterior surface.

To sum up, this mandible mostly resembles *Triplopus* in morphology, and its early Eocene age is earlier than other known species of *Triplopus*. But the genus *Triplopus* is also a complex issue to deal with. It contains four species from North America after Radinsky^9^ synonymized *Prothyracodon*, *Eotrigonias*, and *Ephyrachyus* with *Triplopus*. However, it is uncertain whether *Triplopus* is a monophyletic taxon and that all synonymies are reasonable (for example, see *Ephyrachyus* below). Thus, we assigned the new species to *Triplopus* with a query, pending a more comprehensive review of this genus.

Forstercooperiidae Kretzoi, 1940

*Gobioceras wangi* gen. et sp. nov.

**Holotype.** IVPP V 26249, a right mandible with m1–m3 (Fig. 5b).

**Referred specimens.** IVPP V 26250.1, .2, a right M3 (Fig. 5c), an ectoloph of right M2 (Fig. 5d); V 26251, associated left and right mandibles with talonid of dp3, dp4–m2, and m3 in the alveolus.

**Etymology.** The root ‘*Gobi*’ refers to the Gobi area, where the holotype was found; the suffix ‘ceras’ means horn, a common suffix used in rhinocerotoid names. The specific name honors Prof. Jin-Wen Wang, for his contributions to the study of Paleogene perissodactyls from China.

**Locality and horizon.** Late early Eocene, basal part of the Arshanto Formation, Nuhetingboerhe.

**Diagnosis (Table** **2****and Supplementary Note** 1**).** A relatively small forstercooperiid; Differs from *Pappaceras* by relatively larger and more cuspate M3 parastyle, and the relatively longer and lower anterior branch of the paralophid on m1–3. Differs from *Uintaceras* by the more lingually appressed M3 metacone, and the more oblique protolophid and hypolophid of m1–3. Differs from *Forstercooperia* by M3 less triangular in outline with a reduced metacone.

**Comparisons.** The mandible with m1–3 (IVPP V 26249) was unearthed from the same quarry (east of ‘chalicothere quarry’) where M3 (V 26250) was found; the quarry also bears a new species, possibly of *Hyrachyus* (V 26253), as described below. The juvenile mandibles (V 26251) were unearthed from the ‘chalicothere quarry’.

*Gobioceras* is distinguishable from *Hyrachyus* in having a strong cristid obliqua with a high contact with the protolophid on the lower molars, and a reduced, more lingually placed metacone on M3 with a triangular outline. All these features suggest its affinity with rhinocerotoids. However, the parastyle of M3 still remains relatively large as in *Hyrachyus* and tapiroids, but is somewhat more compressed as in rhinocerotoids. The roughly triangular outline of M3 with reduced, lingually appressed metacone excludes its affinity with amynodontids. Furthermore, the M3 metacone of *Gobioceras* is relatively more lingually placed and smaller than those of *Triplopus* that have rudimentary metacones^9^. The lower molars of *Gobioceras* are similar to those of *Triplopus* in having oblique transverse lophids, but different from the latter by having a more U-shaped outline of trigonids with longer paralophids, the cristid obliqua of m1–3 descending slightly rather than sharply from the hypoconid, and joining the protolophid in a relatively higher position based on the slightly worn teeth^9^. The m1–3 of *Gobioceras* is further different from Asian *Triplopus*? *proficiens* in having a less lingually extended cristid obliqua which has an angled joint with the hypolophid. The lower molar length of *Gobioceras* (63.1 mm) is considerably larger than in species of *Triplopus*, although the former from the early Arshantan (roughly equivalent to the early Bridgerian NALMA) is much earlier than Irdin Manhan (or equivalent to the Uintan NALMA) *Triplopus*^9,18^. Compared with *Triplopus*? *youjingensis* from the same horizon, *Gobioceras* is larger and has a U-shaped trigonid on the lower molars and a more oblique protolophid and hypolophid. Furthermore, *Gobioceras* differs from *Prohyracodon*^48,49^ in having a less reduced metacone, a larger parastyle on M3, and a more oblique protolophid and hypolophid on the lower molars. Thus, *Gobioceras* is remote from the ancestry of any hyracodontid rhinoceroses.

Among rhinocerotoids, only *Pappaceras*, which consists of three species, has been reported from the upper part of the Arshanto Formation^11,18,50,51^. *Pappaceras* was considered to be closely related to *Forstercooperia* from the overlying Irdin Manha Formation^20,52^, which gave rise to later *Juxia* and other giant rhinos^13^. It is not surprising to note that *Gobioceras* from the base of the Arshanto Formation is considerably smaller than *Pappaceras* from the higher horizon (Fig. 4c, d). However, lower molars of *Gobioceras* show some similarities with those of *Pappaceras* in having a generally U-shaped trigonid, oblique protolophid and hypolophid that parallel each other, a smoothly curved joint at the hypoconid, and a cristid obliqua contacting the protolophid in a relatively high position. But *Pappaceras* is more advanced than *Gobioceras* in having a higher crown, a relatively shorter and higher anterior branch of the paralophid on m1–3, and the buccal branch of the paralophid of m1 slightly more lingually extended. The M3 parastyle of *Gobioceras* is relatively larger and more cuspate than that of *Pappaceras*, but both of them are strongly buccally projected relative to the paracone. The M3 metacone of *Gobioceras* is as lingually placed as those in *P. confluens* and *P. minuta*, but that of *P. meiomenus* is obviously more buccally situated. The M3 metacone of *Gobioceras* is more distinct than that of *P. confluens*, but less prominent than those of *P. minuta* and *P. meiomenus*, which are even buccally deflected. However, the prominence of metacone on M3 may be a variable character as inferred from *Uintaceras*^9,19^ and *Teletaceras*^53^. To sum up, *Gobioceras* is closely related to *Pappaceras* and probably represents the ancestral condition for the latter. *Forstercooperia* from the overlying Irdin Manha Formation (or equivalent Ulan Shireh Formation) is distinguished by a much larger size, and a more triangular outline of M3 without a metacone^20^.

The Uintan *Uintaceras radinskyi*, which is considered to be a sister group of Rhinocerotidae^9,19^, also bears a subtriangular M3 with nearly confluent centrocrista and metaloph, a relatively large parastyle, and a reduced metacone as in *Gobioceras*. But *Uintaceras* (m1–3 length: 88–93)^9^ is considerably larger than *Gobioceras*. *Uintaceras* is further different from *Gobioceras* in having the M3 metacone less lingually placed, and the protolophid and hypolophid of m1–3 more transversely extended.

? Hyracodontidae Cope, 1879

*Ephyrachyus* Wood, 1934

**Type species.** *Ephyrachyus implicatus*

**Included species.** *E. cristalophus*, and *E. woodi* sp. nov.

**Localities and horizons.** Middle Eocene; Washakie Formation of the Washakie Basin, Bridger C_3_ of the Bridger Basin, Wyoming, US; upper part of the Arshanto Formation, Erlian Basin, Inner Mongolia, China.

**Diagnosis.** Upper cheek teeth with the paracone and metacone more merged to form the ectoloph; P3–4 with a high metaconule and a relatively long endoprotocrista. Differs from *Hyrachyus* and *Metahyrachyus* (*sensu* Wood, 1934) by having the paracone and metacone merged with the ectoloph on the upper cheek teeth, the P3–4 metaconule relatively high, and the endoprotocrista relatively long. Further differs from *Metahyrachyus* (*sensu* Wood, 1934) by the protocone not joining the metaconule on P2, and the hypocone not budding off from the endoprotocrista on P3–4.

*Ephyrachyus* *woodi* sp. nov.

**Fig. 6:**
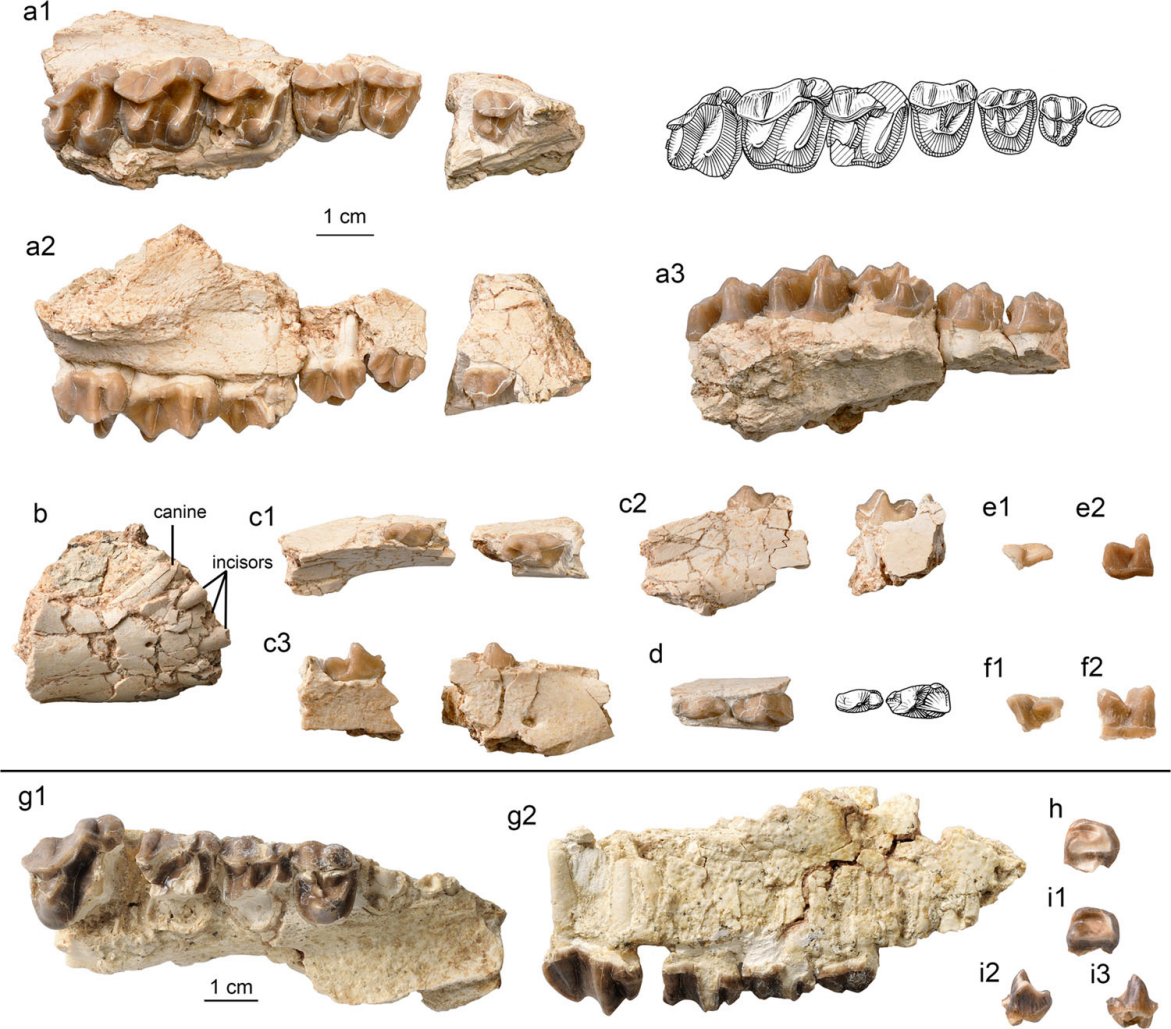
Specimens of *Ephyrachyus woodi* sp. nov. and *Hyrachyus*? *tumidus* from the Arshanto Formation of the Erlian Basin. **a**–**f** *E. woodi* (IVPP V 26252, holotype), **a** right maxilla with P2–M3 in occlusal (a1), buccal (a2), and lingual (a3) views; **b** fragmentary symphyseal region with the roots of incisors and canine; **c** right mandible with p2–3 in occlusal (c1), lingual (c2), and buccal (c3) views; **d** left mandible with p2–3 in occlusal view; **e** p4 fragment in occlusal (e1) and buccal (e2) views; **f** m1/2 fragment in occlusal (f1) and buccal (f2) views. **g**–**i** H.? *tumidus*; **g** right maxilla with P3–M2 (IVPP V 26253.1, holotype) in occlusal (g1) and buccal (g2) views; **h** m1 fragment (IVPP V 26253.2) in occlusal view; **i** m2 fragment (IVPP V 26253.3) in occlusal (i1), buccal (i2), and lingual (i3) views.

**Holotype.** IVPP V 26252, a right maxilla with P2–M3, right and left p2–3, and fragmentary p4 and lower molar (Fig. 6a–f).

**Etymology.** The specific name honors H. E. Wood, who erected the genus *Ephyrachyus* and made a thorough revision of hyrachyids from North America in 1934.

**Locality and horizon.** Early–middle Eocene, lower horizon of the upper part of the Arshanto Formation, Chaganboerhe.

**Diagnosis (Table** **2****and Supplementary Note** 1**).** Differs from both *E. implicatus* and *E*. *cristalophus* in having the endoprotocrista of P3–4 posterobuccally extended from the protocone at a sharp angle, metaconules of P2–4 transversely extended; the metaconule of P4 not fused with the crista; M1–3 parastyle relatively larger. Further differs from *E. implicatus* by having relatively narrower and longer upper molars with more lingually placed metacones, and by lacking a posterior cingulum on P2 curved up on to the protocone. Further differs from *E*. *cristalophus* by having a metaconule on P2, and a relatively shorter M3 metaloph not confluent with the centrocrista.

**Comparisons.** The new specimens clearly show some ‘*Hyrachyus*’-like characters, including large parastyles closely appressed to the paracones on the upper molars, relatively long postmetacrista on M1–2, M3 metacone reduced, buccally deflected, and perpendicular to the metaloph, and a relatively low cristid obliqua on the lower molars. The length of M1–3 is about 43.2 mm, which is similar to that of *Hyrachyus modestus* (*s.l*.) with the mean length ranging from 45 to 50 mm^16^. However, the upper cheek teeth with paracones and metacones merged to form ectolophs, and the relatively high metaconules on P3–4 resemble those of *Ephyrachyus* erected by Wood in 1934^15^.

The type of *Ephyrachyus* was based on ‘*Hyrachyus*’ *implicatus* (AMNH FM 5078), which was unearthed from the probably late Bridgerian of the Washakie Formation in the Washakie Basin, Wyoming^15,54^. Wood also erected a new species *E. cristalophus* from the Bridger C_3_ (=late Bridgerian) in the Bridger Basin, Wyoming^15,55^. However, Radinsky^16^ assigned *E. implicatus* to *Triplopus* mainly based on its occurrence in the Washakie Formation, from which *Hyrachyus* is unknown; by contrast, *Hyrachyus* are much more abundant in the Bridger Formation. Radinsky^9^ further considered *Eotrigonias petersoni* to be a synonym of *T. implicatus*. Radinsky^16^ also considered *E. cristalophus* to be a synonym of *H. modestus*, representing a small sized species of late Bridgerian *Hyrachyus*.

The new material, preserving nearly complete P2–M3 from the Erlian Basin, suggests that *Ephyrachyus* is a valid genus and ‘*Eotrigonias petersoni*’ is not a synonym of ‘*Ephyrachyus*’ *implicatus* (Supplementary Note 1). The new material is similar to ‘*Ephyrachyus*’ *implicatus* in having a prominent metaconule on P2 separated from the protoloph, paracones and metacones merged with the ectolophs on P2–4, endoprotocristae of P3–4 relatively long, metaconules of P3–4 high and enclosing the medifossette, and p3 with a distinct paraconid and lacking the entoconid. These similarities suggest that the new material and ‘*Ephyrachyus*’ *implicatus* should be assigned to the same genus. The new material can be distinguished from ‘*Ephyrachyus*’ *implicatus* by the lacking a posterior cingulum on P2 curved up on to the protocone, and in having the endoprotocristae of P3–4 posterobuccally rather than posterolingually extended from the protocone with sharp angles, metaconules of P2–4 transversely rather than posterolingually extended, and the metaconule of P4 not fused with a crista. The lower cheek teeth of the new material are more primitive than those of ‘*Ephyrachyus*’ *implicatus* in having the metaconid of p3 placed close to the protoconid, and a relatively lower cristid obliqua. Compared with M1–3 of CM 9384, which was assigned to ‘*Triplopus*’ *implicatus* by Radinsky^9^, those of the new material are different in being relatively narrower and longer, and in having larger parastyles and more lingually placed metacones. Although M3 of the holotype of ‘*Ephyrachyus*’ *implicatus* is fragmentary, both the new material and CM 9384 show a reduced metacone of M3 buccally deflected and perpendicular to the metaloph, which are characteristics of hyrachyids rather than *Triplopus*. Thus, ‘*Ephyrachyus*’ *implicatus* should not be reassigned to *Triplopus*, and we suggest resurrecting *Ephyrachyus* for those advanced, small ‘hyrachyids’. The new material represents a new species, *E. woodi*, first known from Asia.

Compared with *Ephyrachyus*, the holotype of ‘*Eotrigonias*’ *petersoni* (AMNH FM 2341) is distinguishable by smaller parastyles on P4–M3, metacones of M1–2 flatter and more elongated, and metacone of M3 relatively longer and lingually deflected^47^. Thus, ‘*Eotrigonias*’ *petersoni* is not a synonym of ‘*Ephyrachyus*’ *implicatus*, but probably represents a valid species *T. petersoni*.

Another species of *Ephyrachyus*, *E*. *cristalophus*, was considered to be a synonym of *H. modestus*^16^. However, *E*. *cristalophus* is similar to both *E. woodi* and *E. implicatus* in having the paracones and metacones merged to form ectolophs on P2–4, relatively long endoprotocristae and high metaconules on P3–4, and elongated metacones on M1–2. We follow Wood^15^ in considering *E*. *cristalophus* as a valid species of *Ephyrachyus*. The new material is different from *E*. *cristalophus* in having a metaconule on P2, the endoprotocristae of P3–4 sharply rather than smoothly curved from the protocones, metaconules of P3–4 transversely extended and enclosing the medifossette, the metaconule of P4 not fused with the crista, and the metaloph of M3 relatively shorter and not confluent with the centrocrista. The dental morphology of *E. woodi* is somewhat intermediate between those of *E. cristalophus* and *E. implicatus*, but is more similar to the latter. Furthermore, the similarities between *E. woodi* and North American *E. implicatus* indicate that the age of the upper part of the Arshanto Formation can be correlated to the late Bridgerian (Br3).

Two species of *Hyrachyus* have been reported from the Arshanto Formation in the Erlian Basin^39^: *H. neimongoliensis* and *H. crista*. *H. neimongoliensis* is preserved by a fragmentary skull with P3–M3 (IVPP V 5721), and Huang and Wang^56^ have argued its probable affinity with amynodontids. Although Qi^39^ assigned it to *Hyrachyus*, he also noticed that its cranial morphology and size resembles those of *P. confluens* (=*Forstercooperia huhebulakensis*)^20,50^. We consider ‘*Hyrachyus neimongoliensis*’ likely to be a synonym of *P. minutus* or *P*. *meiomenus* (Supplementary Note 1). If the latter case is true, the specific name *P*. *neimongoliensis*^39^ has priority over *P*. *meiomenus*^11^.

Another species of *Hyrachyus*, *H. crista*, was reported from the Arshanto Formation at Bayan Ulan^39^. *H. crista* is different from *E. woodi* in being larger, and in having a more distinct paracone rib on P4, a metaconule of P4 not in contact with the single protocone on the lingual side, parastyles of molars relatively more reduced, the protocone more anteriorly placed related to the level of the paracone on M1–3, the metacone ribs faint or absent on M1–2, the crista (not crochet as described in the context of Qi^39^) more distinct on M1–3, and the metacone of M2 much more elongated.

Radinsky^30^ reported Cf. *Hyrachyus* (AMNH FM 81801) with P4–M3 from the Arshanto Formation at Huheboerhe in the Erlian Basin. *E. woodi* is different from Cf. *Hyrachyus* in having metacone more separated from the paracone on P4, paracone and the metacone of P4 more merged with the ectoloph, the hypocone not separated from the protocone on P4, a distinct metacone rib on M2, and a relatively larger parastyle on M1–3. AMNH FM 81801 probably represent a new species of *Hyrachyus* as suggested by Huang and Wang^56^.

Hyrachyidae Osborn, 1892

*Hyrachyus*? *tumidus* sp. nov.

**Holotype.** IVPP V 26253.1, a right maxilla with broken P3–M2 (Fig. 6g).

**Referred specimens.** IVPP V 26253.2, .3, trigonids fragments of lower molars (Fig. 6h, i).

**Etymology.** The Latin ‘*tumidus*’ means swollen, referring to the swollen buccal surface of the P3–4 paracone and metacone.

**Locality and horizon** Late early Eocene, basal part of the Arshanto Formation, Nuhetingboerhe.

**Diagnosis (Table** **2****and Supplementary Note** 1**).** Differs from other species of *Hyrachyus* by the combination of following characters: P3–4 paracone and metacone rounded and swollen on the buccal surface; P3 with a long endoprotocrista and a metaconule directed to the base of the protocone; M1–2 with a parastyle somewhat separated from the paracone, a prominent metacone rib, and a relatively short postmetacrista.

**Comparisons.** The new material has the following characters suggestive of *Hyrachyus* affinity^9,41^: a prominent metacone rib on M1–2, a relatively long postmetacrista, a weak cingulum on the buccal side of the metacone, a strong, cuspate parastyle on M1–2, and the attachment between the metaconule and the ectoloph higher than the corresponding attachment between the protoloph and ectoloph on P4. Compared with other known species of *Hyrachyus* (Wood’s^15^ *H. modestus* and *H. affinis*) from early and middle Bridgerian (Br1–2, approximately equal to Bridger A and B) of North America, *H*.? *tumidus* shows some relatively advanced features, including a protocone posteriorly extended on P3, a high, compressed parastyle on P4, a high and sharp paracone on M1–2 with the parastyle somewhat separated from the paracone. These features are in turn more or less reminiscent of Wood’s^15^ *H. eximius* and ‘*Colonoceras agrestis*’ from the late Bridgerian (Br3, Bridger C-D)^15,16,55^. Compared with hyrachyids from the late Bridgerian, *Hyrachyus*? *tumidus* is more advanced than *H*. ‘*princeps*’ in having more molarized P3, but more primitive than ‘*Metahyrachyus*’ in lacking the hypocones on P3–4^15^. Furthermore, the upper cheek teeth of *H*.? *tumidus* is usually larger than those of *Hyrachyus* from the middle Bridgerian, and approaches the relatively larger size in hyrachyids from the late Bridgerian^15,16,55^. Thus, *H*.? *tumidus* seems more similar to species of *Hyrachyus* from the late Bridgerian of North America than those from early and middle Bridgerian. However, the fragmentary material and lack of M3 and most of the lower dentition in the new species make this statement very provisional. Compared with *H. metalophus*^57^ from Shandong Province, both have distinct metacone ribs on M1–2, but *H*.? *tumidus* can be distinguished by larger parastyles and shorter metacones on M1–2.

It is noteworthy that the buccal surfaces of the paracone and metacone on P3–4 are rounded and swollen rather than the rib-like as in other species of *Hyrachyus*. These features are in turn similar to those of *Uintaceras radinskyi*, which Holbrook and Lucas^19^ considered to be the sister taxon of Rhinocerotidae^19^. In addition, *H*.? *tumidus* also resembles *Uintaceras* in having a posteriorly extended protocone on P3 with the metaconule directed toward the base of protocone, and a relatively short postmetacrista on M1–2 with more separated parastyle. These similarities probably indicate that *H*.? *tumidus* has a close relationship with *Uintaceras*. However, because of the lack of M3 and complete material, we tentatively assign the species to *Hyrachyus*, pending the new discovery of more complete material in the future.

### The phylogenetic analysis

**Fig. 7:**
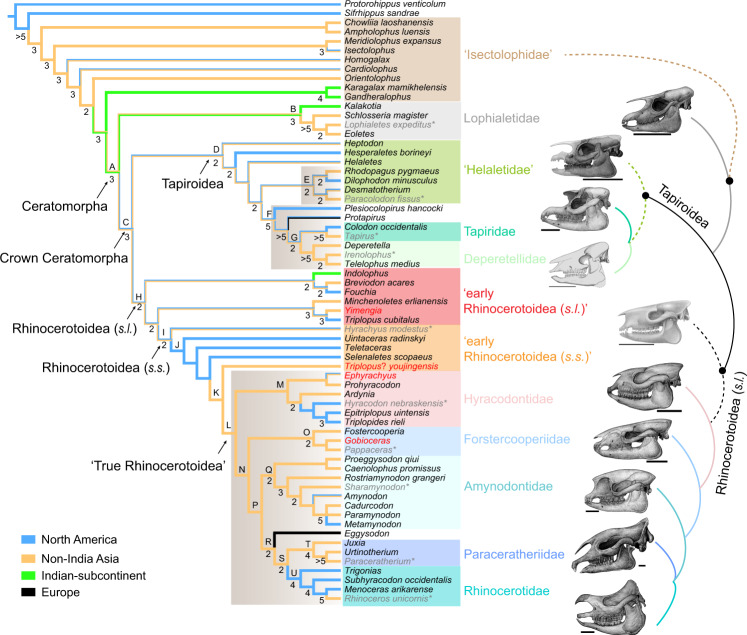
The strict consensus tree of two most parsimonious trees, showing the phylogeny of Ceratomorpha with paraphyletic ‘Isectolophidae’ as a sister group. All new taxa reported here are marked in red, and placed in Rhinocerotoidea. The geographic distribution was reconstructed using the parsimony criterion in Mesquite^89^. The taxa marked in gray with asterisks in different clades are reconstructed on the right side with simplified phylogenetic relationships (scale bar equals 10 cm). The numbers and letters at the nodes show Bremer Support >1, and the clades discussed in the text with synapomorphies are listed in Supplementary Table 1.

A cladistic analysis with parsimony criteria results in two equally most parsimonious trees (MPTs). The tree length of the strict consensus is 2765; the consistency index is 0.234; the retention index is 0.497. The cladogram of the strict consensus tree shows two main clades of Ceratomorpha: Tapiroidea and Rhinocerotoidea; however, the endemic Asian Lophialetidae is a stem group of Ceratomorpha (Fig. 7). Regarding the new materials of rhinocerotoids reported here, *Yimengia* is placed within Rhinocerotoidea (*s.l*.), and is a sister group to *T. cubitalus*, which has been considered as an early hyracodontid by Radinsky^9^. *Minchenoletes* forms a sister group to the *Yimengia* and *T. cubitalus* clade. *Triplopus*? *youjingensis* is most closely related to the ‘true rhinocerotoids’, which comprises Hyracodontidae, Amynodontidae, ‘Paraceratheriidae’, and Rhinocerotidae. *Epihyrachyus* is a sister group to *Prohyracodon*, and both allied with Hyracodontidae. *Gobioceras* is a sister group to *Pappaceras*, and they are allied with *Forstcooperia*. Forstercooperiidae forms a clade as a sister group to the clade comprising Amynodontidae, ‘Paraceratheriidae’, and Rhinocerotidae.

**Fig. 8:**
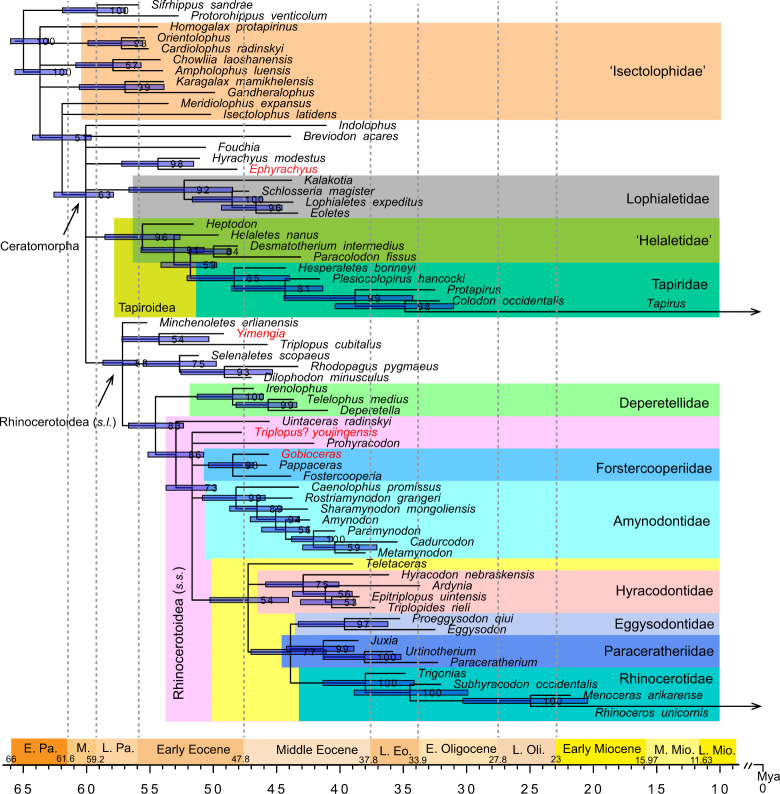
Majority-rule (50%) consensus trees of Ceratomorpha using Bayesian phylogenetic tip-dating analyses. The node ages (divergence times) are the median estimates and node bars represent the 95% highest posterior density (HPD) intervals of the divergence times. The numbers at the internal nodes are the posterior probabilities of the corresponding clades. Eo. Eocene, Mio. Miocene, Oli. Oligocene, Pa. Paleocene.

The Bayesian tip-dating analysis generates a majority consensus tree, which is shown in Fig. 8. The relationships within Ceratomorpha are less resolved than in the parsimonious tree, and alternative phylogenetic positions for some taxa or groups are suggested. However, considering the taxa studied in the present paper, their phylogenetic positions generally coincide with those inferred from the parsimony analysis. *Yimengia* is the sister group to *T. cubitalus* as suggested by the parsimony analyses. *Triplopus*? *youjingensis* is placed in Rhinocerotoidea (*s.s*.) with a polytomous position (excluding *Uintaceras*). *Gobioceras* is allied with *Pappaceras* and *Forstercooperia*, but they form a trichotomous clade. *Hyrachyus*, instead of *Prohyracodon*, is the sister group of *Ephyrachyus*, and they form a clade with an unresolved position in Ceratomorpha.

## Discussion

### ‘Isectolophidae’ and Lophialetidae

The phylogenetic trees show some interesting results and resolve long-lasting controversies on the phylogeny and biogeography of Ceratomorpha, although some discrepancies are present between the MPT and the Bayesian Inference tree (BIT). It is necessary to mention that the ancestral distributions were reconstructed based on the most parsimony tree (Fig. 7). The paraphyletic ‘Isectolophidae’ originated from Asia (excluding India) in the early Eocene, and then dispersed to North America and the Indian-subcontinent (Fig. 7). The *Karagalax*–*Gandheralophus* clade is most closely related to Ceratomorpha in the MPT; however, *Meridiolophus* and *Isectolophus* are closer to Ceratomorpha than are other ‘isectolophids’ in the BIT (Fig. 8). The relatively derived position of *Meridiolophus* is consistent with its intermediate morphologies between *Homogalax*-like taxa and *Heptodon*^58^. The endemic Asian Lophialetidae is excluded from the crown Ceratomorpha and represents a stem group (Fig. 7, node B; Supplementary Table 1) in the MPT, and its phylogenetic position is similar to that in the cladogram proposed by Hooker^4^. Thus, Lophialetidae should neither be placed in Tapiroidea^30^ nor in Rhinocerotoidea^5^. The ancestral distribution of lophialetids is either in the Indian-subcontinent or in non-India Asia (Fig. 7). But Lophialetidae is placed in an unresolved position within Ceratomorpha in the BIT (Fig. 8). *Ampholophus*, originally considered as a lophialetid^59^, is a sister group of *Chowliia* in both analyses, and the clade is included in a paraphyletic ‘Isectolophidae’.

### Tapiroidea and ‘early Rhinocerotoidea’

The crown Ceratomorpha is composed of superfamilies Tapiroidea and Rhinocerotoidea in the MPT (Fig. 7, node C; Supplementary Table 1). We consider Tapiroidea to be a monophyletic group (Fig. 7, node D, 8; Supplementary Table 1), because ‘Isectolophidae’ is excluded from Tapiroidea and may also give rise to ancylopods^4,60,61^. Furthermore, rhinocerotoids do not originate from tapiroids, but probably from ‘isectolophids’ and/or lophialetids. The crown Ceratomorpha originated in Asia or North America, and the ambiguity is probably attributed to the nearly simultaneous appearances of early tapiroids and/or rhinocerotoids during the early Eocene on both continents (Fig. 7).

The superfamily Tapiroidea is supported by several common synapomorphic characters in the MPT (Supplementary Table 1), such as M1 postmetacrista considerably posterobuccally oriented (105:2), cristids obliquae of lower molars highly reduced (234:2, 258:2) and directed toward protoconid (235:1, 260:1), and absence of nasolacrimal contact (335:0). *Heptodon* is the sister group to other tapiroids (Fig. 7, node D, 8). The conventional ‘Helaletidae’ is clearly not a monophyletic group, because both Tapiridae and Deperetellidae derived from ‘helaletids’ in the MPT, which is consistent with previous morphologic comparisons^62^. The Asian endemic Deperetellidae is more closely related to Tapiridae than to lophialetids^1^ or rhodopagids^4^ (Fig. 7, node G), and *Colodon* is closer to *Tapirus* than is *Protapirus* as suggested by Colbert^5^. Furthermore, *Rhodopagus* and *Dilophodon* form a sister group within ‘Helaletidae’, rather than being allied with rhinocerotoids. In contrast, both the *Rhodopagus*–*Dilophodon* clade and Deperetellidae are placed in Rhinocerotoidea (*s.l*.) in the BIT, and Deperetellidae is even the sister group to Rhinocerotoidea (*s.s*.) (Fig. 8). *Rhodopagus* from the middle Eocene of Asia was first included in Tapiroidea^30^, but subsequent investigations have suggested that *Rhodopagus* may be a hyracodontid^21,23^ or primitive rhinocerotoid^6,35^. Similarly, *Dilophodon* was usually considered to be a small tapiroid from the middle Eocene of North America^30,35^, but Emry^22^ suggested its sister relationship with *Fouchia* and close to rhinocerotoids^21^. However, a sister group relationship between Deperetellidae and Rhinocerotoidea (*s.s*.) is somewhat unexpected, because the former has been unequivocally placed in Tapiroidea based on its craniodental characters^30,62^. But the enamel microstructure found in the molars of Deperetellidae are characterized either by vertical HSB (Hunter-Schreger Band) or by compound HSB, which has been seen in unequivocal Rhinocerotoidea, and ‘Hyrachyidae’ and *Uintaceras*, respectively^62,63^. In contrast, the enamel microstructure found in the cheek teeth of Tapiroidea have either a transversal HSD or a curved HSD^63^.

The superfamily Rhinocerotoidea (*s.l*.) is supported by several common synapomorphic characters in the MPT (Supplementary Table 1), such as P3–4 postprotocrista absence (49:0, 75:0), M1–2 protolophid somewhat posterolingually oblique (227:1), M1–2 metaconid slightly more posteriorly displaced to the protoconid (228:1), and m3 hypolophid slightly posterolingually oblique (263:1). Beside *Yimengia* and Deperetellidae, some taxa previously allied with Tapiroidea are replaced in Rhinocerotoidea (*s.l*.) in both analyses (Fig. 7, node H, 8; Supplementary Table 1). Those taxa include *Minchenoletes* from the early Eocene of Asia^37^, and *Selenaletes* from the early Eocene of North America^43^. *Minchenoletes* is either a sister group to the *Yimengia* and *T. cubitalus* clade (in the MPT) or placed in an unresolved position in Rhinocerotoidea (*s.l*.) (in the BIT), instead of being a primitive lophialetid as originally assigned^37^. *Selenaletes* was initially considered to be a helaletid^43^, but it is placed either in a sister group to the ‘True Rhinocerotoidea’ plus *Triplopus*? *youjingensis* (in the MPT) or forms a sister group to the *Rhodopagus*–*Dilophodon* clade (in the BIT). In the parsimony tree, *Indolophus* forms a sister group to the *Breviodon* and *Fouchia* clade, and together they represent a sister group to other Rhinocerotoidea (*s.l*.). *Fouchia* was originally considered to be in a pivotal position to the origin of rhinocerotoids^22^, and the statement is supported by the present cladogram. However, *Indolophus*, *Breviodon*, and *Fouchia* are polytomous in Ceratomorpha based on the BIT. *H. modestus* is a sister group to other Rhinocerotoidea (*s.s*.) in the MPT (Fig. 7), but forms a sister group to *Ephyrachyus* and they are together placed in an unresolved position in Ceratomorpha in the BIT (Fig. 8).

### ‘True Rhinocerotoidea’

The phylogenetic trees further provide the phylogenetic relationships among four ‘true rhinocerotoid’ families (Fig. 7, node L, 8). In the MPT, Hyracodontidae is a sister group to other ‘true Rhinocerotoidea’, and originated from non-India Asia. It is a monophyletic group if the genus *Triplopus* is excluded from hyracodontids (Fig. 7, node M). *Ephyrachyus* is the sister group to *Prohyracodon*, and is remote from *Hyrachyus*. The *Ephyrachyus* and *Prohyracodon* clade forms a sister group to other hyracodontids. In contrast, Hyracodontidae, which excludes *T. cubitalus* and *Prohyracodon*, is more closely related to the Eggysodontidae–Paraceratheriidae–Rhinocerotidae clade in the BIT (Fig. 8). The Asian endemic Paraceratheriidae (*s.l*.), usually comprising Forstercooperiinae and Paraceratheriinae, is not a monophyletic group^11,13^ in both analyses. The Forstercooperiidae (Fig. 7, node O) is a sister group to other ‘true rhinocerotoids’ except for hyracodontids in the MPT, and its phylogenetic position is somewhat similar to that proposed by Holbrook^7^. However, Forstercooperiidae is placed in a polytomous position in Rhinocerotoidea (*s.s*.) (excluding *Uintaceras*) in the BIT. In the MPT, Paraceratheriidae (*s.s*.), which is represented by *Juxia*, *Urtinotherium*, and *Paraceratherium*, is most closely related to Rhinocerotidae, as proposed by Heissig^8^ (Fig. 7, node S), rather than being closely related either to hyracodontids^6,9,64^ or amynodontids^11,20^. Current evidence suggests that Rhinocerotidae likely originated from North America. The Rhinocerotidae clade is supported by several synapomorphic characters, including a chisel-like I1 (3:3) and a tusk-like i2 (144:3) (Supplementary Table 1), which were usually considered to be the most conspicuous features of Rhinocerotidae^12^. The lack of metacone on M3 (125:3) is not restricted in rhinocerotids, and is also distributed in other rhinocerotoids except for Amynodontidae, which is characterized by a distinct metacone on M3 (125:1) with a short postmetacrista (127:0) (Supplementary Table 1). *Eggysodon* is the sister group to the Paraceratheriidae and Rhinocerotidae clade in the MPT (Fig. 7). Amynodontidae is a sister group to the *Eggysodon*–Paraceratheriidae–Rhinocerotidae clade (Fig. 7, node P), and originated from non-India Asia. *Proeggysodon*, previously considered to be a primitive eggysodontid, forms a sister group to *Caenolophus promissus*, and both of them represent a sister group to other amynodontids in the MPT. *Caenolophus* was originally considered to be a hyracodontid^65^, but later became allied with amynodontids^6,9^. *Proeggysodon* was known only from a mandible and the lower dentition^66^, which probably bias its phylogenetic position in the MPT. In contrast, Eggysodontidae (*Eggysodon* and *Proeggysodon*), Paraceratheriidae, and Rhinocerotidae form a trichotomous clade in the BIT, and the phylogenetic position of Amynodontidae within Rhinocerotoidea (*s.s*.) (excluding *Uintaceras*) is unresolved (Fig. 8).

### The discrepancy between the most parsimonious and BITs

As discussed above, the general topologies are somewhat different between the MPT and BIT (Figs. 7 and 8). Lophialetidae is a stem group of Ceratomorpha in MPT, but placed in an unresolved position in Ceratomorpha in BIT. However, the phylogenetic positions of some lineages are contradicted between the two methods. The *Rhodopagus*–*Dilophodon* clade and Deperetellidae are placed in Tapiroidea in the MPT, but both are allied with Rhinocerotoidea (*s.l*.) in the BIT. Amynodontidae is closer to the Eggysodontidae–Paraceratheriidae–Rhinocerotidae clade than is Hyracodontidae in the MPT; however, the BIT suggests a closer relationship between the latter two clades. The preference of different topologies generated by the parsimony and Bayesian analysis for morphological data are ongoing debate^67,68^, and it seems that both have advantages and disadvantages for morphological data^69,70^. The parsimony method only provides a point estimate (the MPTs) while Bayesian inference averages over the uncertainties of the topologies by summarizing a majority-rule consensus tree. Moreover, the Bayesian tip-dating analysis takes both the morphological characters and geological times into account and models the diversification and sampling processes explicitly, while the parsimony method uses morphological characters solely and absents explicit model assumptions. Nevertheless, the taxa or clade contradictory in both methods indicate that the data might not contain enough information to draw firm conclusions about their relationships^71^. With more fossils and more complete data added in the matrix in combination with improvements of algorithms and parameters^70,72,73^, two methods probably converge to more compatible results.

### The divergence of Ceratomorpha

The new rhinocerotoid taxa *Y. magna*, as well as reassigned *Minchenoletes*, from the early Eocene Bumbanian is nearly contemporary with early Eocene tapiroids, suggesting that the divergence between rhinocerotoids and tapiroids occurred no later than the early Eocene (52–56 Mya). The divergence time between Rhinocerotoidea and Tapiroidea in the early early Eocene based on fossil evidence here falls between the ~51 Mya and ~57.5 Mya estimates from molecular data^2,3^. Furthermore, forstercooperiid *Gobioceras*, rhinocerotoid *Triplopus*? *youjingensis*, and rhinocerotid-like *Hyrachyus*? *tumidus* from the base of the Arshanto Formation suggest that divergence of these different rhinocerotoid groups occurred no later than the late early Eocene, soon after the split between the rhinoceroses and the tapiroids. However, the Bayesian tip-dating estimate suggests that the median value of the divergence time of different ceratomorph groups (60.1 Mya) is in the middle Paleocene, and that of rhinocerotoid groups (*s.l*.) (57.2 Mya) is in the late Paleocene (Fig. 8). Both estimates are earlier than current fossil evidence, but the former estimate is close to the divergence time between Rhinocerotoidea and Tapiroidea (57.5 Mya) based on recent molecular analysis^3^. Similarly, the divergences time of different groups within Lophialetidae, Tapiroidea, and Rhinocerotoidea (*s.s*.) are in the early Eocene, and the divergence between Deperetellidae and Rhinocerotoidea (*s.s*.) occurred 54.6 Mya (Fig. 8). The divergences of the groups within Forstercooperiidae and Amynodontidae occurred in the late early Eocene, while those of the groups within Hyracodontidae, Eggysodontidae, Paraceratheriidae, and Rhinocerotidae occurred in the middle Eocene. The median value of the divergence time of Eggysodontidae, Paraceratheriidae, and Rhinocerotidae is 43.9 Mya (95% HPD = 41.1–47.0 Mya).

The diverse rhinocerotoids from the base of the Arshanto Formation are probably correlated with the Early Eocene Climatic Optimum^18,74^ and likely lived in a relatively close, humid environment as inferred from the dental stable carbon isotope analyses of *Schlosseria* from the same horizon^75,76^. The habitat of Lophialetidae in the Huheboerhe area is considered to be ‘a relatively open forest environment like a woodland (or a low-density forest)’, and became relatively more arid and/or open over time during the early–middle Eocene^75,76^.

## Conclusions

To sum up, the phylogenetic analysis based on both parsimony and Bayesian inference criteria highlights the phylogeny and biogeography of Ceratomorpha, especially for some long-standing controversial groups, such as lophialetids, deperetellids, equivocal early rhinocerotoids, and relationships among rhinocerotoid groups. Both Tapiroidea and Rhinocerotoidea are independent, monophyletic groups, and derived from ‘isectolophids’ and/or lophialetids. Lophialetidae is a stem group of Ceratomorpha in the MPT. Some taxa conventionally assigned to tapiroids are placed to Rhinocerotoidea (*s.l*.). However, the phylogenetic positions of Deperetellidae, the *Rhodopagus*–*Dilophodon* clade, Hyracodontidae, and Amynodontidae within Ceratomorpha are controversial between the two methods. Furthermore, we propose that the divergence between the Rhinocerotoidea and Tapiroidea occurred no later than the early early Eocene, or extended to the middle Paleocene as suggested by the Bayesian tip-dating estimate. The appearance of various rhinocerotoids from the base of the Arshanto Formation suggest that the divergence of different rhinocerotoid (*s.s*.) groups occurred no later than the late early Eocene, or in the early early Eocene as inferred from the Bayesian tip-dating estimate. The habitat of diverse rhinocerotoids from the base of the Arshanto Formation is inferred to have been a relatively close, humid environment. More groups and postcranial characters need to be added into the matrix in future investigations, in order to resolve some controversial issues and illuminate the evolutionary history of the order Perissodactyla.

## Methods

### Taxa and characters selection

The data matrix consists of 65 taxa and 361 morphological characters, including 271 dental, 77 cranial, and 13 mandibular characters (Supplementary Note 2 and 3). Early Eocene equoid *Sifrhippus* and *Protorohippus* were chosen as outgroups. The ingroup includes representatives of conventional tapiroid (i.e. ‘Isectolophidae’, ‘Helaletidae’, Tapiridae, Lophialetidae, and Deperetellidae) and rhinocerotoid (i.e. Hyracodontidae, Amynodontidae, ‘Paraceratheriidae’, and Rhinocerotidae) families. The extant species *Tapirus indicus* and *Rhinoceros unicornis* were also added in the matrix. The new taxa of rhinocerotoids reported here are included in order to test their phylogenetic positions within Ceratomorpha. The dental terminology mentioned in the text is modified from Hooker^4^ (Supplementary Fig. 1).

### Parsimony analyses

The phylogenetic analyses were conducted on TNT 1.5 using the New Technology Search method^77,78^. All characters are unordered and equally weighted. We used sectorial search, 200 ratchet iterations, 100 drifting cycles, and 10 rounds of tree fusing combined^79,80^.

### Bayesian analyses

The Bayesian tip-dating analysis was conducted by MrBayes 3.2.8^81–83^. For the substitution models, the Mkv model^84^ was used with an assumption of gamma rate variation across characters. The independent gamma rate^85^ was applied to the relaxed clock model, and the mean clock rate was assigned a gamma (2, 50) prior and the variance parameter was set to exp (1). Fossil ages (as represented by the genera) were calibrated with uniform distributions, and the minimum and maximum ages were inferred from the Land Mammal Ages^25,86^ and the Paleogene Geologic Time Scale^26^ (Supplementary Table 2). The fossilized birth–death process^87^ was used as a tree prior on branch lengths with diversified sampling^88^. The percentage of extant species sampled in the analysis was set to 0.2 (two out of ten species). The net diversification rate prior was set to exp (100). The relative extinction and fossilization priors were set to beta (1.0, 1.0). The root age was given a uniform distribution from 56 to 66 Mya. Markov chain Monte Carlo analysis consists of two independent runs and four chains (one cold and three hot) per run for 50 million iterations and sampled every 200 iterations, with a burn-in percentage of 25%.

### Nomenclatural acts

This published work and the nomenclatural acts it contains have been registered in ZooBank, the proposed online registration system for the International Code of Zoological Nomenclature (ICZN). The ZooBank LSIDs (Life Science Identifiers) can be resolved and the associated information viewed through any standard web browser by appending the LSID to the prefix ‘http://zoobank.org/’. The LSIDs for this publication is: urn:lsid:zoobank.org:pub:52AB0E77-2D01-43D0-BBA1-FA231A3E10E4.

### Reporting summary

Further information on research design is available in the Nature Research Reporting Summary linked to this article.

## Supplementary information

Supplementary Information

Reporting Summary

## Data Availability

All data needed to evaluate the conclusions in the paper are present in the paper and/or the Supplementary Information. The data matrix was deposited in Morphobank (project 3617).
