# Author Correction: The origin of Rhinocerotoidea and phylogeny of Ceratomorpha (Mammalia, Perissodactyla)

**DOI:** 10.1038/s42003-021-01660-x

**Published:** 2021-01-20

**Authors:** Bin Bai, Jin Meng, Chi Zhang, Yan-Xin Gong, Yuan-Qing Wang

**Affiliations:** 1 Key Laboratory of Vertebrate Evolution and Human Origins of Chinese Academy of Sciences, Institute of Vertebrate Paleontology and Paleoanthropology, Chinese Academy of Sciences, Beijing, 100044 China; 2 CAS Center for Excellence in Life and Paleoenvironment, Beijing, 100044 China; 3 Division of Paleontology, American Museum of Natural History, New York, NY 10024 USA; 4 Earth and Environmental Sciences, Graduate Center, City University of New York, New York, NY 10016 USA; 5 College of Earth and Planetary Sciences, University of Chinese Academy of Sciences, Beijing, 100049 China

Correction to: *Communications Biology* 10.1038/s42003-020-01205-8, published online 14 September 2020.

In the original version of the Article ‘The origin of Rhinocerotoidea and phylogeny of Ceratomorpha (Mammalia, Perissodactyla)’, the authors had used the generic name *Gobioceras* for a late Early Eocene rhinocerotoid. Authors are now aware that this name has been preoccupied by *Gobioceras* Bogoslovskaya, 1988^1^, a Permian ammonoid. A new generic name *Gobicerops* nom. nov. is proposed to replace *Gobioceras* Bai et al. 2020. We thank Mr. Mo Hassan for bringing this issue to our attention.
