# Publisher Correction: Author Correction: The origin of Rhinocerotoidea and phylogeny of Ceratomorpha (Mammalia, Perissodactyla)

**DOI:** 10.1038/s42003-021-01852-5

**Published:** 2021-03-04

**Authors:** Bin Bai, Jin Meng, Chi Zhang, Yan-Xin Gong, Yuan-Qing Wang

**Affiliations:** 1 Key Laboratory of Vertebrate Evolution and Human Origins of Chinese Academy of Sciences, Institute of Vertebrate Paleontology and Paleoanthropology, Chinese Academy of Sciences, Beijing, China; 2 CAS Center for Excellence in Life and Paleoenvironment, Beijing, China; 3 Division of Paleontology, American Museum of Natural History, New York, NY USA; 4 Earth and Environmental Sciences, Graduate Center, City University of New York, New York, NY USA; 5 College of Earth and Planetary Sciences, University of Chinese Academy of Sciences, Beijing, China

Correction to: *Communications Biology* 10.1038/s42003-021-01660-x, published online 20 January 2021.

In the original Correction relating to the Article ‘The origin of Rhinocerotoidea and phylogeny of Ceratomorpha (Mammalia, Perissodactyla)’, we stated that ‘authors had used the generic name *Gobioceras* for a late Early Eocene rhinocerotoid. Authors are now aware that this name has been preoccupied by Gobioceras Bogoslovskaya, 1988^1^, a Permian ammonoid. A new generic name Gobicerops nom. nov. is proposed to replace Gobioceras Bai et al. 2020’.

This has been corrected to ‘authors had used the generic name *Gobioceras* for a late Early Eocene rhinocerotoid. Authors are now aware that this name has been preoccupied by *Gobioceras* Bogoslovskaya, 1988^1^, a Permian ammonoid. A new generic name *Gobicerops* nom. nov. is proposed to replace *Gobioceras* Bai et al. 2020’.

