# Supplementary material for: The origin of Rhinocerotoidea and phylogeny of Ceratomorpha (Mammalia, Perissodactyla)
Source: Commun Biol. 2020 Sep 14;3:509. doi: 10.1038/s42003-020-01205-8 (PMC7490376; doi:10.1038/s42003-020-01205-8)
Supplement: Supplementary file 1 — Supplementary Information [file 42003_2020_1205_MOESM1_ESM.pdf]

Supplementary materials

The origin of Rhinocerotoidea and phylogeny of Ceratomorpha (Mammalia, Perissodactyla)

Bin Bai[1,2*] , Jin Meng[1,3,4], Chi Zhang[1,2], Yan-Xin Gong[1,2,5], Yuan-Qing Wang[1,2,5*]

[1]Key Laboratory of Vertebrate Evolution and Human Origins of Chinese Academy of Sciences, Institute of Vertebrate Paleontology and Paleoanthropology, Chinese Academy of Sciences Beijing 100044, China; [2]CAS Center for Excellence in Life and Paleoenvironment, Beijing 100044, China; [3]Division of Paleontology, American Museum of Natural History New York 10024, USA; [4]Earth and Environmental Sciences, Graduate Center, City University of New York, New York 10016, USA; [5]College of Earth and Planetary Sciences, University of Chinese Academy of Sciences Beijing 100049, China

[*] Correspondence and requests for materials should be addressed to B.B. (email: baibin@ivpp.ac.cn), or to Y.Q.W. (wangyuanqing@ivpp.ac.cn.).

Supplementary Figure 1: Dental terminology of perissodactyls mentioned in the text and character list

Supplementary Table 1: Distributions of common synapomorphies

Supplementary Table 2: Temporal distributions of ceratomorph fossils for the Bayesian tip-dating analysis (Mya)

Supplementary Note 1: Detailed descriptions and additional discussion

Supplementary Note 2: Character list

Supplementary Note 3. Data matrix

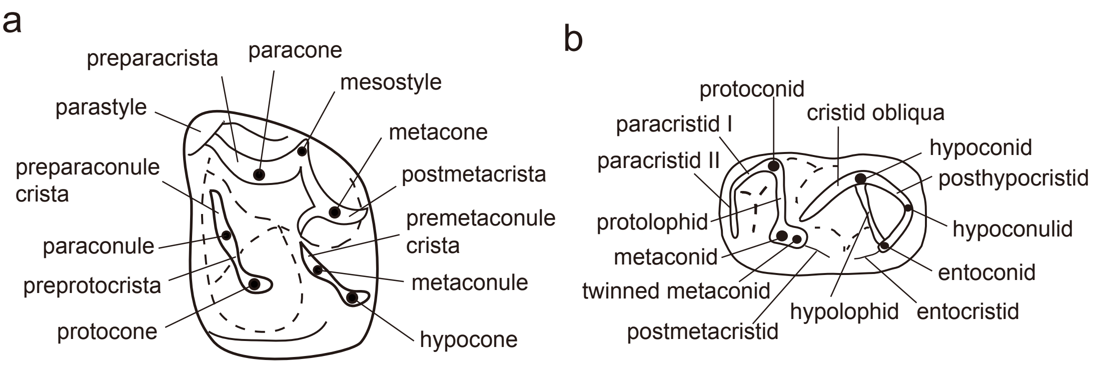

Supplementary Figure 1. Dental terminology of perissodactyls mentioned in the text and character list. **a,** diagram of upper molar; **b,** diagram of lower molar (modified from Hooker[6]).

Supplementary Table 1. Distributions of common synapomorphies (tree, fig. 7)

Node	Clade	Character and state
A	Ceratomorpha	70(1), 71(2), 85(1), 87(2), 101(0), 105(1), 106(1), 107(2), 111(1), 129(2), 133(1), 175(1), 196(1), 234(1), 262(2), 265(1)
B	Lophialetidae	37(1), 39(2), 42(1), 51(2), 63(2), 77(2), 104(2), 115(2), 117(0), 127(1), 138(1), 236(2), 245(1), 252(0), 271(1)
C	Crown Ceratomorpha	125(2), 157(0), 172(0), 295(1), 323(0), 325(0), 329(1), 354(1)
D	Tapiroidea	105(2), 169(1), 180(1), 181(1), 186(2), 221(0), 224(2), 234(2), 235(1), 258(2), 260(1), 269(1), 317(3), 335(0)
E	New clade 1	44(2) , 47(0), 49(0), 55(1), 196(0)
F	New clade 2	18(3), 23(0), 32(1), 66(0), 89(0), 189(2), 220(0), 222(1), 231(2), 343(1)
G	Tapiridae+ Deperetellidae	49(0), 51(2), 53(35), 75(0), 80(35), 103(2), 196(0), 208(2), 286(1), 349(0)
H	Rhinocerotoidea (*s.l.*)	23(0), 49(0), 75(0), 86(4), 195(3), 210(1), 227(1), 228(0), 263(1), 278(0), 281(2), 286(1)
I	Rhinocerotoidea (*s.s*)	53(1), 69(1), 74(1), 79(1), 80(1), 180(1), 234(0), 274(1), 351(2)
J	Rhinocerotoidea (*s.s*) excluding *Hyrachyus*	7(0), 12(1), 13(1), 14(1), 26(3), 29(1), 34(0), 51(2), 57(2), 77(2), 87(1), 96(2), 112(1), 127(2), 174(0), 189(1), 244(1), 269(1)
K	New clade 3	231(2), 234(1), 243(0), 249(1), 252(0), 253(1)

L	True Rhinocerotoidea	172(3), 173(1), 174(1)
M	Hyracodontidae	38(2), 62(1), 104(2), 131(1), 203(2), 205(0), 233(1)
N	New clade 4	3(2), 43(0), 94(1), 114(1), 144(2), 148(2), 189(0), 196(1), 254(3), 292(1), 293(2), 325(1), 327(1), 340(1), 343(1)
O	Forstercooperiidae	13(0), 21(2), 32(1), 55(1), 69(0), 101(3), 121(2), 122(1), 135(2), 149(1), 197(1), 216(1), 224(0), 249(0), 272(0), 298(1), 323(1), 351(1)
P	New clade 5	83(2), 99(1), 190(3), 200(0), 212(23), 215(1), 246(1)
Q	Amynodontidae	52(0), 53(0), 105(0), 106(0), 120(1), 125(1), 126(2), 127(0), 170(1), 172(0), 195(4), 213(1), 233(1)
R	New clade 6	36(0), 44(2), 60(2), 86(3), 89(1), 156(3)
S	Paraceratheriidae+ Rhinocerotidae	98(0), 149(1), 183(1)
T	Paraceratheriidae	27(1), 126(0), 141(1), 142(2), 146(0), 161(0), 189(1), 200(1), 253(0), 276(1), 295(0), 298(1)
U	Rhinocerotidae	3(3), 42(0), 53(5), 63(0), 104(2), 112(2), 138(1), 144(3), 147(1), 160(3), 173(0), 191(1), 213(1), 223(1), 259(1), 273(2), 285(2), 291(0), 292(1), 293(1), 296(2), 300(2), 302(2), 304(1), 341(0)

Supplementary Table 2. Temporal distributions of ceratomorph fossils for the Bayesian tip-dating analysis (Mya)

Taxa	Min	Max
Sifrhippus_sandrae	55.8	56.0
Protorohippus_venticolum	52.0	53.4
Orientolophus	55.0	56.0
Cardiolophus_radinskyi	52.0	56.0
Homogalax_protapirinus	52.0	55.5
Chowliia_laoshanensis	53.4	55.0
Meridiolophus_expansus	52.0	55.0
Isectolophus_latidens	39.9	52.0
Karagalax_mamikhelensis	52.0	56.0
Gandheralophus	45.0	52.0
Yimengia	45.0	52.0
Rhodopagus_pygmaeus	39.9	45.0
Indolophus	39.9	42.0
Heptodon	49.2	55.5
Helaletes_nanus	39.9	52.0
Desmatotherium_intermedius	47.8	48.5

Paracolodon_fissus	33.9	45.0
Dilophodon_minusculus	43.0	49.2
Selenaletes_scopaeus	49.2	53.4
Colodon_occidentalis	29.7	47.8
Plesiocolopirus_hancocki	39.9	43.0
Hesperaletes_borineyi	39.9	47.8
Irenolophus	45.0	52.0
Telelophus_medius	42.0	45.0
Deperetella	39.9	42.0
Minchenoletes_erlianensis	55.0	56.0
Schlosseria_magister	45.0	52.0
Lophialetes_expeditus	42.0	45.0
Eoletes	42.0	45.0
Ampholophus_luensis	53.4	55.0
Kalakotia	42.0	45.0
Breviodon_acares	42.0	45.0
Protapirus	29.7	33.9
Hyrachyus_modestus	48.5	53.0
Ephyrachyus	47.8	48.5
Fouchia	49.2	52.0
Triplopus? youjingensis	45.0	52.0
Triplopus_cubitalus	37.8	47.8
Epitriplopus_uintensis	37.8	47.8
Triplopides_rieli	36.5	37.8
Prohyracodon	33.9	45.0
Hyracodon_nebraskensis	29.0	39.9
Ardynia	23.0	37.2
Gobioceras	45.0	52.0
Pappaceras	45.0	52.0
Forstercooperia	39.9	45.0
Proeggysodon_qiui	33.9	37.2
Eggysodon	23.0	33.9
Juxia	37.2	42.0
Urtinotherium	33.9	42.0
Paraceratherium	23.0	33.9
Rostriamynodon_grangeri	42.0	45.0
Caenolophus_promissus	39.9	45.0
Amynodon	39.9	48.5
Sharamynodon_mongoliensis	39.9	45.0
Cadurcodon	30.5	37.2
Paramynodon	39.9	42.0
Metamynodon	29.7	43.0

Uintaceras_radinskyi	37.8	47.8
Teletaceras	37.8	39.9
Trigonias	32.0	37.8
Subhyracodon_occidentalis	28.0	37.8
Menoceras_arikarense	17.5	23.0

Supplementary Note 1.

Detailed descriptions

Perissodactyla

Rhinocerotoidea Gray, 1825

Family incertae sedis

Yimengia Wang, 1988

Emended diagnosis (modified from Wang[1]). small to medium size rhinocerotoids; cranium with a narial notch presumably not very deep, a large orbit, and a large, deep preorbital fossa; symphyseal region long and narrow; dental formula, 3·1·4·3/3·1·(3-4)·3; upper incisors spatulate; canine relatively small with a long postcanine diastema; premolar non-molariform with P4 metaconule joining the protocone in a relatively low position; upper molars with a small to medium parastyle slightly lingually placed to the paracone, a pinched, sharp paracone, a straight centrocrista, and a flat, relatively long, lingually situated metacone; M3 metacone reduced and lingually appressed; lower premolars without entoconid; p3 metaconid reduced and small with a slightly buccally directed paralophid; cristid obliqua high, slightly lingually directed with the buccal wall somewhat lingually tilted on p3-4; lower molars with relatively strong, anterior directed cristid obliqua, and an arched, long paralophid; m3 without the hypoconulid lobe.

Comments

Yimengia is considered to be closely related to *Rhodopagus*[1], however, the phylogenetic relationship of *Rhodopagus* is still a matter of debate. *Rhodopagus* was initially tentatively assigned to Lophialetidae with a query based on its elongated

ectoloph[2], and the assignment was supported by Gabunia and Kukhaleishvili[3]. Lucas and Schoch[4] considered *Rhodopagus* to be a hyracodontid rhinocerotoid, while Dashzeveg and Hooker[5] regarded Rhodopagidae as a rhinocerotoid, but distinct from hyracodontids. Hooker[6] proposed a sister relationship between Rhodopagidae and Deperetellidae, but the similarities were later attributed to convergence[5].

Yimengia magna sp. nov.

Holotype. IVPP V 26234, a right mandible with dp4, m1, and m3.

Locality and horizon. upper part of the Nomogen Formation, Nuhetingboerhe.

Description. P3/4 (IVPP V 26238) (Fig. 2d): The ectoloph of the tooth is broken off, but the metacone should be present as indicated by the preserved lingual part of the ectoloph. The protocone is situated at the lingual side without any trace of posterior extension. The protoloph is relatively high, joining the preparacrista and the protocone. The metaloph contacts the ectoloph between the paracone and the metacone, and as high as the joint between the protoloph and the ectoloph. The metaloph somewhat obliquely extended, joining the protocone slightly below its apex. The basin posterior the metaloph is relatively wide. The cingula are continuous at the anterior, lingual, and posterior sides, but are relatively weaker on the lingual side.

M1-2 (V 26241, fig 2f): The M1 is moderately worn with the paracone and parastyle broken off, and M2 is nearly unworn and larger than M1. The tooth is quadrate in outline. The paracone is sharp, strongly pinched on the buccal side, whereas the metacone is strongly lingually appressed with a weak rib (M1) or nearly flat (M2) on the buccal side. The parastyle is relatively strong on M2, placed anterior to the paracone, and separated from the latter. The centrocrista is straight. The postmetacrista is relatively long and posterobuccally deflected. The protocone is conical and situated at the level of the paracone. The protoloph is slightly anteriorly arched and joins the preparacrista, which is more lingually than anteriorly directed.

The metaloph is nearly as long as and parallel to the protoloph, and slightly more lingually extended than the protoloph. The metaloph joins the ectoloph at the point anterior to the apex of the metacone by a short curve. The anterior cingulum is more distinct than the posterior one, and a weak cingulum is present at the buccal base of the metacone. The anterior cingulum is continuous at the lingual base of the protocone on M2.

M3 (V 26241, Fig. 2e): The tooth is trapezoid in outline with the posterior border considerably shorter than the anterior border. Its general morphology is similar to that of M2, but is different in having a more buccally projected and smaller parastyle, a highly reduced metacone with a slightly convex buccal surface, a short postmetacrista posteriorly extended, and the protocone situated at the level slightly anterior to the paracone.

m1 (Fig. 2a, c): The tooth is rectangular in outline with the talonid wider and longer than the trigonid. The protolophid is notched in the middle and nearly transverse extended with the metaconid slightly posteriorly situated to the level of the protoconid. Both the protoconid and the metaconid bear a distinct rib on the anterior surface of the protoloph, and the two ribs converge at the base of the trigonid. The paralophid extends anteriorly and slightly lingually from the protoconid and then smoothly curves to the lingual side. The hypolophid lacks a notch, and is more oblique than the protolophid. The buccal wall of the hypoconid is slightly lingually slanted. The cristid obliqua extends anteriorly and slightly lingually from the hypoconid, joining the posterior wall of the protolophid at a point roughly half the height of the wall on an unworn tooth. The angle between the hypolophid and the cristid obliqua is slightly greater than 90° with meeting at a sharp angle, which should become gentler after wear as inferred from the somewhat swollen buccal side of the hypoconid. The entoconid bears a distinct anterior cristid. The anterior cingulid arises towards the lingual side and surrounds the anterobuccal corner of the crown. A weak cingulid is present at the base of the ectoflexid. The

posterior cingulid is weak without a cuspid.

m3 (Fig. 2b): Two teeth were assigned to m3. One (V 26234) is associated with dp4-m1 and partially erupted. This tooth is assigned to m3 rather than m2 because its root has not been formed, which is not consistent with the fragmentary root of m2 preserved on the mandible. Its morphology is generally similar to that of m1, but is different in being larger, and in having a more anteriorly extended paralophid, less distinct ribs on the anterior surface of the protoconid and the metaconid, a less notched protolophid (but moderately notched on V 26236), and a cristid obliqua contacting the protolophid in a relatively lower position. Further, the posterior cingulid is somewhat developed into a small cuspid representing a highly reduced hypoconulid.

dp3 (Fig. 2c): The tooth is anteroposteriorly elongated with the talonid wider than the trigonid. The trigonid is longer than wide with a well-developed paraconid, which is nearly as high as the protoconid. From the apex of the paraconid three ridges extend: two posterolingually and posterobuccally, and a short third one anteriorly. The protolophid is slightly notched, oblique, and short with the metaconid not widely separated from the protoconid. The paralophid extends anteriorly and slightly buccally from the protoconid. The hypolophid is also oblique and parallel to the protolophid, and less notched than the latter. The cristid obliqua extends anterolingually from the hypoconid, and terminates at a point slightly lingual to the protoconid in a relatively high position. A distinct ridge descends anteriorly from the entoconid to the base of the talonid, whereas a short, less distinct cristid descends from the posterior side of the metaconid. The cingulids are faint at the buccal side of the trigonid, and weak on the posterior side. (dp3: L=8.8 mm, W of Trigonid=4.0 mm, W of Talonid=4.8 mm, V 26235)

dp4 (Fig. 2a, c): The tooth is generally similar to m1 in morphology, but can be distinguished by a more distinct rib on the anterior side of the metaconid, and a more

distinct, longer cristid anterior to the entoconid. (dp4: L=9.2 mm, W of Trigonid=5.4 mm, W of Talonid=6.1 mm, V 26234)

Yimengia chaganense sp. nov.

Holotype. IVPP V 26242.1, associated left and right maxillae including P4-M3 with ectolophs partially broken off.

Locality and horizon. lower horizon of the middle part of the Arshanto Formation, Chaganboerhe.

Description. P4 (Fig. 3a): The tooth is rectangular in outline with a rounded lingual border. The parastyle is high, somewhat pillar-like, and separated from the paracone by a distinct groove on the buccal surface. The paracone bears a prominent rib on the buccal side, while the metacone is nearly flat. The parastyle, paracone, and metacone are aligned in a longitudinal line. The protocone is conical, and the protoloph extends to the anterolingual side of the paracone in a relatively high position. The metaloph (or metaconule) is lower and shorter than the protoloph, joining the ectoloph between the paracone and the metacone, but contacts the midpoint of the height rather than the apex of the protocone. Thus, the medifossette is not completely closed on the posterior side. A distinct cingulum is present and continuous on the anterior, lingual, and posterior sides, as well as the buccal side of the metacone.

M1 (Fig. 3a-b): The tooth is quadrate in outline with the width slightly greater than the length. The paracone is high and sharp with a pinched rib on the buccal side, whereas the metacone is lingually placed and tilted with a flat buccal surface. The centrocrista is straight or slightly buccally convex. The postmetacrista is moderately long and buccally deflected. The parastyle is relatively small, situated anterolingual to the paracone, and somewhat pillar-like rather than cuspate. The protocone is directly lingual to the paracone. The protoloph is arched anteriorly, smoothly joining the preparacrista. The metaloph is short, contacting the ectoloph anterior to the apex

of the metacone. The cingulum is almost continuous at the anterior, lingual, and posterior sides, but interrupted at the base of the hypocone. A distinct buccal cingulum is present at the base of the metacone.

M2 (Fig. 3a): All the second upper molars available are partially damaged. However, the morphology of M2–is similar to that of M1 as inferred from the combined preserved parts. The cingulum is nearly absent at the lingual, as well as the posterior side of the hypocone.

M3 (Fig. 3a): The tooth is roughly triangular in outline. The morphology is similar to that of M1-2, but the metacone is more reduced, more lingually placed and deflected. The metaloph is much shorter and more confluent with the centrocrista. The lingual cingulum is absent as on M2.

p3 (Fig. 3c): The tooth is rectangular in outline with the talonid slightly shorter and wider than the trigonid. The buccal paralophid descends anteriorly and slightly buccally from the protoconid to a moderately high paraconid, and then turns lingually for a short distance. The metaconid is slightly smaller than the protoconid, and closely posterolingually placed to the latter, resulting an oblique, short protolophid. The hypoconid is large, and as high as the paraconid. The cristid obliqua extends anterolingually toward the protoconid, joining the posterior wall of the protolophid in a relatively high position. The buccal wall of the cristid obliqua is somewhat lingually slanted. A very weak cingulid at the lingual base of the talonid represents a rudimentary entoconid. The buccal cingulid is faint, whereas the lingual cingulid is absent.

p4 (Fig. 3c): The tooth is similar to p3, but the metaconid is as large as the protoconid, and widely separated from the latter. The protolophid is–slightly less oblique, and the cristid obliqua joins the protoloph slightly lingual to the protoconid. A weak hypolophid extends posterolingually from the hypoconid for a short distance, and then descends to the lingual cingulid at the base of the talonid.

m1/2 (Fig. 3d-e): The tooth is rectangular with the talonid slightly wider than

the trigonid. The metaconid is slightly posterior to the protoconid, and the protolophid is nearly transversely extended with a very shallow notch in the middle. The paralophid descends from the protoconid anteriorly and slightly lingually to a low position, and then curves lingually for a short distance. The entoconid is posteriorly placed to the hypoconid. The hypolophid is complete without a notch and more oblique than the protolophid. The cristid obliqua is strong, extending from the hypoconid to the posterior wall of the protolophid at a roughly middle height, and slightly lingual to the protoconid. The anterior cingulid is continuous with the buccal cingulid of the trigonid, which is somewhat interrupted. The posterior cingulid is distinct and slightly elevated in the middle, representing a weak hypoconulid. The lingual cingulid is absent.

DP2 (Fig. 3b): The parastyle is broken off on both sides of the maxilla. The posterior part of the tooth is much wider than the anterior one. The paracone is the main cusp of the ectoloph, approximately situated in the midline with a convex buccal surface. The protoloph is mainly composed of the paraconule, from which two ridges originate: one extends buccally to the preparacrista, while the other descends posterolingually with a shallow lingual furrow and not in contact with the protocone. Thus, the protoloph forms a sharp angle. The protocone is large and posterolingually placed. The metaloph is stronger than the protoloph. A weak cingulum is discernable at the buccal base of the metacone. A tiny cuspid is present at the opening of the middle valley. Other characters are obscure due to the heavily worn state. (DP2: L=5.7 mm, W=6.0 mm, V 26242.2)

DP3 (Fig. 3b): The tooth is heavily worn, and rectangular in outline with the posterior part slightly wider than the anterior part. The parastyle is relatively small and anterior to the paracone. The paracone is sharp with a convex buccal surface, whereas the metacone is marginally more lingually placed with a slightly convex buccal surface. The postmetacrista is relatively short, and directed posteriorly and slightly buccally. The hypocone is as large as and more lingually extended than the

protocone. The protoloph and metaloph are completely separated on the lingual side. The cingula are continuous on the anterior, lingual, and posterior sides. A distinct cingulum is also present at the buccal side of the metacone. (DP3: L=7.2 mm, W=7.2 mm, V 26242.2)

DP4 (Fig. 3b): The tooth is very similar to M1-2 in morphology with equally-developed protoloph and metaloph except for a less lingually placed metacone with a shorter postmetacrista. (DP4: L=7.8 mm, W=8.3 mm, V 26242.2)

dp4 (Fig. 3e): The dp4 is similar to m1/2 in morphology, but can be distinguished by smaller size and more distinct ribs on the anterior sides of the metaconid and the protoconid. (dp4: L=7.5 mm, W of Trigonid=4.4 mm, W of Talonid=4.6 mm, V 26247.2)

Hyracodontidae Cope, 1879

Triplopus? *youjingensis* sp. nov.

Holotype. IVPP V 26248, a right mandible with p2-m3 (Fig. 5a).

Locality and horizon. Basal part of the Arshanto Formation, Nuhetingboerhe.

Description. p1: Only a single alveolus is present but, based on this, the tooth is not greatly reduced in size.

p2: The anterobuccal part of the enamel is broken off, but the dentin is still preserved. The crown is oval in outline with a main cusp (protoconid) in the middle. Two ridges descend from the protoconid anteriorly to the paraconid, and posteriorly to the hypoconid, in a relatively high position. The talonid is slightly wider and deeper than the trigonid. The entoconid is almost absent, but represented by a swelling at the posterolingual base of the talonid. The lingual cingulid is absent, and a posterior cingulid is discernable.

p3: The tooth is cracked longitudinally, and was broken off at the antero- and posterolingual parts. The trigonid is longer than wide with a relatively short protolophid. The metaconid is as large as, and slightly posteriorly situated to the

protoconid, resulting in a slightly oblique protolophid. The paralophid descends anteriorly from the protoconid to a relatively high paraconid, and then extends lingually for an unknown distance due to breakage. The cristid obliqua ascends anteriorly from the prominent hypoconid to the protoconid in a high position. The posterolingual part of the talonid is swollen, indicating an incipient entoconid, and a low, weak ridge between the entoconid and hypoconid represents a rudimentary hypolophid. The cingulid is absent on the lingual and buccal sides.

p4: The tooth is roughly rectangular in outline with the talonid shorter and slightly wider than the trigonid. The metaconid is as large as the protoconid, and slightly posteriorly placed to the latter. Thus, the protolophid is nearly transverse. The paralophid descends anteriorly and slightly lingually from the protoconid to a relatively high paraconid, and then descends lingually to the base of the trigonid. The hypoconid is strong and high, and the cristid obliqua slightly rises to the lingual part of the protoconid in a high position. Although the entoconid is broken off, there is a transversely extended, faint swollen low ridge between the hypoconid and presumably present entoconid, foreshadowing the incipient hypolophid as in p3. Weak cingulids are present at the base of the trigonid and the buccal side of the talonid.

m1-2: The m1 is heavily worn and the m2 is moderately worn. The tooth is rectangular in outline with the talonid slightly longer than the trigonid. The protolophid is slightly oblique and parallel to the hypolophid. The paralophid extends anterolingually from the protoconid to the paraconid, and then extends lingually for a short distance. The cristid obliqua extends anteriorly to the protolophid in a high position slightly lingual to the protoconid. The anterior cingulum is relatively distinct, slightly wrapping the trigonid at the lingual and buccal base, but does not enclose the trigonid. A weak cingulid is present at the base of the ectoflexid.

m3: The tooth is similar to m1-2, but is relatively longer and narrower. The paralophid is more anteriorly extended, and descends to a relatively high paraconid. A relatively long cristid obliqua extends anteriorly from the hypoconid and then turns somewhat lingually at its anterior end, joining the posterior wall of the protolophid in a relatively high position, which is higher than the middle height of the protolophid. The posterior cingulid is partially broken off, but the hypoconulid was apparently highly reduced and cingulid-like.

The preserved horizontal ramus is relatively shallow with a slightly convex ventral border. Three mental foramina are present below the postcanine diastema, p1, and p2, respectively. The posterior border of the symphyseal region is at the level of the anterior border of p1.

Forstercooperiidae Kretzoi, 1940

Gobioceras wangi gen. et sp. nov.

Holotype. IVPP V 26249, a right mandible with m1-m3.

Locality and horizon. Basal part of the Arshanto Formation, Nuhetingboerhe.

Description. M3 (Fig. 5c): The tooth is roughly triangular in outline. The paracone is vertically placed, bearing a strong paracone rib on the buccal side that is slightly posteriorly directed. The metacone is considerably lingually placed, nearly flat, and reduced to a short ridge with a shallow notch in the middle. The parastyle is strong, more buccally projected than the paracone, and is separated from the paracone and somewhat compressed. The protocone is directly lingual to the paracone. The protoloph is long and joins the ectoloph between the preparacrista and the parastyle. The hypocone is as lingually placed as the protocone, but smaller. The metaloph is very short and nearly confluent with the centrocrista, resulting in a triangular outline of the crown. The anterior cingulum is much stronger than the posterior one, and a feeble lingual cingulum is present at the base of the middle valley. (M3: L=21.1 mm, W=24.0 mm, V 26250.1).

Only a fragmentary ectoloph of M2 is preserved (Fig. 5d), and probably from the same individual as the M3. The metacone is nearly flat and moderately lingually placed, bearing an indistinct metacone rib. Although the parastyle is partially broken, it is likely as strong as that of M3.

A right mandible is preserved with m1-3 and the alveoli of p1-4 (Fig. 5b).

m1-2 (Fig. 5b): The m1 and m2 are similar in morphology, with the talonid wider and longer than the trigonid. The trigonid is obliquely U-shaped with the protolophid posterolingually extended and parallel to the relatively long anterior branch of the paralophid. The buccal branch of the paralophid is perpendicular to its anterior branch anteriorly and the protolophid posteriorly. The hypolophid is also oblique and parallel to the protolophid, and smoothly rather than sharply joints the cristid obliqua at the hypoconid. The cristid obliqua is relatively short, contacting the protolophid slightly lingual to the protoconid in a relatively high position (above the half height of the protolophid). Anterior to both the metaconid and entoconid, there is a weak ridge. A weak cingulid is present on the buccal side and continuous to the posterior side. The lingual cingulid is absent. Another specimen (IVPP V 26251) shows some variation in that the size is slightly smaller, and the buccal cingulid is only present at the base of the ectoflexid.

m3 (Fig. 5b): The entoconid of m3 is broken. The tooth is generally similar to the preceding molars. It mainly differs from m1-2 in having a relatively longer trigonid, a cristid obliqua anteriorly extended, and a nearly absent buccal cingulid. Furthermore, a small cuspid is present approximately at the midline of the anterior wall of the protolophid.

Hyracodontidae Cope, 1879

Ephyrachyus Wood, 1934

Remarks

Wood[7] thoroughly reviewed hyrachyids from North America, a group consisting of

four genera and 12 species. Radinsky[8], however, considered only one genus, *Hyrachyus*, and three species to be valid, including *H. modestus* and *H. eximius* from North America and *H. minimus* from Europe. European '*Chasmotherium*' *stehlini* was also considered a synonym of *H. modestus*[8]. Radinsky[9] further reassigned some species of hyrachyids to *Triplopus* or *Forstercooperia*. As pointed out by Schoch[10], Wood's revision may have been 'oversplit', whereas Radinsky's revision probably represented an extreme taxonomic "lumping". Four genera of hyrachyids have been reported from Asia, including *Hyrachyus*[11], *Subhyrachyus*[12], *Aktauhyus*[13,14], and probably *Jhagirilophus*[15]. However, Lucas et al.[16] treated *Subhyrachyus* as a synonym of the lophialetid *Eoletes*. Four species of *Hyrachyus* have been reported from Asia, consisting of *H. asiaticus*[17], *H. metalophus*[18], *H. modestus*[18,19], and *H. tongi*[11]. '*Hyrachyus*' *minor*, as well as '*H.*' *lunanensis*, from the Lumeiyi Formation of the Lunan Basin, Yunnan Province could be included in *Aktauhyus*[13].

Ephyrachyus woodi sp. nov.

Holotype. IVPP V 26252, a right maxilla with P2-M3, right and left p2-3, and fragments p4 and lower molars.

Locality and horizon. lower horizon of the upper part of the Arshanto Formation, Chaganboerhe

Description. P2 (Fig. 6a): The tooth is roughly quadrilateral in outline with a rounded lingual border. The paracone and the metacone are rudimentary and separated with the metacone slightly smaller and lower than the paracone. The paracone and the metacone merged with a sharp ectoloph, extending a longer ridge anteriorly from the paracone and a short ridge posteriorly from the metacone. The buccal surface of the paracone and the metacone are slightly convex with a weaker metacone rib. The parastyle is low and rather weak. The protocone is large and conical and is directly lingual to the paracone. The protoloph extends anterobuccally

from the protocone but dies out before reaching the anterolingual base of the paracone. The metaloph, mainly composed of the metaconule, is lower and shorter than the protoloph, joining to the anterolingual base of the metacone and separated from the protocone by a moderately deep valley. A weak cingulum continues around the posterior and lingual bases of the crown, but is absent at the base of the protocone. A weak cingulum is present buccal to the metacone.

P3 (Fig. 6a): The tooth is quadrilateral in outline with the width greater than the length. The paracone and the metacone are closely placed, equal in size, and merged into the ectoloph. The buccal surfaces of the paracone and the metacone bear prominent ribs and are divided by a narrow groove from each other. A crista is present on the lingual surface of the paracone. Both the parastyle and the metastyle are distinct. However, the former is larger and slightly lower than the latter. The protocone is directly lingual to the paracone, extending the protoloph anterobuccally to join the preparacrista near the parastyle in a high position. Another ridge (endoprotocrista) extends posterobuccally from the protocone with a relatively sharp angle, bypassing the metaconule. The metaloph incorporates the metaconule and is transversely extended and sharp, joining the ectoloph and endoprotocrista in relatively high positions. A weak cingulum is continuous on the anterior, lingual, and posterior sides, and is discernable buccal to the metacone.

P4 (Fig. 6a): The tooth is similar to P3 in morphology except that the paracone and metacone are widely separated with the metacone slightly more lingually placed. The paracone and metacone ribs are divided by a wide, triangular trough, and that the parastyle is larger and relatively higher.

M1-2 (Fig. 6a): The M1 and M2 are similar in morphology, although the parastyle and hypocone of M1 are broken off. The tooth is nearly quadrate in outline with relatively high, sharp lophs, which are partially related to its lightly worn condition. The paracone is high and pinched on the buccal side, with a nearly vertical, weak crista on the lingual side. The metacone is slightly lower than the

paracone, strongly lingually depressed with a distinct rib on the buccal surface, and bears a relatively long postmetacrista. The parastyle is large, expanded, and situated anterior to the paracone. The protocone is roughly lingual to the paracone, extending the slightly anterior-arched protoloph toward the parastyle and then joining the preparacrista. The hypocone is as large as the protocone, and the metaloph is straighter and shorter than the protoloph, almost joining the top of the ectoloph close to (on M1) or slightly anterior to (on M2) the apex of the metacone. A thin but distinct cingulum is continuous on the anterior, lingual, and posterior sides. A weak cingulum is discernable on the buccal side of the metacone and between the paracone and the parastyle.

M3 (Fig. 6a): The tooth is trapezoid in outline with a truncated posterobuccal part. The metacone is much reduced with a short postmetacrista, which is slightly buccally flared. The metacone rib is absent on the buccal surface. The parastyle is relatively more buccally placed than that of M2. The metaloph is slightly anteriorly arched and joins the top of the metacone. The cingulum is partially broken off or imbedded in the socket, but probably similar to that of M2.

The fragmentary symphyseal region of the mandible preserves the roots of three lower incisors and a slightly larger canine, which are closely placed to each other (Fig. 6b). The cross sections of the incisor roots are rounded, but that of the canine is laterally compressed oval. Based on the alveoli of anterior teeth, it is reasonable to infer that i2 is larger than i1, which is in turn slightly larger than i3.

p1: This tooth only preserves the single root, which is oval in cross section (Fig. 6c).

p2 (Fig. 6c-d): The tooth is elongated anteroposteriorly with a main protoconid placed in the middle. The paracristid extends anteriorly and slightly buccally from the protoconid, and then curves lingually in a low position. Two ridges descend posteriorly from the protoconid: the buccal one is indistinct and joins the cristid obliqua, whereas the lingual one is more prominent. The hypoconid is large but

lower than the protoconid, extending the cristid obliqua anteriorly with a vertical buccal surface and a sloped lingual surface. The entoconid is absent. A weak cingulid is discernable at the lingual base of the talonid. (p2: L=8.3 mm, W≈3.7 mm, V 26252)

p3 (Fig. 6c-d): The tooth is elongated anteroposteriorly with the talonid wider and as long as the trigonid. The trigonid is longer than wide, whereas the width of the talonid is slightly greater than the length. The paracristid descends anteriorly and slightly buccally from the protoconid, and then curves lingually to a tiny paraconid, which forms a small hook from the rest of the paracristid. The metaconid is as large as the protoconid, and situated posterolingual to the latter. The protolophid is short and divided by a shallow notch. The cristid obliqua extends anteriorly and slightly lingually from the hypoconid to the posterior side of the protoconid at mid-height. The buccal surface of the cristid obliqua is vertical as in p2, whereas the lingual side forms a sloped surface with a faint rib extending lingually from the hypoconid. A weak ridge descends from the hypoconid along the posterior border of the talonid, and joins the lingual cingulid at the base of the talonid. A very small cuspid arises from the lingual cingulid foreshadowing the development of the entoconid. A weak cingulid is also present at the base of the ectoflexid. (p3: L=11.0 mm, W=6.1 mm, V 26252)

Only a fragmentary p4 is preserved (Fig. 6e). The cristid obliqua extends from the hypoconid to the lingual side of the protoconid in a high position. The buccal cingulid is distinct and likely continuous.

Only fragmentary lower molars are preserved (Fig. 6f). However, it is reasonable to infer that the metaconid is posterolingually placed to the protoconid, resulting in an oblique protolophid, the cristid obliqua descends anteriorly from the hypoconid to the middle height of the protolophid, and that the buccal cingulid is present and probably continuous.

Comments on *Ephyrachyus implicatus* (=*Triplopus implicatus*)

Radinsky[9] assigned the following specimens to '*Triplopus*' *implicatus*: AMNH FM 2341, 2342 (holotype of '*Eotrigonias*' *petersoni*), 5074, 5075, 5078 (holotype of '*Ephyrachyus*' *implicatus*), 13194, and 13202; CM 9384, 18461-18464; UCMP 69374-69376.

After checking other all available specimens and casts (except for casts of CM 18416-18464) housed at the AMNH that were assigned to '*Triplopus implicatus*' by Radinsky[9], following brief comments need to be addressed: 1) the lower check teeth of AMNH FM 5078, 5074, and CM 9384 are similar in size and morphology, and thus could be assigned to the species *Ephyrachyus implicatus*; 2) the M3 and p4-m2 of UCMP 69374-39376 are very similar to those of AMNH FM 2341, 2342, and 13202 except for a more distinct entoconid on p4, and they probably belong to *Triplopus petersoni*; 3) AMNH FM 13194 from the mid-late Uintan may represent a different species of *Triplopus*, characterized by a distinct entoconid on p4, short and anterolingually directed paralophids on p4-m3, and oblique hypolophids on m1-2.

Hyrachyidae Osborn, 1892

Hyrachyus? *tumidus* sp. nov.

Holotype. IVPP V 26253.1, a right maxilla with broken P3-M2.

Locality and horizon. Basal part of the Arshanto Formation, Nuhetingboerhe.

Description. A right maxilla preserves nearly complete P3, incomplete P4-M2, and roots of P1-2. The P1 bears two roots, and P2 has a triangular outline, suggesting a well developed protocone at the posterolingual side.

P3 (Fig. 6g): The parastyle, metacone and metaconule are partially broken. The tooth is rectangular in outline with the width greater than the length. The paracone and the metacone are closely placed, separated by a narrow groove on the buccal side. The buccal surfaces of the paracone and metacone are rounded and swollen rather than rib-like with the former slightly more convex. The parastyle is broken off.

The protoloph joins the ectoloph in a relatively high position and swings posteriorly passing the metaconule. Although the metaconule is nearly completely damaged, its low lingual end dies out before reaching the protoloph, resulting in a posterior outlet forthe median valley. The cingulum is distinct and continuous around the anterior, lingual, and posterior sides, although the lingual cingulum is partially broken. Buccal to the metacone, there is also a weak cingulum at the base.

P4 (Fig. 6g): The lingual half of the tooth is broken off, and the buccal surface of the paracone is damaged. The paracone and metacone are more separated than those on P3. A crista is present at the base of the lingual surface of the paracone. The parastyle is somewhat pinched, relatively high, and anterobuccal to the paracone. The protoloph connects to the ectoloph in a high position as in P3. The metaconule joins the ectoloph anterior to the metacone and slightly higher than the corresponding joint of the protoloph.

M1-2 (Fig. 6g): Both M1 and M2 are partially broken off. The following description combines their morphologies, assuming that they have similar morphologies to other *Hyrachyus*. The outline of the crown is roughly quadrate. The paracone is high and sharp with a strong rib on the buccal side. The metacone is lingually placed, slightly concave with a moderately long postmetacrista that is slightly buccally deflected. The metacone rib is distinct, but is weaker on M2 than that of M1. The parastyle is large (relatively larger on M2), fan-shaped, but is slightly separated from the paracone rather than closely appressed. The protoloph is considerably longer than the metaloph, which joins the ectoloph slightly anterior to the metacone. The posterior cingulum is distinct, but absent at the posterior and lingual sides of the hypocone. A weak buccal cingulum is present at the base of the metacone. The anterior and lingual cingula are unknown due to damage.

m1-2 (Fig. 6h-i): Only the trigonids of m1 and m2 are preserved, and they are probably from the same individual as the maxilla based on a similar degree of wear. The trigonid is roughly U-shaped with the transversely extended protolophid parallel

to the anterior branch of the paralophid. The buccal branch of the paralophid is anteriorly and slightly lingually oriented from the protoconid. The anterior branch of the paralophid is relatively long. The metaconid is somewhat conical and higher than the protoconid on the slightly worn m2. Based on the fragmentary cristid obliqua preserved on m1, it extends to the protolophid lingual to the protoconid, and joins the protolophid in a relatively low position. A weak cingulid is preserved at the anterobuccal corner and continueson the anterior side.

Comments on other species of *Hyrachyus* from China

Hyrachyus neimongoliensis from the Arshanto Formation in the Erlian Basin[20] is known from a fragmentary skull with P3-M3 (IVPP V 5721), and Huang and Wang[11] have argued its probable affinity with amynodontids. Although Qi[20] assigned it to *Hyrachyus*, he also noticed that its cranial morphology and size resembles those of *Pappaceras confluens* (=*Forstercooperia huhebulakensis*)[21,22]; furthermore, a relatively long distance between the postglenoid process and the occipital condyle (about 20% of the skull length) and a possible shallow preorbital fossa according to the figure (Qi[20], fig. 29) suggest that IVPP V 5721 is more similar to *Pappaceras* than to *Hyrachyus*[21]. IVPP V 5721 is also different from *Hyrachyus* but similar to *Pappaceras* in the presence of the antecrochet on M1 (as in *P. confluens* and *P. meiomenus*), flat and straight metacones on M1-2, and relatively pinched parastyles on M2-3 (Qi[20], fig. 29). The length of the molar series (about 77.0 mm) and less reduced M3 metacone of IVPP V 5721 suggest that it is more similar to *P. minutus* or *P. meiomenus* than to *P. confluens* [21,23,24]. As a result, without access to IVPP V 5721, we consider '*Hyrachyus neimongoliensis*' likely to be a synonym of *Pappaceras minutus* or *P. meiomenus*. If the latter case is true, the specific name *P. neimongoliensis*[20] has priority over *P. meiomenus*[24].

Qi[20] aslo assigned a left M3 (IVPP V 5728) to *Hyrachyus* cf. *H. eximus* from the Arshanto Formation at Huheboerhe. But the M3 has an inverted U-shaped ridge

composed of the protoloph, ectoloph, and metaloph, and a highly reduced metacone situated in the midway of the metaloph, suggesting that it is more probably assigned to depertellid *Irenolophus*[25].

Upper molars of *Hyrachyus tongi* from the Hedi Formation in the Yuanqu Basin[11] are more similar to those of *Triplopus* than to *Hyrachyus* in having reduced, pillar-like parastyles, concave metacone without metacone ribs, protocone situated directly lingual to the paracone, an antecrochet on M1, and metacone of M3 very short, more lingually depressed and lingually directed. Furthermore, among *Triplopus*, '*Hyrachyus*' *tongi* most resembles *T. rhinocerinus* in having molarized P2, incipient separation between the protocone and hypocone on P3 and P4 (more distinct on P4), and the metaconule of P3-4 contacting the protoloph and enclosing the medifossette[9,26]. However, '*Hyrachyus*' *tongi* can be mainly distinguished from *T. rhinocerinus* by a slightly less molarized P2 and the junction between the metaconule and the crista on P3-4, resulting a small pit [11]. As a result, we consider '*Hyrachyus*' *tongi* to be more reasonably assigned to *Triplopus tongi*.

Chow and Qi[18] named *Hyrachyus metalophus* from the Guanzhuang Formation in Xintai, Shandong Province. *Hyrachyus metalophus* is mainly different from *Ephyrachyus woodi* in its larger size, prominent metacone ribs and smaller parastyles on M1-3, cristid obliqua of p3-4 oriented to the midpoint of the protolophid, and cristid obliqua of lower molars more developed. However, the more lingually directed cristid obliqua from p2 to p4, as a diagnosis character of *H. metalophus*, is also seen in *H. eximius* (AMNH FM 1645)[7]. Chow and Qi[18] also tentatively assigned several maxilla and mandibles (IVPP V 6391, 6392) from the same locality to *Hyrachyus modestus*?. However, the U-shaped ridge formed by the protoloph, ectoloph, and metaloph on M2 with a relatively reduced metacone, highly reduced paralophs and cristid obliquae on m1-3, and the tops of the protolophs and hypolophs posteriorly curved on m3 suggest that these specimens more likely belong to deperetellid tapiroids[2].

Common synapomorphic characters of some clades

The Tapiridae-Deperetellidae clade (Fig. 7, node G) in the most parsimous tree is supported by following common synapomorphic characters (Supplementary Table 1): P3 postprotocrista absent (49:0, character and state), P3 metaloph joining the ectoloph in a high position and slightly anterior to the apex of the metacone (51:2), P3 semimolariform without the lingual ridge or molariform (53:3, 5), P4 postprotocrista absent (75:0), P4 semimolariform without the lingual ridge or molariform (80:3, 5), M1-2 metacone strongly lingually displaced compared to the paracone (103:2), p4 trigonid shorter than the talonid (196:0), p4 crisitid obliqua high and composed of two ridges (208:2), the orientation of basioccipital nearly horizontal (286:1), and the height of the mandibular corpus constant (349:0).

The Paraceratheriidae-Rhinocerotidae clade (Fig. 7, node S) in the most parsimonious tree is supported by the following common synapomorphic characters (Supplementary Table 1): the buccal surface of M1-2 paracone broadly convex (98:0), the postcanine diastema length 50–100% of m1 length (149:1), and position of metaconid slightly more posteriorly displaced related to the protoconid on p3 (183:1).

The clade (Fig. 7, node P), comprising Amynodontidae and the *Eggysodon*-Paraceratheriidae-Rhinocerotidaeis clade, in the most parsimonious tree is supported by the following common synapomorphic characters (Supplementary Table 1): P4 metaloph posterolingually extended (83:2), M1-2 paracone somewhat lingually slanted (99:1), p3 hypolophid lophid and distinctly lower than protocristid (190:3), p4 anterior branch of the paracristid shorter than the buccal branch (200:0), p4 hypolophid either weak, curving along the posterior border or lophid and distinctly lower than protocristid (212:2, 3), m1-2 lingual cingulid incomplete (215:1), and m3 lingual cingulid present but incomplete (246:1) (Supplementary Table 1).

Supplementary Note 2.

Character list

Most characters and states are new, and some are derived or modified from previous researchers, including H89: Hooker[6]; H94: Hooker[27]; BB95: de Bonis et al.[28]; DH97: Dashzeveg and Hooker[5]; H01: Holbrook[29]; A02: Antoine[30]; F02: Froehlich[31]; HD03: Hooker and Dashzeveg[32]; HD04: Hooker and Dashzeveg[33]; C05: Colbert[34]; AA07: Anquetin et al.[35]; M08: Mihlbachler[36]; H09: Holbrook[37]; B10: Bai et al.[38]; H10: Hooker[39]; CD12: Chen et al.[40]; KL12: Kondrashov and Lucas[41]; DR13: Danilo et al.[42]; RH14: Rose et al.[43]; RR15: Robinet et al.[44]; and W16: Wang et al.[24].

Dental characters

Upper Dentitions

Upper Incisors

1. Number of upper incisors: (0) 3; (1) 1 (I1).
2. Upper incisor relative sizes: (0) approximately equal in size; (1) enlarged from I3 to I1; (2) gradually increased from I1 to I3; (3) I3 considerably larger than I1 and I2, (4) I2 the largest.
3. Incisors shape: (0) spatulate; (1) I3 pointed, I1-2 spatulate; (2) all pointed; (3) I1 chisel-like, I2-I3 reduced or absent; (4) I1 hypertrophied and conical, I2-3 reduced or absent.
4. Arrangement of upper incisors: (0) loosely arranged and evenly distributed, (1) closely arranged.
5. Upper incisors: (0) arched, (1) converging anteriorly and forming an acute angle, nearly anteroposteriorly displaced.

Upper Canine

6. Upper canine size: (0) medium, larger than the incisors, (1) small, comparable to or even smaller than incisiors, (2) large, considerably larger than incisors, (3)

hypertrophied, (4) absent.

Most amynodontids have hypertrophied upper canines (state 3), while *Heptodon*, *Pappaceras*, *Forstcooperia*, and a few amynodontids have large upper canines (state 2), which are relatively smaller than the size of hypertrophy.

7. Postcanine diastema: (0) short, (1) long, (2) absent. (RH14:105; H09:28)

As suggested by Holbrook (2009), if the postcanine diastema is not much longer than P1, it is coded as state 0 (short).

P1

8. P1: (0) present; (1) absent. (DR13:8; F02:20; H89:11; HD04:20; RR15:1)
9. Post P1 diastema: (0) present; (1) absent. (modified from H10:44; HD04:52)
10. P1 root: (0) single; (1) double; (2) three.
11. P1 elongation: (0) shorter than P2; (1) as long as or longer than P2. (DR13:9)
12. P1 posterolingual cusp (protocone or hypocone): (0) absent; (1) present. (C05:28; DH97:17)

Dashzeveg and Hooker (1997) referred to a large posterolingual cusp of P1 as a hypocone, however, its homology with the protocone could not be excluded. For convenience, we use posterolingual cusp instead of hypocone.

13. P1 metacone: (0) absent; (1) present.
14. P1 protoloph: (0) absent; (1) rudimentary and cingulum-like; (2) prominent and loph-like.
15. P1 metaloph (or metaconule): (0) absent, (1) present.

P2

16. P2: (0) longer than wide; (1) wider than long. (AA07:37; CD12:42)
17. P2 occlusal outline: (0) oval; (1) triangular, tapering forward; (2) subtriangular, anterior side slightly slanted; (3) rectangular. (modified from DR13:11)
18. P2 metacone: (0) absent or indistinct; (1) present, small, close to paracone; (2)

present, as large as and appressed to the paracone; (3) present, about as large as and separate from the paracone. (modified from H09:30; RH14:111)

19. P2 metacone position relative to the paracone: (0) posteriorly; (1) posterolingually.
20. Buccal surface of P2 paracone: (0) nearly flat; (1) conical; (2) broadly swollen; (3) rib-like.
21. Buccal surface of P2 metacone: (0) nearly flat; (1) conical; (2) broadly swollen.
22. P2 parastyle size: (0) absent; (1) less than the half of the paracone; (1) at least equal to the half of the paracone.
23. P2 metastyle: (0) absent; (1) present.
24. P2 protocone: (0) weak or absent; (1) prominent. (B10:3; H10:54)
25. Position of P2 protocone: (0) posterior, (1) on transverse axis, (2) anterior. (modified from B10:4)
26. P2 protoloph (or protoconule): (0) absent, (1) weak and low, (2) distinct and low, (3) prominent and high.

When the protoloph is cingulum-like as in *Protorohippus*, the protoloph was considered as being weak and low (state 1). When the protoloph is ridge-like but still in a relatively low position as in *Homogalax*, it is coded as being distinct and low (state 2).

27. Orientation of P2 protoloph (or the preparaconule crista) on the buccal side: (0) toward the lingual base of the paracone, (1) toward the anterolingual side of the paracone, (2) toward the preparacrista, (3) toward parastyle.
28. P2 postprotocrista: (0) absent, (1) present.
29. P2 hypocone: (0) absent; (1) present.
30. P2 protocone and hypocone: (0) fused, (1) incipient and separated from the protocone, (2) moderately separated, (3) completely separated (modified from A02:94)

As in *Teletaceras*, the protoloph extends posteriorly from the protocone with the

hypocone completely fused (state 0). In some taxa as *Colodon*, the protocone and hypocone are closely appressed and separated by a lingual groove (state 1). In *Juxia*, the protocone and hypocone are distinctly separated, but the middle valley is still closed (state 2). In *Trigonias* and *Subhyracodon*, the protocone and hypocone are completely separated and the middle valley is open (state 3).

31. P2 hypocone position related to the protocone: (0) posteriorly, (2) posterolingually, (1) posterobuccally.
32. P2 metaloph (or metaconule): (0) absent; (1) present, extending toward protocone, (2) present, extending toward hypocone (or posterior end of protocone), (3) much shorter and separated from the protoloph.
33. P2 metaloph (or metacounule) buccal extension: (0) low; (1) high.

P3

34. P3 lingual cingulum: (0) complete; (1) broken or absent. (C05:30)
35. Position of parastyle related to the paracone of P3: (0) anteriorly; (1) slightly anterobuccally; (2) strongly anterobuccally; (3) anterolingually.
36. P3 paracone and metacone: (0) well separate, (1) close together, (2) very close. (modified from HD04:51; B10:7)
37. P3 metacone position relative to the paracone: (0) posteriorly, (1) posterolingually. (RH14:132)
38. Buccal surface of P3 paracone: (0) nearly flat; (1) convex; (2) rib-like; (3) slightly convex.
39. Buccal surface of P3 metacone: (0) nearly flat; (1) convex; (2) rib-like; (3) slightly convex.
40. P3 parastyle: (0) less than the half of the paracone; (1) at least equal to the half of the paracone. (modified from RR15:19)
41. P3 metastyle: (0) absent or weak; (1) present.
42. Orientation of P3 postmetacrista: (0) posteriorly; (1) slightly posterobuccally;

(2) considerably posterobuccally.

43. P3 crista: (0) absent, (1) present.

44. Position of P3 protocone: (0) posterior, (1) on transverse axis, (2) anterior.

45. P3 paraconule: (0) present in the middle of the protoloph; (1) present and lingually positioned; (2) absent or indistinct. (modified from RH14:116)

In some advanced ceratomorphs (e.g., *Triplopus*, *Prohyracodon*, *Juxia*) the paraconules are merged with the protolophs, and are not demarcated from the latter (state 2).

46. Connection between protocone and paraconule: (0) notched; (1) loph or slightly notched; (2) completely separated.

In *Deperetella* the paraconule is completely separated from the protocone (state 2).

47. Orientation of P3 protoloph (or the preparaconule crista) on the buccal side: (0) toward the lingual or anterolingual base of the paracone; (1) toward parastyle; (2) toward the preparacrista and parastyle junction; (3) midway of preparacrista; (4) toward paracone

48. P3 endoprotocrista: (0) absent; (1) present.

49. P3 postprotocrista: (0) absent, (1) present.

50. P3 metaconule: (0) present (1) absent or indistinct. (modified from RH14:119)

In some taxa (e.g., *Triplopus*, *Prohyracodon*, *Juxia*) the metaconules are merged with the metalophs, and are not demarcated from the latter (state 1).

51. P3 metaloph (or premetaconule crista) joins the ectoloph: (0) low, at the base of the anterolingual side of the metacone; (1) low, at the base of lingual side of the metacone; (2) high, slightly anterior to the apex of the metacone;

52. P3 hypocone: (0) absent; (1) present.

In *Metamynodon*, the metaconules is elongated and forms the posterior transverse loph, which is partially or completely separated from the protoloph. However, the hypocone is still absent on P3 of *Metamynodon* (state 0).

53. Degree of P3 molarization: (0) unmolariform; (1) premolariform; (2) submolariform; (3) semimolariform without the lingual ridge; (4) semimolariform with the lingual ridge; (5) molariform. (modified from A02:102)

54. P3 hypocone position related to the protocone: (0) posteriorly; (1) posterolingually; (2) posterobuccally.

55. P3 metaloph (or metaconules) orientation when a hypocone present: (0) hypocone; (1) protocone.

This character is somewhat similar to the character 19 of Dashzeveg and Hooker (1997), and the character state 1 was used as a synapomorphy of *Desmatotherium* and *Dilophodon*.

56. P3 metaloph (or metaconules) orientation: (0) nearly transversely extended; (1) posterolingually extended; (2) anterolingually extended

P4

57. P4 occlusal outline: (0) triangular, (1) subquadrangular, (2) square or rectangular. (DR13:20)

58. P4 lingual cingulum: (0) with reduced on the protocone or absent; (1) present and continuous. (modified from DR13:21).

59. Orientation of P4 preparacrista: (0) anteriorly; (1) slightly anterobuccally; (2) strongly anterobuccally; (3) anterolingually extended.

60. P4 paracone and metacone: (0) closely placed; (1) moderately separated; (2) widely separated.

61. P4 metacone situated relative to the protocone: (0) posteriorly; (1) slightly posterolingually; (2) strongly posterolingually. (RH14:132, with additional character state)

In *Schlosseria* and *Lophialetes*, the metacone is strongly lingually displaced related to the paracone (state 2).

62. The buccal surface of P4 paracone: (0) convex; (1) rib-like; (2) slightly convex.

63. The buccal surface of P4 metacone: (0) nearly flat; (1) convex; (2) rib-like; (3) slightly convex.

64. P4 paracone: (0) vertically implanted; (1) slightly lingually inclined; (2) considerably lingually inclined.

65. P4 parastyle: (0) less than the half of the paracone; (1) at least equal to the half of the paracone. (modified from RR15:19)

66. P4 metastyle: (0) absent or indistinct; (1) present.

67. Orientation of P4 postmetacrista: (0) posteriorly; (1) posterobuccally.

68. P4 crista: (0) absent, (1) present.

69. Position of P4 protocone: (0) on transverse axis; (1) anterior; (2) posterior. (modified from B10:4)

70. P4 protocone: (0) vertically implanted, (1) slightly buccally slanted; (2) considerably buccally slanted.

71. P4 paraconule: (0) large and distinct, (1) small, (2) absent. (modified from RH14:124; KL12:7, in part)

72. Connection between protocone and paraconule: (0) loph or slightly notched; (1) notched; (2) completely separated

73. Orientation of P4 protoloph (or the preparaconule crista) on the buccal side: (0) toward the lingual or anterolingual base of the paracone; (1) toward parastyle; (2) toward the preparacrista and parastyle junction; (3) midway of preparacrista; (4) toward paracone.

74. P4 endoprotocrista: (0) absent; (1) present.

75. P4 postprotocrista: (0) absent; (1) present.

76. P4 metaconule: (0) similar in size to paraconule; (1) present, small; (2) absent or indistinct.

77. P4 metaloph (or metaconule) joins the ectoloph: (0) low, at the base of lingual side of the metacone; (1) low, at the base of the anterolingual side of the metacone; (2) high, nearly at the apex of the metacone; (3) high, towards the

middle of the centrocrista.

78. P4 endometacrista: (0) absent; (1) present. (RR15:32)

In *Homogalax* and *Isectolophus*, a ridge on the lingual surface of the metacone connects the apex of the metacone and metaloph, which is displaced at the base of the metacone (state 1).

79. P4 hypocone: (0) absent; (1) present. (RH14:131, in part)
80. Degree of P4 molarization: (0) unmolariform; (1) premolariform; (2) submolariform; (3) semimolariform without the lingual ridge; (4) semimolariform with the lingual ridge; (5) molariform.
81. P4 hypocone position related to the protocone: (0) posterior; (2) posterolingually; (1) posterobuccally.
82. P4 metaloph (or metaconules) orientation when a hypocone present: (0) hypocone; (1) protocone.
83. P4 metaloph (or metaconules) orientation: (0) anterolingually extended; (1) nearly transversely extended; (2) posterolingually extended. (similar to A02:103)

M1-2

84. Upper molar buccal and lingual cusp outer walls converge at: (0) between 90° and 100°, (1) at less than 90°. (modified from H94:12; F02:65)
85. Upper molar (M1-2) ectocingulum: (0) present and continuous; (1) interrupted at the paracone, forming a ridge and marginal ajacent to the metacone; (2) present adjacent to metacone, bulbous and encroaching on stylar shelf; (3) absent or very weak. (modified from DH97:12, C05:24, RH14:135)
86. Upper molar lingual cingulum: (0) relatively complete (1) interrupt at the protocone; (2) interrupt at the hypocone, (3) restricted to the medivallum, (4) absent. (modified from C05:23)

87. Upper molar lingual margins of paracone and metacone: (0) rounded; (1) nearly flat; (2) with a crista. (M08:62, in part)

88. M1 length and width ratios: (0) wider than long; (1) roughly as long as wide; (2) longer than wide.

89. M2 length and width ratios: (0) wider than long; (1) roughly as long as wide; (2) longer than wide.

90. Orientation of M1-2 preparacrista: (0) anteriorly; (1) anterobuccally; (2) anterolingually.

91. M1-2 centrocrista: (0) present, straight, (2) present, slightly flexed buccally. (modified from H94:10, HD03:10, HD04:10, H09:36, H10:9, B10:25, RH14:137,)

92. Those with straight centrocrista: (0) contacting the posterolingual side of the paracone; (1) contacting the posterior side of the paracone, thus forming a U-shaped ectoloph.

In *Colodon occidentalis* and deperetellids, the centrocrista contacts the posterior side of the paracone instead of posterolingual side of the paracone, and marked by a shallow notch; thus, the ectoloph is inverted U-shaped (state 1).

93. M1-2 parastyle morphology: (0) small and low; (1) moderate to large fan-shaped; (2) pillar or rib-like. (modified from H09:42, RH14:150)

94. Those with pillar or rib-like parastyle with groove separating the paracone (parastyle fold): (0) narrow, (1) wide and open.

95. M1-2 parastyle: (0) separated from the paracone; (1) closely compressed to the paracone. (modified from C05:11, DH97:7)

96. M1 parastyle position related to the paracone: (0) anterior; (1) anterobuccal; (2) anterolingual.

97. M2 parastyle position related to the paracone: (0) anterior; (1) anterobuccal; (2) anterolingual.

98. The buccal surface of M1-2 paracone: (0) broadly convex; (1) pinched.

99. M1-2 paracone: (0) vertically implanted; (1) somewhat lingually slanted, (2) distinctly lingually slanted.

100. Upper molar mesostyle: (0) absent; (1) variably developed by forming a ridge on the buccal wall between paracone and metacone; (2) small cingulum-like (modified from H94:11)

101. Buccal surface of M1-2 metacone: (0) nearly flat; (1) convex; (2) rib-like; (3) slightly convex (C05:8; similar to RH14:141, but with different states)

102. M1-2 metacone: (0) vertically implanted; (1) tilted lingually slightly; (2) tilted lingually strongly. (modified from DH97:5, HD04:44, B10:23)

103. M1-2 metacone position compared with paracone: (0) slightly lingually displaced; (1) distinctly lingually displaced, (2) strongly lingually displaced; (3) posteriorly displaced.

If the metacone is lingually inclinded, the relative position of the metacone to the paracone moves buccally during wear. Thus, this character is determined based on the slightly worn specimens.

104. M1-2 postmetacrista: (0) short; (1) relatively long; (2) distinctly long; (3) reduced to a ridge or almost absent. (modified from DH97:15, C05:27)

If the postmetacrista is considerably shorter than the distance between paracone and metacone on slightly worn M1-2, it is coded as short (state 0).

105. Orientation of M1 postmetacrista: (0) posteriorly; (1) slightly posterobuccally; (2) considerably posterobuccally. (modified from RH14:143, in part)

106. Orientation of M2 postmetacrista: (0) posteriorly; (1) slightly buccally deflected; (2) strongly buccally deflected.

107. M1-2 paraconule: (0) large and distinct; (1) small or indistinct, (2) absent. (RH14:146, in part);

108. The paraconule position: (0) midway between paracone and protocone; (1) closer to protocone, (2) closer to paracone. (RH14:147, in part)

109. M1-2 preparaconule crista orientation toward: (0) preparacrista; (1) curved

towards the paracone without a break.

110. M1-2 protoloph (or the preparaconule crista) on the buccal side: (0) low, separate from the ectoloph by a groove; (1) low and unnotched; (2) high (modified from F02:54, H10:2, DR13:36)
111. M1-2 preprotocrista: (0) slightly notched, (1) complete. (modified from F02:45)
112. M1-2 antecrochet: (0) absent; (1) present and weak; (2) present and prominent.

If there is groove between the protocone and the antecrochet on the posterior surface of the protoloph as in *Hyracodon* and *Teletaceras*, the character state is coded as "present and prominent" (state 2). If the protocone and the antecrochet are generally continuous on the posterior surface of the protoloph as in *Amynodon*, the character state is coded as "present and weak" (state 1).

113. M1-2 protocone lingual surface: (0) nearly vertical; (1) moderately buccally tilting; (2) strongly buccally tilting.
114. M1-2 protocone position: (0) directly lingual to the paracone; (1) posterior to the paracone, closer to the paracone than to the mesostyle (or the middle point of the centcrista); (2) anterior to the paracone.
115. M1-2 metaloph (or premetaconule crista) joins the ectoloph: (0) low, near the base of the metacone; (1) high, nearly at the apex of the metacone; (2) high, considerably anterior to the metacone, even towards the middle of the centrocrista;

In Lophialetidae (*Schlosseria*, *Lophialetes*, *Eoletes*, *Kalakotia*), the metaloph contacts the ectoloph distinctly anterior to the metacone, nearly at the middle point of the centrocrista (state 2), while in most ceratomorphs the metaloph contacts the ectoloph near the apex of the metacone (state1). This character can only be determined in slightly-worn teeth, because as wear progresses, the metaloph and ectoloph junction can be changed as, for example, in the M3 of *Moropus elatus* (Coombs, 1978).

116. M1-2 metaconule: (0) present; (1) very small; (2) indistinct or absent. (modified from RH14:148)
117. M1-2 metaloph: (0) extremely oblique; (1) transverse to slightly oblique. (B10:22; H10:35, in part; F02:58 in part)
118. M1-2 crochet: (0) absent, (1) present.
119. Metaconal fold: (0) present; (1) absent. (H10:32)

M3

120. M3 outline: (0) quadrilateral; (1) roughly square; (2) triangular.
121. M3 parastyle position related to the paracone: (0) anterior; (1) slightly anterobuccal; (2) strongly anterobuccal; (3) anterolingual.
122. M3 parastyle: (0) aligned with those on M1-M2, (1) projecting strongly buccally. (modified from HD04:43)
123. M3 parastyle: (0) not recurved; (1) posteriorly recurved. (HD04:42, H09:44, B10:14, RH14:152)
124. M3 mesostyle: (0) absent; 1) small, cingulum-like.
125. M3 metacone: (0) as large as the paracone; (1) somewhat smaller than the paracone, but still distinct; (2) vestigial; (3) absent, confluent with ectoloph and metaloph.
126. M3 metacone position: (0) equivalent to M2 position, (1) lingually shifted relative to M2 metacone position, (2) shifted buccally. (modified from RR15:67, F02:61, H09:48)
127. M3 postmetacrista: (0) short; (1) long; (2) reduced to a ridge; (3) absent. (modified from DH97:15, C05:27, F02:62)
128. Orientation of M3 postmetacrista: (0) posteriorly; (1) buccally deflected; (2) posterolingually.
129. M3 paraconule: (0) large and distinct; (1) small or indistinct, (2) absent. (RH14:146, in part)

130. M3 paraconule position: (0) midway between paracone and protocone; (1) closer to protocone. (modified from RH14:147, in part)

131. M3 preparaconule crista orientation toward: (0) preparacrista; (1) curved towards the paracone.

132. M3 protoloph (or the preparaconule crista) on the buccal side: (0) low, separated from the ectoloph by a groove, (1) low and unnotched; (2) high. (modified from F02:54, H10:2, DR13:36,)

133. M3 Preprotocrista: (0) slightly notched, (1) complete. (modified from F02:45)

134. M3 protocone lingual surface: (0) vertical; (1) slightly buccally tilting; (2) strongly buccally tilting.

135. M3 protocone position: (0) directly lingual to the paracone; (1) posterior to the paracone, closer to the paracone than to the mesostyle (or the middle point of the centrocrista); (2) anterior to the paracone.

136. M3 metaconule: (0) present and relatively large; (1) small; (2) indistinct or absent. (modified from RH14:148, in part)

137. M3 hypocone position: (0) at about same level as protocone; (1) buccally shifted relative to protocone; (2) lingually shifted relative to protocone. (RH14:164, in part)

138. M3 metaloph: (0) transverse or slightly oblique; (1) extremely oblique. (modified from B10:22; H10:35, in part; F02:58 in part)

When the metaloph is nearly in line with the ectoloph and extends towards the paracone, it is also coded as oblique (state 1).

139. The degree of M3 metaloph confluence with the ectoloph: (0) absent, (1) moderate, (2) complete.

Lower Dentitions

Lower incisors

140. The number of lower incisors: (0) 3, (1) 2 (i1-i2), (2) only i1 present, (3) only

i2 present.

Only one pair of lower incisors was present in *Cadurcodon*, however, its homology with a particular incisor pair has not been clear. Considering that the primitive amynodonts *Rostriamynodon*, *Amynodon* and *Sharamynodon* have i2 larger than other incisor, it is reasonable to deduce that the lower incisors of *Cadurcodon* are i2 (state 3).

141. Where three incisors are preserved, they are: (0) approximately equal in size, (1) enlarged from i3 to i1, (2) enlarged from i1 to i3, (3) i2 the largest, (4) i1 and i2 equal insize, i3 much reduced

One specimen of *Homogalax protapirinus* (AMNH FM 131) shows that the lower incisors increase in size from i1 to i3 (state 2), which is in contrast to the subequal lower incisors as mentioned by Radisnksy (1963).

142. The arrangement of lower incisors: (0) loosely arranged, (1) closely arranged, (2) widely separated.

143. Lower incisors: (0) arched; (1) converging anteriorly and forming an acute angle.

144. Lower incisors shape: (0) spatulate; (1) i3 pointed; (2) all pointed; (3) i2 tusk-like, i1-i3 reduced or absent, (4) i1 hypertrophied and conical, i2-3 reduced or absent.

145. Posterior cusp on i3: (0) absent, (1) present. (RH14:168, H09:50)

146. i3-canine diastema: (0) present (1) absent.

Lower canine

147. Lower canine: (0) present, (1) absent. (AA07:36, CD12:40, coding reversed)

148. Lower canine: (0) relatively large; (1) small; (2) hypertrophied.

149. Postcanine diastema length: (0) less than 50% of m1 length, (1) 50-100% of m1 length, (2) 100-150% of m1 length, (3) >150% of m1 length. (H10:48)

In some taxa (e.g. *Colodon occidentalis*) the lower canines are absent, and the

diastema was measured between the last incisor and the first premolar.

p1

150. p1: (0) present; (1) absent. (HD04:32, C05:49)
151. p1 root: (0) single; (1) double.
152. p1 morpholoogy: (0) simple with anterior and posterior ridges from the protoconid; (1) relatively complex with additional posterolingual ridge from the protoconid.
153. Post p1 diastema: (0) longer than lower postcanine diastema, (1) shorter, (2) absent. (H10:45)

p2

154. p2: (0) present, (1) absent. (BB95:35; AA07:48, coding reversed)
155. p2 lingual cingulid: (0) absent; (1) incomplete, (2) complete.
156. p2 buccal cingulid: (0) absent; (1) incomplete, present on the hypoflexid; (2) interupted at the protoconid; (3) continuous, weak; (4) complete, strong.
157. p2 posterolingual cingulid: (0) absent; (1) present and enclosing the lingual side of talonid.
158. p2 paraconid: (0) absent, (1) present with paracristid, (2) distinct, relatively large and prominent; (3) paracristid forming loop enclosing anterior basin. (modified from RH14:171)
159. p2 paraconid: (0) less than half the height of the protoconid; (1) more than half the height of the protoconid.
160. p2 metaconid: (0) absent; (1) small and appressed to the protocone, (2) distinct, close to the protoconid; (3) distinct, widely separated from the protoconid.
161. Length of p2 trigonid: (0) much longer than talonid, (1) as long as or shorter than talonid. (modified from M08:78)
162. p2 protolophid orientation: (0) nearly transverse, (1) posterolingually, (2)

anteroposteriorly

163. p2 cristid obliqua contacting the trigonid (or protoconid): (0) low; (1) high.

164. The orientation of p2 cristid obliqua: (0) toward the midpoint of the protolophid; (1) toward the protoconid; (2) closer to the protoconid than to metaconid; (3) toward the metaconid.

165. p2 hypoconid: (0) small; (1) large, (2) absent.

A hypoconid lower than half the height of the protoconid was coded as small (state 0). A hypoconid higher than half the height of the protoconid was coded as large (state 1).

166. p2 hypconid position: (0) distocentral, (1) distobuccal. (RR15:77)

167. p2 entoconid: (0) absent; (1) weak; (2) distinct.

168. p2 hypolophid: (0) absent; (1) weak, curves lingually for a short distance; (2) weak, curves along the posterior border; (3) lophid and distinctly lower than protocristid; (4) lophid and approaching the protocristid.

169. p2 talonid: (0) not broader than trigonid; (1) broader than trigonid.

p3

170. p3 size: (0) comparable in size to m1, (1) distinctly smaller than m1. (modified from F02:71)

171. p3 lingual cingulid: (0) absent; (1) incomplete; (2) continous and weak; (3) complete.

172. p3 buccal cingulid: (0) absent; (1) incomplete, present on the hypoflexid; (2) interupted at the protoconid; (3) continuous, weak; (4) complete.

173. p3 trigonid: (0) as long as the talonid; (1) longer than the talonid; (2) shorter than the talonid.

174. p3 trigonid: (0) as long as wide; (1) longer than wide; (2) wider than long.

175. p3 paraconid: (0) absent, (1) indistinct, confluent with paracristid, (2) distinct.

176. p3 paraconid: (0) less than half the height of the protoconid; (1) more than half

the height of the protoconid. (H10:28, similar to C05:50, DH:97:23, HD04:31, HD03:31)

177. p3 paraconid: (0) single cuspate; (1) bifurcate.
178. p3 buccal branch of the paracristid (paracristid I) orientation: (0) anteriorly; (1) anteriorly and slightly lingually; (2) anterolingually; (3) anterobuccally.
179. p3 metaconid: (0) absent; (1) very small; (2) slightly smaller; (3) large. (modified from F02:69)
180. p3 metaconid position: (0) close to protoconid; (1) separated. (similar to RH14:174, F02:70)
181. p3 protolophid orientation: (0) nearly transverse; (1) slightly oblique; (2) considerably oblique.
182. p3 protolophid: (0) notched; (1) lophid.
183. Position of metaconid related to the protoconid on p3: (0) considerably more posteriorly displaced; (1) slightly more posteriorly displaced; (2) transversely displaced.
184. The ridge on the buccal side of the protoconid of p3: (0) absent; (1) present and smooth; (2) present with nodules.
185. p3 cristid obliqua: (0) low but unreduced; (1) high; (2) high and composed of two ridges; (3) low and reduced.

In *Colodon*, *Telelophus*, and *Deperetella*, the cristid obliqua of p3 is composed of two ridges: one extending from the hypoconid anteriorly and the other steep one descending from the apex of the protoconid posteriorly (state 2). The two ridges join at a distinct notch. In *Tapirus*, the cristid obliqua of p3 is highly reduced and joins the posterior base of the protoconid (state 3).

186. Orientation of p3 cristid obliqua: (0) toward the midpoint of the protolophid; (1) towards protoconid; (2) closer to the protoconid than to metaconid; (3) towards the metaconid.
187. p3 hypoconid: (0) small; (1) large. (modified from RH14:176)

188. p3 hypoconid position: (0) posterocentral; (1) posterobuccal.
189. p3 entoconid: (0) absent; (1) present, distinctly smaller than hypoconid; (2) present, comparable in size to hypoconid. (RH14:177)
190. p3 hypolophid: (0) absent; (1) weak with a faint rib, or curves lingually for a short distance; (2) weak, curves along the posterior border; (3) lophid and distinctly lower than protocristid; (4) lophid and approaching the protocristid.
191. p3 talonid: (0) not broader than trigonid; (1) broader than trigonid. (DH97:24, C05:52, in part)
192. p3 postmetacristid: (0) absent; (1) present.

p4

193. p4 size: (0) smaller than m1, (1) comparable in size to m1, (2) larger than m1.
194. p4 lingual cingulid: (0) absent; (1) incomplete; (2) continuous and weak; (3) complete.
195. p4 buccal cingulid: (0) complete (1) continuous, weak; (2) interrupted at the protoconid; (3) incomplete, present on the hypoflexid; (4) absent.
196. p4 trigonid: (0) shorter than the talonid (1) as long as the talonid;; (2) longer than the talonid.
197. p4 trigonid: (0) wider than long (1) as long as wide; (2) longer than wide.
198. p4 paraconid: (0) less than half the height of the protoconid; (1) more than half the height of the protoconid. (H10:28, similar to C05:50, DH97:23, HD04:31, HD03:31)
199. p4 buccal branch of the paracristid (paracristid I) compared with protolophid: (0) shorter; (1) as long as; (2) longer.
200. p4 anterior branch of the paracristid (paracristid II) compared with paracristid I: (0) shorter; (1) as long as; (2) longer.
201. p4 paracristid I orientation: (0) anteriorly; (1) anteriorly and slightly lingually; (2) anterolingually; (3) anterobuccally.

202. p4 metaconid position: (0) close to protoconid; (1) separated.

203. p4 protolophid orientation: (0) nearly transverse; (1) slightly oblique; (2) considerably oblique.

204. p4 protolophid: (0) notched; (1) lophid or shallow notched.

205. Position of metaconid related to the protoconid on p4: (0) considerably more posteriorly displaced; (1) slightly more posteriorly displaced; (2) transversely displaced.

206. Ridge on the buccal side of the protoconid of p4: (0) absent; (1) present and smooth; (2) present with nodules.

207. p4 metaconid twinned: (0) absent; (1) present.

208. p4 cristid obliqua: (0) low but unreduced; (1) high; (2) high and composed of two ridges; (3) low and reduced.

209. The orientation of p4 cristid obliqua: (0) toward the midpoint of the protolophid; (1) towards protoconid; (2) closer to the protoconid than to metaconid.

210. p4 hypoconid: (0) small; (1) large.

211. p4 entoconid: (0) absent; (1) present, distinctly smaller than hypoconid; (2) present, comparable in size to hypoconid.

212. p4 hypolophid: (0) absent; (1) weak with a faint rib, or curves lingually for a short distance; (2) weak, curves along the posterior border; (3) lophid and distinctly lower than protocristid; (4) lophid and approaching the protocristid.

213. p4 talonid: (0) no broader than trigonid; (1) broader than trigonid. (DH97:24, C05:52, in part)

214. p4 hypoconulid: (0) present; (1) absent.

m1-2

215. m1-2 lingual cingulid: (0) absent; (1) incomplete; (2) continuous and weak.

216. m1-2 buccal cingulid: (0) complete; (1) continuous, weak; (2) interrupted at the protoconid; (3) interrupted at the hypoconid;(4) incomplete, present on the

hypoflexid; (5) absent.

217. m1-2 trigonid compared with talonid: (0) shorter, (1) roughly equal in length.

218. m1 trigonid: (0) wider than long; (1) as long as wide; (2) longer than wide.

219. m2 trigonid: (0) wider than long; (1) as long as wide; (2) longer than wide.

220. m2 mean width as percentage of length: (0) >75, (1) 71-74, (2) 69-70, (3) 67-68, (4) 63-64, (5) 59-61. (H10:18)

221. m1-2 paraconid: (0) less than half the height of the protoconid; (1) more than half the height of the protoconid. (H10:28, similar to C05:50, DH:97:23, HD04:31, HD03:31)

222. molar paracristid: (0) present; (1) reduced, (2) absent.

223. molar paracristid I (buccal branch) compared with protolophid: (0) shorter; (1) as long as; (2) longer.

224. molar paracristid II (anterior branch) compared with paracristid I (buccal branch): (0) shorter; (1) as long as; (2) longer.

225. m1-2 paracristid I orientation: (0) anteriorly; (1) slightly lingually; (2) moderately lingually; (3) strongly lingually. (modified from B10:41)

226. m1-2 paracristid with anterobuccal angle: (0) rounded, (1) sharp or bulging. (H94:18, F02:88, HD03:18, HD04:18, H10:16)

227. m1-2 protolophid orientation: (0) nearly transversely or slightly posterolingually oblique; (1) somewhat posterolingually oblique, (2) distinctly posterolingually oblique.

228. Position of metaconid related to the protoconid on m1-2: (0) slightly more posteriorly displaced; (1) considerably more posteriorly displaced; (2) transversely displaced

229. m1-2 premetacristid (anterior crest of metaconid): (0) present, (1) absent. (HD04:41, RH14:189, in part)

230. Molar twinned metaconid: (0) absent; (1) present. (M08:84, H94:4, DR13:64, RH14:186, H09:54 in part)

231. m1-2 protolophid: (0) deeply notched, (1) shallowly notched, (2) lophid. (modified from H09:55, HD03:5, HD04:5, RH14:185, in part)

232. m1-2 trigonid: (0) V-shaped; (1) U-shaped (CD12:54, AA07:50)

233. m1-2 talonid: (0) not broader than trigonid; (1) broader than trigonid. (DH97:24, C05:52, in part)

234. m1-2 cristid obliqua: (0) low; (1) high, (2) highly reduced. (modified from F02:82, HD03:15, HD04:15, H94:15, RR15:91, M08:83)

235. Orientation of m1-2 cristid obliqua: (0) toward the midpoint of the protolophid; (1) toward the protoconid; (2) closer to the protoconid than to metaconid. (modified from F02:80, H10:13)

In *Sifrhippus* and *Protohippus*, the cristid obliqua extends toward the midpoint of the protolophid (state 0); In ceratomorphs, the cristid obliqua extends toward a point buccal to the midpoint of the protolophid (state 2), even to the protoconid (state 1).

236. m1-2 hypolophid: (0) weak and deeply notched; (1) shallowly notched; (2) lophid.

237. m1-2 hypolophid orientation: (0) nearly transversely; (1) slightly posterolingually oblique; (2) strongly posterolingually oblique. (modified from F02:92)

238. m1-2 hypoconulid: (0) small, (1) absent or cingular. (modified from RH14:201, similar to DR13:66)

239. m1-2 posthypocristid: (0) present; (1) absent. (RH14:198, in part)

240. m1-2 hypolophid and cristid obliqua junction: (0) angled, (2) curved.

241. m1-2 postmetacristid: (0) absent, (1) present.

242. molar entoconulid: (0) indistinct or absent (1) distinct. (HD03:16, HD04:16, H10:14, RH14:207, in part)

243. molar entocristid: (0) absent; (1) present and anteriorly extended; (2) present and curves slightly lingually. (modified from RR15:101)

m3

244. m3 talonid: (0) narrower than trigonid; (1) as broad as the trigonid; (2) broader than trigonid. (modified from DH97:24, C05:52, in part)

245. m3 trigonid compared with talonid: (0) shorter, (1) roughly equal in length.

246. m3 lingual cingulid: (0) absent; (1) present but incomplete.

247. m3 buccal cingulid: (0) complete; (1) continuous, weak; (2) interrupted at the protoconid; (3)interrupted at the hypoconid (4) incomplete, present on the hypoflexid; (5) absent.

248. m3 length: (0) short; (1) long. (M08:86)

Length of crown is more than twice the width (state 1).

249. m3 paraconid: (0) less than half the height of the protoconid; (1) more than half the height of the protoconid. (H10:28, similar to C05:50, DH:97:23, HD04:31, HD03:31)

250. m3 paracristid: (0) present; (1) reduced; (2) absent.

251. m3 paracristid orientation: (0) anteriorly or slightly lingually, (1) anterolingually, (2) lingually. (B10:41)

252. m3 paracristid with anterobuccal angle: (0) rounded, (1) sharp or bulging. (H94:18, F02:88, HD03:18, HD04:18, H10:16)

253. m3 protolophid orientation: (0) nearly transversely; (1) somewhat posterolingually oblique, (2) distinctly posterolingually oblique.

254. m3 protolophid: (0) deeply notched; (1) moderately notched; (2) shallowly notched; (3) lophid. (modified from H09:55, HD03:5, HD04:5, RH14:185, in part)

255. m3 premetacristid (anterior crest of metaconid): (0) absent, (1) present. (HD04:41, RH14:189, in part, coding reversed)

256. m3 trigonid: (0) U-shaped; (1) V-shaped. (CD12:54, AA07:50)

257. m3 metaconid buttress: (0) absent; (1) lingual, (2) buccal. (HD04:13, H10:12,

RH14:188, in part)

258. m3 cristid obliqua: (0) low; (1) high, (2) highly reduced. (modified from F02:82, HD03:15, HD04:15, H94:15, RR15:106, M08:83)
259. m3 cristid obliqua: (0) straight; (1) bowed buccally. (modified from H10:4)
260. Orientation of m3 cristid obliqua: (0) toward the midpoint of the protolophid; (1) toward the protoconid; (2) closer to the protoconid than to metaconid. (modified from F02:80, H10:13)
261. m3 posthypocrisitd: (0) present; (1) absent.
262. m3 hypolophid: (0) deeply notched; (1) shallowly notched; (2) lophid. (modified from F02:104, H09:58, HD03:28, HD04:28, H94:28)
263. m3 hypolophid orientation: (0) nearly transversely; (1) slightly posterolingually oblique; (2) strongly posterolingually oblique. (modified from F02:92)
264. m3 hypoconulid: (0) lobe-like; (1) small and reduced, (2) absent or cingular. (modified from DH97:16, RH14:204, H09:59, similar to DR13:67)
265. m3 hypoconulid: (0) high, nearly as high as the talonid; (1) low, much lower than the talonid. (B10:39)
266. m3 hypoconulid buccal border: (0) toward the hypoconid; (1) toward the midpoint of hypolophid. (RD13:70, F02:97, modified from RH14:206)
267. m3 hypoconulid lingual border: (0) absent; (1) crest-like.
268. m3 hypoconulid lobe buccal cingulid: (0) distinct; (1) absent or weak.
269. m3 hypolophid and cristid obliqua junction: (0) angled; (2) curved.
270. m3 postmetacristid: (0) absent, (1) present.
271. Molar cristid obliqua compared with the hypolophid: (0) shorter; (1) equal; (2) almost twice as long.

Cranial characters

272. Skull type: (0) dolichocephalic; (1) mesaticephalic; (2) brachycephalic.

If the facial and cranium portions were demarcated by the postorbital process of

the frontal (Qiu and Wang, 2007), dolichocephalic refers to the facial portion longer than the cranium (state 0), mesaticephalic means facial as long as cranium (state 1), brachycephalic refers to facial shorter the cranium (state 2).

273. Dorsal surface of skull from the lateral view: (0) nearly flat above the orbits and postorbital region; (1) nearly flat above the orbits, strongly convex over the postorbital cranial region; (2) entire dorsal surface of the skull is concave, or saddle-shaped; (3) generally convex and arched. (modified from M08:20)

Occipital region

274. Outline of the occipital: (0) bell-shaped and wider than high; (1) bell-shaped and higher than wide; (2) trapezoid and higher than wide; (3) trapezoid and wider than high; (4) triangular.

275. Occipital: (0) the upper part projected posteriorly but not beyond the occipital condyles; (1) the upper part projected posteriorly beyond the occipital condyles. (modified from H89:3, AA07:18, CD12:19, similar to M08:41)

276. Occipital condyle: (0) not high and almost rounded; (1) elongated vertically. (RR15:128, AA07:19, BB95:21, CD12:20)

277. Proportions of occiput: (0) dorsal portion narrower than ventral portion; (1) dorsal portion as wide as the ventral portion. (modified from M08:36)

278. The exoccipital (and/or supraoccipital) above the foramina magnum: (0) anteriorly inclined; (1) nearly vertically displaced or slightly anteriorly inclined.

Since the sutures between the exoccipital and supraocciptial were usually obliterated, this character refers to the bone between the excavated dorsal part and foramen magnum.

279. Occipital condyles in lateral view: (0) projected posteriorly to the exocciptial; (1) not projected behind exoccipital. (modified from AA07:14, BB12:15)

280. The distal extension of the paroccipital process: (0) short, above the level of the ventral border of the occipital condyle; (1) long, slightly beyond the occipital

condyle; (2) considerably beyond the occipital condyle.

Basicranium

281. Hypoglossal foramen position between occipital condyle and paroccipital condyle: (0) closer to occipital condyle; (1) equal distance; (2) distant from occipital condyle and closer to paroccipital condyle.
282. Basisphenoid orientation from the basioccipital: (0) rising dorsally; (1) horizontal.
283. Posterior extension of jugal: (0) onto to or just in front of the glenoid fossa; (1) separated from the glenoid fossa by a fossa;
284. Posterior end of jugal: (0) narrow and ridge-like; (1) wide and plate-like.
285. Basioccipital: (0) smooth; (1) with a blunt median ridge; (2) with a sharp median crest. (modified from AA07:25, BB95:17, CD12:26, character state reversed)

In rhinocerotids the basioccipital bears a sharp median crest (state 2).

286. Orientation of basioccipital: (0) rising anterodorsally; (1) nearly horizontal.
287. External auditory pseudomeatus: (0) mediolaterally angled; (1) posterolaterally angled. (modified from M08:32)
288. Exposure of mastoid: (0) narrow, laterally; (1) absent (amastoidy). (modified from RH14:28)
289. Interspace between occipital and squamosal for the mastoid process: (0) short, on the ventral part between occipital and squamous; (1) long and slit-like; (2) a narrow dorsal part present between occipital and squamosal, with the ventral part concealed.
290. Mastoid foramen: (0) present, between mastoid and occipital-supraoccipital; (1) absent. (RH14:29)
291. Paroccipital-posttympanic foramen: (0) absent; (1) present.

Qiu and Wang (2007) named a foramen between the paroccipital process and

posttympanic process as the paroccipital-posttympanic foramen (Qiu and Wang, 2007, Pl. 1, fig. 2). A similar foramen is also present in *Pappaceras*, *Paraceratherium*, *Teletaceras*, *Sharamynodon*, *Amynodon*, and *Metamynodon*.

292. The paroccipital process and posttympanic process: (0) separate; (1) fused.

Qiu and Wang (2007) determined that the coalesced and enlarged paroccipital and posttympanic processes is one of the most important features of *Juxia* and *Paraceratherium*. We define this character as the anterolateral face of paroccipital process completely appressed to the posttympanic process at least on the proximal part (state 1).

293. Position of the paroccipital process related to the posttympanic process: (0) posteromedially; (1) medially; (2) posteriorly;

In *Pappaceras*, *Juxia*, *Paraceraterium*, and *Sharamynodon*, the paroccipital process is displaced posterior to the posttympanic process (state 2).

294. Posttympanic process and paroccipital process: (0) relatively slender; (1) transversely wide; (2) anteroposteriorly elongated (W16:43)

295. Ventral extension of paroccipital process relative to the posttympanic process: (0) approximately equally extended; (1) considerably longer.

296. Ventral extremities of postglenoid process and the posttympanic process: (0) widely separated; (1) close but not fused; (2) fused.

297. Ventral extension of posttympanic process: (0) slightly shorter than postglenoid process; (1) considerably shorter; (2) as long as or more ventrally extended. (modified from RH14:27, H09:12, RR15:137, H01: C13)

298. Postglenoid process to occipital condyle length, relative to the length of cranial post-P1 portion of skull: (0) short (<1/5), (1) long (>1/5). (W16:38)

299. Postglenoid foramen: (0) present; (1) absent. (RR15:119, RH14:26, H09:11,C05:81, H01:C10)

300. Postglenoid process facing: (0) anteriorly; (1) anterolaterally; (2) nearly laterally. (modified from RR15:120, RH14:25, C05:86, H09:10, H01:C11).

In amynodotids and paraceratheres, the postglenoid process is divided into roughly equal-sized anterior and lateral facing parts, thus coded as anterolaterally (state 1). In *Subhyracodon*, and *Trigonias* the anterior part is considerably smaller than the lateral part, and main orientation is laterally (state 2). This character is probably related to the well-developed postcotyloid process in rhinocerotids.

301. Anterior face of the postglenoid process: (0) flat or concave and undivided; (1) convex with a median ridge. (H01: C12).
302. Postglenoid process: (0) relatively small and peg-like; (1) flattened and transversely extended; (2) considerably elongated and twisted; (3) massive and relatively short.
303. Temporal condyle of mandibular fossa: (0) flat or slightly concave; (1) slightly anteroposteriorly convex;
304. Orientation of the temporal condyle: (0) anterolaterally; (1) transversely.

In paraceratheres and amynodonts, the temporal condyles are anterolaterally inclined (state 0), while in rhinocerotids the temporal condlyles are transversely extended (state 1).

305. Foramen ovale: (0) separate from middle lacerate foramen, (1) confluent with middle lacerate foramen. (RH14:18, C05:82, M08:33, similar to DR13:5, H89:1)

Although MacFadden (1976) used the confluence of foramen ovale and foramen lacerate medium as a synapomorphic character of *Hyracotherium* and Equidae, there is no confident evidence to support the confluence of these foramina in early *Hyraocotherium*-like taxa. In contrast, Gingerich (1981) observed the separate condition present in some *Hyracotherum*-like specimens. We coded *Protorohippus venticolum* as state 1, although the condition was poorly preserved in AMNH 4832.

Although Scott (1941) stated that *Hyracodon* has the foramen ovale fused with posterior opening of the alisphenoid canal (= foramen rotundum of Scott, 1941), it is more likely that the foramen ovale of *Hyracodon* is fused with the middle lacerate

foramen (AMNH 12303).

306. Pterygoid fossa: (0) absent; (1) present. (W16:49)

In *Hyracodon* in ventral view (AMNH 12303), the pterygoid fossa is situated on either side of the basisphenoid and presphenoid, and bordered laterally and medially by distinct ridges. On the posterior side, the fossa is open to the middle lacerate foramen. On the posterolateral side, the fossa is separated from the glenoid fossa by an arterial groove. *Lophialetes* and *Schlosseria* have similar pterygoid fossae (state 1) (Li and Wang, 2010), however, the foramen ovale is separated from the middle lacerate foramen.

307. 'Temporal crest': (0) weak; (1) absent; (2) prominent.

The temporal crest refers to the ridge between the nuchal crest and the zygomatic arch (Bai et al., 2017)

308. Frontal ridges: (0) converging into a median sagittal crest posteriorly just behind the orbit, (1) converging more posteriorly, (2) separated as two ridges. (modified RR15:122)

309. Sagittal crest: (0) low; (1) high.

Wall (1982) noted that the sagittal crest of amynodonts is high and laterally compressed compared with relatively low sagittal crests of other perissodactyls. *Pappaceras* and other paraceratheres also have high sagittal crests.

310. Squamous-frontal contact: (0) absent; (1) present.

Orbit and side wall of braincase

311. Sharp crest extending from the orbital foramen anteriorly: (0) absent; (1) short; (2) long.

312. Orbital portion of parietal: (0) contacting alisphenoid; (1) not contacting alisphenoid (RH14:20)

313. Orientation of the jugal-squamosal suture with respect to the horizontal plane: (0) parallel; (1) oblique. (AA07:9, CD12:9)

314. Ventral postorbital process: (0) absent; (1) weak; (2) prominent.

315. Zygomatic process of squamosal contributes to the ventral postorbital process: (0) no; (1) yes.

316. Posterior part of zygomatic arch: (0) always lower than the level of the upper border of the orbit; (1) reaching the same level as the upper border of the orbital. (AA07:11, CD12:12, RR15:127 coding reversed)

317. Postorbital process of the frontal: (0) weak or absent; (1) protuberant as an elongated flange; (2) prominent and overhanging the orbital, but not protruding from the frontal; (3) sturdy and short; (4) forming the bar in contact with zygomatic arch.

318. Supraorbital foramen or notch: (0) absent, (1) present. (H01:C9, RR15:118; RH14:13, coding reversed)

319. Anterior opening of alisphenoid canal: (0) in common with foramen rotundum; (1) completely isolated.

320. Foramen rotundum: (0) opening in common with the orbital foramen; (1) opening in common with the anterior opening of the alisphenoid canal. (modified from RH14:15).

321. Position of the optic foramen: (0) approximately in middle between ethmoid foramen and orbital foramen; (1) closer to the orbital foramen than to the ethmoid foramen; (2) closer to the ethmoid foramen than to the orbital foramen. (modified from HR14:14, H10:29, HD03:33, HD04:33, H89:2)

322. Position of the sphenopalatine foramen: (0) far away from the maxillary foramen; (1) below the maxillary foramen. (modified from RH14:8, H09:4)

323. Retromolar space on the maxilla: (0) absent; (1) present. (AA07:22, BB95:4, CD12:23)

324. Anterior border of the orbit at the level of: (0) near the boundary of M2/M3; (1) near the border of M1/M2; (2) near the border of P4/M1; (3) near the border of P3/ P4.

325. Zygomatic arch: (0) nearly parallel to the midline of the skull; (1) straight but anteriorly convergent; (2) convex.

326. Posterior end of zygomatic arch lobe-shaped blade: (0) absent; (1) present.

Qiu and Wang (2007) concluded that the posterior end of the zygomatic arch in *Juxia* is a lobe-shaped blade protruded upward, with a steep anterior rim (state 1). In *Fostercooperia*, it is rudimentary.

327. Postorbital width of skull: (0) constricted; (1) not constricted (M08:22)

Facial portion

328. Anterolateral apophyses of the nasal: (0) present; (1) weak or absent.

329. Narial incision: (0) above or anterior to canine; (1) above diastema between canine and premolars; (2) above P1 and P2; (3) above P3 and P4; (4) above molars; (5) retracted and excavated posteroventrally. (modified from DR13:1, C05:56, H01:C8).

330. Free end of nasal extending above the level of: (0) canine or premaxilla and maxilla suture; (1) anterior end of the premaxilla; (2) P1; (3) around P4/M1; (4) postcanine diastema.

331. Posterior end of nasal: (0) above the center of the orbit; (1) near the anterior border of the orbit; (2) considerably anterior to the orbit; (3) posterior to the orbit.

332. Premaxilla and nasal contact: (0) present; (1) absent. (RH14:4, modified from C05:60)

333. Anterior ends of premaxillae: (0) unfused; (1) fused.

334. Lacrimal tubercle: (0) weak or absent; (1) distinct with a single tubercle; (2) distinct with two tubercles.

335. Nasolacrimal contact: (0) absent; (1) present. (CD12:3, AA07:3; H01:C3, RR15:115, coding reversed; modified from F02:4)

336. Facial exposure of the lacrimal: (0) large; (1) smaller or reduced. (RR15:123,

H01:C3, F02:4, H09:5)

337. Lacrimal foramen: (0) single; (1) two.

338. Narrow grooves on posterolateral nasals and frontals at the posterior terminus of the narial incision: (0) absent; (1) present, short, largely restricted to posterolateral nasals; (2) present, long and straight, extending well onto frontals. (C05:73)

339. Medial grooves on dorsal surface of frontals: (0) absent; (1) present. (C05:75)

340. Frontal between two orbits: (0) relatively narrow; (1) expanded;

The frontal width between orbitals narrower than the zygomatic arch width at the level of the orbitals (state 0). equal or wider than zygomatic arch (state 1)

341. Maxillary fossa (preorbital fossa): (0) absent; (1) present.

Pappaceras, paraceratheres, and amynodonts possess maxillary fossae (state 1). In some tapiroids, the maxillary fossa is also present; however, its homology with those of rhinocerotoids are uncertain.

342. When present, maxillary fossa: (0) shallow; (1) well-developed pocket; (2) vertical groove near the orbit. (H01: C6; modified RR15:117)

343. Infraorbital foramen: (0) above P4/M1; (1) above P3/P4; (2) above P2/P3; (3) above P3; (4) above P4; (5) above M1/2; (6) above M2/3. (modified from H01: C7)

344. Paried horns: (0) absent; (1) present on nasal.

Palate

345. Palatine process of premaxilla: (0) absent, (1) present

In *Juxia* and *Paraceratherium* the palatine process of the premaxilla is absent (state 0), while in *Pappaceras* and *Rostriamynodon*, the palatine process is present (state 1).

346. Incisive foramen: (0) paired, fissure-like; (1) single, median. (modified from H01: C5)

347. Posterior border of palatine at the level of: (0) near the border of M2 and M3; (1) near the border of P4 and M1; (2) near M1/M2; (3) posterior to M3. (modified RR15:134, AA07:23)
348. Body of the premaxilla: (0) slender; (1) stout, laterally expanded; (2) stout, laterally compressed;

In *Pappaceras* and some amynodonts, the horizontal process of the premaxilla is stout and laterally expanded in dorsal view (state 1). In *Paraceratherium* and *Subhyracodon*, the horizontal process of the premaxilla is stout and somewhat laterally compressed (state 2) related to the reduction of incisors.

Mandible

349. Height of the mandibular corpus: (0) constant; (1) increasing posteriorly. (AA07:30, BB95:25, CD12:32, B10:54, RR15:147)
350. Body of mandible: (0) anteriorly extended with incisors procumbent; (1) anterodorsally extended with incisors more erect. (modified from CD12:35)
351. Posterior border of the mandibular symphysis: (0) anterior to p2; (1) anterior border of p2; (2) near p2/p3; (3) near p3/4. (modified from AA07;33, BB95:24, CD12:37, RR15:143)
352. Vascular impression of mandible: (0) moderately developed, (1) weak or absent.
353. Angle of mandible: (0) not expanded; (1) strongly posteriorly extended; (2) moderately posteriorly extended. (modified from B10:55, AA07:31, BB95:26, CD12:34, RR15:148)
354. Retromolar space on the mandible: (0) absent; (1) present. (AA07:32, CD12:36, B10:56
355. Anterior border of ascending ramus: (0) slanted posteriorly; (1) nearly vertical; (2) convex anteriorly.
356. Posterior border of ascending ramus: (0) rounded convex; (1) slanted anteriorly;

(2) vertical.

357. Posterior border of ascending ramus medial inflection: (0) absent; (1) present.

358. Condylar process of mandible: (0) low; (1) high.

If the distance between the condylar process and the alveolar border is less than the length of m2-m3, the condylar process is low (state 0) (Qiu and Wang, 2007). If the distance between the condylar process and the alveolar border is greater than the length of m2-m3, the condylar process is high (state 1) (e.g. *Equus*).

359. Postcotyloid process: (0) absent; (1) present. (H01:C14)

360. Coronoid process: (0) posteriorly projected at the apex; (1) vertical.

361. Mandibular notch: (0) wide, (1) shallow.

Supplementary Note 3.

Data matrix

Sifrhippus_sandrae

?????????????????????????????????1010110000?012100000--00031101000001
0020001000--100(01)1012(01)01-00110(01)31001100010010000000010001
10000010?00010???1??????????????
?????????????03000310011102010100001?000011103010010100101000000
001-0-???
?????????????????

Protorohippus_venticolum

0??0?0?00?00000013011000-10-0---?0000110000?10201000---00102001000
001?00200?1000--010000001?1-011001103011000100?00000?0100000000
0010?0000?00010?000300010??????????????002000--131???0?001011?0000
0000111?100?001101000000?00021001??0??00000-0011000000010100?00
0?0000110001000001010000??10000???0000?00000??0??0?11001?01001101
10?010110?01?000??010??0?0001101?01000

Orientolophus

??
???1030110001-(01)010001100001101002001001???????????????????????????
??
??
???

Cardiolophus_radinskyi

02?00100011????000-?-010-0--0----1010110100121?011?00--210010010110
0101020121(01)00--01(01)30110001-011002110000110100?10110(01)010
01000011010?1100?0210??001000000?????0?-?11????00?0???0120200??101
00?00??0??0211101?0020000?000001?00?01021001002110100?110?01?001
100000021110001100000?100110100?2001-?000000200001010?200?1?01?
020?????1100101?0?0??00000-20??00????????????

Homogalax_protapirinus

000??100(01)10000012101?11112100----1010111100121(12)001-00---1000
0010110000102012(12)100--01(01)(23)0000011-011000310011110000?10
110(01)020000000011000?1120?02100?100000?001012?01-0-100000020121
002020(01)0101100000120010201101000(02)01101000001000211021101
0021101000(01)1001000011100?0(01)21110001100000?????????1??????????
????0??010100200???01?020?????1100?0??0?1???0000-20??000111?0???????

Chowliia_laoshanensis
???????0?11000?00(01)???110-0--0---???10???0?01(02)??00?-00---100100301
000011021112000--100(03)0010001-0110003101001102002111100021000
0021102020120?0?1???10?000?000002101-0-00000003112000112-0000100
1000011111001101010000201000002100(12)010211010021101001110011
10000000201010100111001??
???????????????????????????11??????????

Meridiolophus_expansus
??
???300?10000
1001-1-0100000211210?20--?01310001000311110011010012022110(03)00
00?0011?10100110210010000?10?1000?10000002111??????????????????????
???

Isectolophus_latidens
??????20110?0??0020110?0-10-0---01000110000(01)21?0(01)1?(01)(03)(012
)0110(01)10010000?00(12)040121100--11130110001-0110003000?02-0?10
?1?210?0100010002-021?(02)220?0?????????00?(02)0???1?01-?-?00?00??011
?00202?00?30?0010011001020010100?0002010(04)0?04?00111101011002
21010001110(15)100011200?012?11000110001000?01????1?110????????02?
010101?0201???010120?????2000??1?0?11??0000-10???0002??????????

Karagalax_mamikhelensis
??????0111???020-1-????2100--0-101011?1001212111-00--2?00100101100
0?1020111000--0?(01)30010001-0001?01?0000110200?(12)(01)(12)100000
00000011020?(12)(12)0000???0?????000201212001-0111001002112113202
001011000?0012011100100210020(01)1000000051000(01)102101100211
0100020000110000210000211000110001??????????????????????????????0???0???
?????????????????1????0????0??0?????1?????0?0??????????

Gandheralophus
????????????????????????????????10101100?0121?011000--?11000010?10000
1020112000--01(03)(03)1000001-000100310000100200121210000010100
0100201221000310??100300?20???????????????0020?210130101111010011
0010101000002000200011030002?00101021011001?011010111031?00101
00000111000012010???
???????????????????????????????

Yimengia
02011110100????122000?0122200--0-1010111001212000000--?10010101
1100012030023000--1?121112002002?1000111222-(01)210201210001100

10022-121202201001 0??10131---01313001-11110010130110032021101(01
)1102100111201101111001210211040002 0000(01)10010111112111(01)0
00?00400000002000021211----100??
?????????1????????????????102?????11???????????

Rhodopagus_pygmaeus
???????01????????????????????????101000110022100 00?00--21000000011000
12010123000--11241010001-1001000120212-121010121012(03)01021222-
121102012??100??0?31---00002101-(01)1110000100111030----011111(01)1
000400111311110011111110500030002111010 11(01)012111(01)0002005
00001120000011212----100??
??????????????????????????1????????????

Indolophus
???????????????????????????????????0??11???????2?????????100110101?0001201
1011000---13401??0?0-00?000320?112-0110?00200???????????????????????????
??
??
???

Heptodon
0000?210110000?12(01)01?0(01)11(23)(12)00----10101101001214011200--
21001001001000120401221 00--0113100?001-0000000100222-1210?01210
10100010012-12110200000000?1002(01)0?200001?01-??000210000111003
1101012010000002100020110100020000103000200021102001102111110
002100300001020002-112011---1000000001011?000010???000100001010
100201?1?010130010001000111101001?0000-(1 2)01?010(0 1)(0 1)011(1
2)(0 1)1(0 1)0(0 1)0

Helaletes_nanus
???????01100(01)0?12201111112110---?10101111001212(01)1000(13)--210
?1101011001020211 21002--01132112001-0110000111212-0210?21210101
01010002-0211221010?1????0?21-?-000?1111-??1100000201110031201012
1110100021000201101001001001050003000211021011021111100021005
00000020002-112011---100??0??30???
?11??0?3??1?00100001200????1?2??1???????

Desmatotherium_intermedius
???????0110????13201100112100---?10001111002211(01)010130121001001
11100122020121113010113111 2001-1010000111202-021010121010 00101
0002?021122?00??????????1-?-000?3011-0?11011000011100311010021 1111
000201001011110002 11?110?0002000(02)01021021021211110021005000

00030002-11212----100??
????????????????0???????0??????????

Paracolodon_fissus
???????01??????13200021122101110-1110111100221000111301220001011?
1001220001200130101 1(34)1000001-1110000220112-0210121210101010
20212-021122001?????????????00002101-111010?00000110031101002111
0000040010211101000211011050001000211021011022211 1-002?0050000
1020002--1212-----00??????????????????????0???0??????????????????????????????
??????????????????????????????????

Dilophodon_minusculus
???????01?000????1???111?3??0---?0010111100221?00001011????????????????
????????????11420?20?1-10?0?001111?2-0210?012?01????????????????????????
???0?31-?-0001??01-1?110000000111003111101111011100200102011 1100
11111110500030002111010110212111100210050000012 00?2-1?212----10
0???1
01??????????

Selenaletes_scopaeus
???
???
???05000(45)100(12)11101
01100(12)2111(01)002??05000010200???????2----?0????????????????????????
???

Colodon_occidentalis
?0???(14)?01?110101330012012230111201000311000221100111300 01002
121110001220110211130011142000011-1000000120202-02101012101000
0010002-021222001011000-1-31-?-00001121111112010002(02)110031111
02111211000400102011110021122110500000 2----02002?02121111002000
50-2--020-02-111111---10020110011020102 1????000102011010??0001???
00-100????020001433112???0001210??11011120200 10?0

Plesiocolopirus_hancocki
???????01?00000133011101 1?2?0--1010101110001212011000--?10301011?
00010202012 1100--011(34)20000?1-100?003110212-021002121010001012
00?-021?22001?????????21-?-00001121 11111(01)0100202110031 1?1012112
?1000?10102111010001110110?0000010211021021021211 1100200?20001
11201?2-112111---100???????????????????????????????????0????????????????????
???????????101210??11100??0???????

Hesperaletes_borineyi
???????01?11(01)??12101??11121(01)0--(01)?101011?101221(02)011?00--21
032101??10?1?202012??00--0?132112001-(01)00??03?110?2-021??2221010
101012002-021?2200101?00?100300120?????2?1????0?10??????0??1101?????
?21?0??1?????1????0?1?(01)2???40?0??0????0??0111201111?0?100??0??0?02?
002?011?1----?0?00?????????????????????????01010?10201???????11????01100
130?1?2??0210122?1102?????????????

Irenolophus
01?1?010111112113302020103100--11001030101?0112001204--02110123
0101?2?01?0022?04--112(03)111?011-1000000020?22-0210?012101000002
0202-1210222010110??10?301?2014111211111001014021?00311110111
100100100000101111001210011040001010111101021 02-2111-000000500
101130002--1212----10000?000100?0??111000?000100001010?0??01??????
30????01??011110?0??00000-10??0?010??011?10?0

Telelophus_medius
?????1?011111211330101010 3300--110010301000010100120 3--021001200
10002?2110022?03--11201110011-1000000-232-2-021000121010 00003-3-2
-1210222020?10011013011201411121111110000140011103111102111101
0000000020111100221111103000101021110102102-2111-00000030020-0
30-02--1212----100??????????????????????0???0???????????????????????????????
??????????????????0??10102?1???

Deperetella
???????????????13300020103200--11001130100001220012 05--0210?1201?00
0202220022105--21101000011-1000000--31-2-?21000121010 00003-3-2-12
10222020310??10010112024121211111104003401211031111021 1123100
30000000101200221 2301(12)000000100211010 2102-2111-000001000111
120-02--1112-----00??
??????????????????000??????????

Minchenoletes_erlianensis
???00010201111001 2020
013000--11142002001-0001000111112-1210202210100000000102-1212020
00??04
00??10011110101101121011001??
??

Schlosseria_magister
???????0111102?13202011113210--111111131110121201120 0--010112030
1110012030122000--11132112001-0000000112112-021012220000 1000001

02-0211221100??????0??01?2000?1111-02010000020121003110100001101
00021211001101000211011041?1?10101102101101221010000(01)0105000
000100?0021200111200 1??????????????????????????0000??01?0?0?1?0?0200
1?0?????12110?01??000??20????000??0???????

Lophialetes_expeditus
?????1?0111102?132120(12)1113210--11111110111112120 11?00--0201120
0011110120400220 00--11142112001-0001000112212-(01)2102022000000
0000102-1212220110010011013(01)0?200213131002010000020010 00311
0100201101000310?0111101000211001041 11?00101102101101221 01000
00105100000200?002121011120000 0410?110?0??1001-????000?00000?101
201?0??0?020???01010014?11001?1000??400101?????????????

Eoletes
??0????0120?01?13200021113210--110(12)20221110121(23)011?00--22020
012011(12)0012031122000--(01)1132110001-0000000112102-0210002200
10100010102-021?22011??????????????0?21313?10201001002101000 31101
002111010002100100100200020100104101?0010110210111 12?10100000
105000000200?000121011110010 3?100110?????????????0?0?0?00??????01??
??0?040?????0100100?010???000??40??3??????????????

Ampholophus_luensis
???????????????011020110-10-0--0011101101111012111100--220110011010
1011021110000--100200?2011-01?02032001?110200?121101???????????????
??
??
??

Kalakotia
???????????????010-1-110-11-0--0-10011201 1?121201021121?000100201001
012220122000--111221200?1-0001000112112-01??00220010100000102-02
1?02010??????????????????????????????004011100102-0011110211000111100
1101000200201000 00?1001110210010122111000001001000 00100?00212
001111001???
??????????????????????

Breviodon_acares
???????1-------122-1-101-11-0--0-111011010?1210001-00--?000100301?000?
20?0121000--1113?0000?1-0010001200?12-??10?2?200?0100000002-021?2
211??????110031-?-1--?---?-?------???0
31?001001111010?10121111000001040100102 00?01212101102000?0??00
00?20??2101-??02?100?0001?100?01????0?010???02?00?????????1000??????

0?10???0???????

Protapirus
???1?1?01?11010133011001031 00--101001111001?1101?00(01)--?100120
11100101202012200(01)--111320000?1-1?00003011?02-021012121010100
010002-021?2200101100?10131-?-00002130102112110001 11?00311?10?11
1211000410?0201111000212211051000?101110210??0212011100000 1501
1010300?2--1212-----0000110011??011200????100?020010101??200?1?00-0
20????0100013411?10102101210??0?10112?2001??0

Tapirus
031001101111100133011201213013120100011100122120012150012 0322
010100110202002 2?15202114211 20?1-10?0001030?02-1210021210100000
00002-121?220000?100010031-?-0000213011311241000001000310020311
1241000400?0-?1012003212401 05000?-2----02101?02-1011-000?0050-2--0?
0??2--1??2-----00111000?12101101002??10000201101011?20111102000001
20030001323112011100122001210 0112?200?000

Hyrachyus_modestus
02?110101110000010-2-10122100--0-1010????1?121010001100010010012
0100112021021011 0011(13)32100001-101010(03)212102-0210121210101
01010002-0211220010410001011 00?20000211011111000001111100312?0
0121101000031000011111000210001040001?000110210110022111 10010
10400001020000?21212----0001010000112 0??01102?10001000010100 0?20
001000-0300?1001000?01100010 0000102(01)??0?1021211011100

Ephyrachyus
???????0?1?????12100000112101022001102201011212101212200111 21121
11110120210220122011102?22001-1?01102212112-1210101200101 00011
012-121102101??????????0??200?(01)1?00-111100?01111210(03)3(01)2(01)
0(01)121111000130010111210(01)01211101040?0510011102?0?11122211
1001?1040100002?000121212----100??
???

Fouchia
???????01?1111013101110112100--30110013000?1210000000--200?2001?0
0??0?2000011?00-???142002001-0110?03?10112-0210?0021010100 0100?2-
021?02100??????????01?200??211?1011100000122110?302??01?110000003
001011110100121100102000110011110101101211 0100?20003010011200
01121111----?-0??
??????????????001??11??????

Triplopus_youjingensis
??
??00?20?????
01-111100?00020??00311?101111110000300101110?200121110104000??0
0110101021012211110001004010001?0001021202----1-???????????????????
???

Triplopus_cubitalus
?????101?0000012102020000-00--01?111301110121?00101020?10112202
1110012021121000--11342112002?0001000112112-12112(02)1200??0000
20202-121?02201??????????0?0200102100-1?11000000111100201?1011110
100014001010110100121120105121?001010101011111221110000104000
00020000?21212----00010000000?20?011001??000?0?0????01??110???0000
20?????200??1????0???0000-?0????0001?????????

Epitriplopus_uintensis
???????01??????13300021123201302101000????2?221?00121500?2102210?1?
011?202??22?11111??4?22200210221?00(12)22202-021?101200123000313
-2-121?2221201100010120012014121 01-1111021034001103311110111111
21002?01101312101012112010?(01)1141000(12)02110211122211100011?
??100023000112121?----10020??0?1??????0???????0??0???003????000???00?1
30?????2000?2????0???000???0?????????????????

Triplopides_rieli
01010101------?13300021123201002?0?00??1?0?221?001?1200?21?1120?11
0?1?20??????1400??34?12200200221?00?22222-?212??1200120000313-2-?2
1?22012001???10?11---0?4??101-1111???034101103312100111231003210
101012101012123 11?11?04100??0??102?1122211?00011??0100013000101
1212----1002???0????????0??????0???0???0030??????????0????????0?0???2??????
?????????????????????????

Prohyracodon
???????????????1330202?112100--11011122111?1212001?0(12)--?211101211
1100120?102200(12)--11?41112002?0?21000212112-12112012101?000031
3-2-121?02012???
???????????0500??1001101010201122111 0000?1??010201301011 2122?----10
0??
???????????

Hyracodon_nebraskensis
?0?101001?11?111330002012320111210011201011221200121210?100111
?011001120210230121 0?114211100200001000002102-021220221 01?3000

20202-021?0220100101010111-?-0130212111111241024201200311110121124102200010101111001212411(01)11223100000101021 1?2211100000111010011200?101?212----00020?100112110?11001100001 0?0010300212010 1?00-130????020000200000102000102101 0?1121202001001

Ardynia

01000111-------1230002010???0--??0??12011??221?00??1?20?21?111?0110?1020?102??1?20?134?022002??22?00012??12-???12?1200?20000313-2-121?0221201100010121-?-00000-1111111001003111202312100121123100041111-21210001212411051?1?101-3?1010201?22211100021050102-1201?1?(12)?222----10010??0??????????0????00010?0????????0?????0??131?????00???1??0?0???000???0????10012?1001001

Gobioceras

??22?002?2-2-021222001???01000400021110002111222111002110500000013?001011222-----00???

Pappaceras

0???12001?1?02013311210113211001101 0?1111??1212101?1221?21?1101111??0120?102??12211134?12200210201003211112-?21(01)?1121012(12)100313-2-?21?2200103102010210012012 0???0???1100001211110031 2?00021101000121111111210000010101040004000011101021012221110001005000001130001021212----10000100011110011 00???11221101111 3000?001?1?01012001?11011101110?010?00?1030100101112111111 00

Fostercooperia

002?12001?1?0??1330122011 32?1001101111111001212101 21021?21?11011111002202102200 1--1?3?11(12)200210221003212112-121(01)?11210122100313-2-121?220?2001120102101120000112111111011003011100311110211101000011110211111001211101010 01(45)10(01)0(01)110102101(12)221110001001000001300010(12)1222-----0000??1??1110??1?1???1122100111?3000?001???????20????10??1?2??0?01??0011010??0?01110?12?1100

Proeggysodon_qiui

???1-000-1022(01)0?200?02101-111100?110112?013121001111000011420?02112100012112111 51?03?0011?1010211?221110000?1150?00013000?01?212----000???

Eggysodon
???????01?111111330?020123201012100001??1010221210121(12)(01)0121?
21??111?0112021022111(01)02133111?00100221100211112-0211?112101
20100?13-2-021?222022-???-?022001201312101-1111(01)100131011(01)13
1?1001211230011110100111111?01212?011410021000211010210?2211110
0011150100113000101121 2----000??
??

Juxia
0120100011?1?11330112011310120210100111110221210121400121?200
1111000120?10220110121331112002100201032111112-?211201210123000
?03-2-021?0200201212000010012013121101111100(01)0121121?0311110
02111100012111011111100211201050001100111110(01)0211122111100(
02)01050100003(01)0?1011212----100101010110200111 0?-?112200211113
000?001?1?010120????0111103111?010?00?1010010?0021010210110

Urtinotherium
???????01?01111133000020123?0101210000??1???221?101?12?0121?2023?1
1??1?20?1022?12002103100 2001-022010 0111?12-0211211210?23000?03-2
-021?021?201214?00100012024 12000-11110011340021003111121211130
01120010111111201211301120004100211101021012211110000012010 01
030001011212----100???
????????????????????0021011001000

Paraceratherium
1?4--4-01??????13300020123101302?00103311??221210??1201221??02311
1001020?102201221110311120021022010021100 2-02122?1210123000303
-2-021?020012---4--1-?1---013121?0?0?110011341011013121021211130001
2101011111120021130112000 3?0021110?0211?2211110000015 0?0010300
?1011212----10013101011120111001-?112200?11113??0?12-?0?011120??21
11201?3??111??000?1000010200312110011 00

Rostriamynodon_grangeri
?41?02?1------?12?0?0??10???0--??????????????????????????2??????????????????????
?????1?4?01?????0??1?0???????2-??1???21??1???0????2-??1??2?0?03102010231
---0???????????????010000110031 0?20?111020010????0?1?????0?211?111?0?0
3?0011?21?0?11?22?1110?021050100 12??0?1121?12----10110?????????01??0
??????1?0?0?1?3100?001?1??11120???001?0?1??000???00?11501001012101
1211100

Caenolophus_promissus

????????????????????????????????0??0??????121?001?00--?????????????????????????????1341?2?0?210?11100201002-??11111210111000120002-?21?0200???041014101-(01)11010?1(01)?221110000??

Amynodon

?0??03?1-------1(23)202220113200--3100101011?0221?001?00--?21?1003111001?20?002?000--1133112?0?21022110011?02-??11211210111?0012012-?21?1200?03?02010221---02010-01-1?010101001111002021011111121010412100102100012113110500031001(01)12110?110(12)2?1110000015010012200?1111212----1021111011?20010101-?1111?0?0?1?3100?001?????121???0010111010001?000?115001?101210?020?100

Sharamynodon_mongoliensis

0021?211-------1220202?113?00--310?10??1?1?121??01?00--?21??003?11001?20?002?000--213(02)112?0?210221?00?11002-??1??11210110?0012012-?21?1200?03102010231---01010-01-1?2-00013301110020210111111300 1?31010011110012113 01?00115?0000?2110?11?(12)2?1110002011010012?00?1?11222----1021010000?120011101-?112?10?0?1?3??0?001??11?12(01)????00101110?00???00?1150??0101?1201001110

Cadurcodon

?????3?1-------12203000103210--110??????????????????????21??000?110?1?20?0(01)2??0?--21341???0?21022110010?002-?211211211130001?002-?21?1200?3---2--0221---1---------------1341111001021011111130(01)1301212000111001112411110?1?101?01211021011 2?11100020111001 2200?11112?2----1021???????????????1-?????12?0??????0???????11?021?????01???2???12???00??160??3201?1201001110

Paramynodon

?????3?1------???2???000?11?11?20?002??00--2134111?0?210221100101?02-??11?1121011?00012012-?21?1200?031???10231---1---------------??????????????????????1041212000111001111311150 22?102001211021111221110001110 11?012?00?1111??2----102104??0?????????1?????0??0??11?1??0??00???1??120????012??100??????00??000??0??????????????

Metamynodon

01???3?1------?120-3-20123210--110310101110221200 1?00???21???00111?11020?0022000--2133??2?0?210221100101002-?211?11210113000 12012-?21?1210?(01)-102?1022????????????????????10001110010111011111300 1040?

120001110011124111150?2?1020012110211112?11100020050100 12?00?1111222----10221(23)01001010100101-?1?1112?011?3??1??01?0?010120????01201110?0110?000?1000?10?0?31011001000

Uintaceras_radinskyi

?????0001?1111?1(23)20222?113211001?0010111101121200121101221010031110111202102201101 0?141102001-0221000211112-0211?0121010000010202-021?0200?0??????0020012000?2101-11110011000011003121001111100?00?11110012100000110010410030000?0101011(01)02211110021004000011300?0?21212----10010?1??11???????????0?001?0?1???????01????0?020?????1?00?2??0?0????????30????0?102?11?1???

Teletaceras

0?3??1001?1111?13201120123201022100101111111212001211 20?20011011111?11202102201120213411120 02(01)0221003211112-021220120012300021202-021?0201?031?3?1012????????????????????0??201?02311?10??1111000??00?00111110002111010510 0??000?11000?10022111100210??000010210?0011212----100001000011200121 0???100011?01111110?010?1?0101200?2?0100001111?010000?0020??2??????????????

Trigonias

0?3011001?111111330202012320130110000301100(12)2120012150012102020211001(01)202102201(23)00213311120021022010021200 2-02122012(01)01230003-3-2-021?0221?031?3?11-1011201?13131102110210??0111003111100211021001220100111110001112111510141011010110012111100001150 10001?00?1111212----10012200111110 0?2101-1001012?01212110?02-???011120????01101021110 01?00010-100122000100000?100

Subhyracodon_occidentalis

1?30-1-01?11?1?13302020123201302100003011002212001?150?021?20201110?11202102??1221113311120?21022010021211 2-021220121112(03)0003-3-2-021?0201?1-1?3--1-1(01)0?2?24121311??11?3101200110231210 1?011231001210100111110 0?212311040?031010111010?110(12)211110001105010011?00?1111212----100020100012201121 0--1000012?01212110?22-???011120????02101020?10????00?0-20012201210110 11111

Menoceras_arikarense

1-3--4-01?01???13300021123?013021110?30110?221?001?152012 0?2120?110?1?202002??1520213421100?2102000001111 12-0212211211?200003-3-2-?21?0201?1-1-3--1-11---024?11211?2111310130111013111 10?2112410013111101111100121241111111410101110102101222110000111101001 1300?1121222----000123001011201121 01-1001012?01212110?22-?1?02012110?

?0210112021?0??000?0-21????1111001201110

Rhinoceros_unicornis

1-3--4101?0112113302021123201302111003011012212101215001201210
3?11?11?2020022?1520213421110021011010011211 2-0210001201121000
?1(23)-2-021?02111?---3--1--1---0000??2111111231000011100311(01)1000
1124100041211111?1?0010124110512211020001010210102211000 00105
0100013000110122 2----1002230000022011210 1-101101210?212111022-12
101110010200210112111011000010-2001020021001001

Supplementary References.

1. Wang JW. A new genus of ceratomorphs (Mammalia) from middle Eocene of China. *Vert PalAsiat* **26**, 20–34 (1988).
2. Radinsky LB. Early Tertiary Tapiroidea of Asia. *Bull Am Mus Nat Hist* **129**, 181–264 (1965).
3. Gabunia LK, Kukhaleishvili RE. A Zaysan *Rhodopagus* (Perissodactyla, Tapiroidea). *Paleontologicheskii Zhurnal* **25**, 107–114 (1991).
4. Lucas SG, Schoch RM. The systematics of *Rhodopagus*, a late Eocene hyracodontid (Perissodactyla: Rhinocerotoidea) from China. *Bulletin of the Geological Institutions of the University of Uppsala, N.S.* **9**, 43–50 (1981).
5. Dashzeveg D, Hooker JJ. New ceratomorph perissodactyls (Mammalia) from the Middle and Late Eocene of Mongolia: their implications for phylogeny and dating. *Zool J Linn Soc* **120**, 105–138 (1997).
6. Hooker JJ. Character polarities in early Eocene perissodactyls and their significance for *Hyracotherium* and infraordinal relationships. In: *The evolution of perissodactyls* (eds Prothero DR, Schoch RM). 79–101 Oxford University Press (1989).
7. Wood HE. Revision of the Hyrachyidae. *Bull Am Mus Nat Hist* **67**, 181–295 (1934).
8. Radinsky LB. *Hyrachyus, Chasmotherium*, and the early evolution of Helatetid tapiroids. *Am Mus Novit* **2313**, 1–23 (1967).
9. Radinsky LB. A review of the rhinocerotoid family Hyracodontidae (Perissodactyla). *Bull Am Mus Nat Hist* **136**, 1–46 (1967).
10. Schoch RM. Two unusual specimens of *Helaletes* in the Yale Peabody Museum collections, and some comments on the ancestry of the Tapiridae (Perissodactyla, Mammalia). *Postilla* **193**, 1–20 (1984).
11. Huang X, Wang J. Notes on *Hyrachyus* (Mammalia, Perissodactyla, Tapiroidea) from the Middle Eocene of Yunqu basin, Shanxi Province. *Vert PalAsiat* **40**, 211–218 (2002).
12. Gabunia LK. On a new member of Hyrachyidae (Perissodactyla, Ceratomorpha) from the Eocene of the Zaisan Depression (eastern Kazakhstan). *Paleontol J* **33**, 88–93 (1999).
13. Kordikova EG. A new Eocene vertebrate fauna from South-eastern Kazakhstan (Aktau Mountaains, Dzhungarian Alatau). *Vestnik KazGU* **5**, 180–185 (1998).
14. Huang XS, Qi T. Notes on late Eocene tapiroids from the Lunan Basin eastern Yunnan. *Vert PalAsiat* **20**, 315–326 (1982).
15. Thewissen JGM, Williams EM, Hussain ST. Eocene mammal faunas from northern Indo-Pakistan. *J Vert Paleont* **21**, 347–366 (2001).
16. Lucas SG, Holbrook LT, Emry RJ. The lophialetid ceratomorph *Eoletes*

(Mammalia) from the Eocene of the Zaysan Basin, Kazakstan. *Proceedings of the Biological Society of Washington* **114**, 988–992 (2001).
17. Ranga Rao A, Obergfell F. *Hyrachyus asiaticus*, new species of an upper Eocene tapiroid (Mammalia, Perissodactyla) from Kalakot, India. *Directorate of Geology, Oil & Natural Gas Commission, Dehra Dun, India, Special Paper* **3**, 1–8 (1973).
18. Chow MC, Qi T. Mammals from the Middle Eocene Kuanchuang Formation of Sintai, Shandong. *Vert PalAsiat* **20**, 302–314 (1982).
19. Qi T, Meng ZY. The discovery of *Hyrachyus modestus* and *Rhodopagus* sp. nov. Perissodactyla, Mammalia in Laiwu County, Shandong. *Vert PalAsiat* **21**, 103–108 (1983).
20. Qi T. The Middle Eocene Arshanto Fauna (Mammalia) of Inner Mongolia. *Ann Carnegie Mus* **56**, 1–73 (1987).
21. Wang HB, Bai B, Meng J, Wang YQ. A new species of *Forstercooperia* (Perissodactyla: Paraceratheriidae) from Northern China with a systematic revision of Forstercooperiines. *Am Mus Novit* **3897**, 1–41 (2018).
22. Wood HE. A primitive rhinoceros from the late Eocene of Mongolia. *Am Mus Novit* **2146**, 1–11 (1963).
23. Lucas SG, Schoch RM, Manning E. The systematics of *Forstercooperia*, a middle to late Eocene hyracodontid (Perissodactyla, Rhinocerotoidea) from Asia and Western North-America. *J Paleont* **55**, 826–841 (1981).
24. Wang HB, Bai B, Meng J, Wang YQ. Earliest known unequivocal rhinocerotoid sheds new light on the origin of Giant Rhinos and phylogeny of early rhinocerotoids. *Scientific Reports* **6**, 1–9 (2016).
25. Bai B, Meng J, Mao F-Y, Zhang Z-Q, Wang Y-Q. A new early Eocene deperetellid tapiroid illuminates the origin of Deperetellidae and the pattern of premolar molarization in Perissodactyla. *PLoS One* **14**, 1–26 (2019).
26. Wood H. Some early Tertiary rhinoceroses and hyracodonts. *Bull Am Paleontol* **13**, 5–105 (1927).
27. Hooker JJ. The beginning of the equoid radiation. *Zool J Linn Soc* **112**, 29–63 (1994).
28. de Bonis L, G., Bouvrain GK, Tassy. P. Un crâne de chalicothère (Mammalia, Perissodactyla) du Miocène supérieur de Macédoine (Grece): remarques sur la phylogénie des Chalicotheriinae. *Palaeovertebrata* **24**, 135–176 (1995).
29. Holbrook LT. Comparative osteology of early Tertiary tapiromorphs (Mammalia, Perissodactyla). *Zool J Linn Soc* **132**, 1–54 (2001).
30. Antoine P. Phylogénie et évolution des Elasmotheriina (Mammalia, Rhinocerotidae). *Mémoires du Muséum National d'Histoire Naturelle* **188**, 1–359 (2002).
31. Froehlich DJ. Quo vadis *Eohippus*? The systematics and taxonomy of the early

Eocene equids (Perissodactyla). *Zool J Linn Soc* **134**, 141–256 (2002).

32. Hooker JJ, Dashzeveg D. Evidence for direct mammalian faunal interchange between Europe and Asia near the Paleocene-Eocene boundary. In: *Causes and Consequences of Globally Warm Climates in the Early Paleogene* (eds Wing SL, Gingerich PD, Schmitz B, Thomas E). 479–500 Geological Society of America Special Paper (2003).
33. Hooker JJ, Dashzeveg D. The origin of chalicotheres (Perissodactyla, Mammalia). *Palaeontology* **47**, 1363–1386 (2004).
34. Colbert MW. The facial skeleton of the early Oligocene *Colodon* (Perissodactyla, Tapiroidea). *Palaeontol Electron* **8**, 1–27 (2005).
35. Anquetin J, Antoine PO, Tassy P. Middle Miocene Chalicotheriinae (Mammalia, Perissodactyla) from France, with a discussion on chalicotheriine phylogeny. *Zool J Linn Soc* **151**, 577–608 (2007).
36. Mihlbachler MC. Species taxonomy, phylogeny, and biogeography of the Brontotheriidae (Mammalia: Perissodactyla). *Bull Am Mus Nat Hist* **311**, 1–475 (2008).
37. Holbrook LT. Osteology of *Lophiodon* Cuvier, 1822 (Mammalia, Perissodactyla) and its phylogenetic implications. *J Vert Paleont* **29**, 212–230 (2009).
38. Bai B, Wang YQ, Meng J. New craniodental materials of *Litolophus gobiensis* (Perissodactyla, "Eomoropidae") from Inner Mongolia, China, and phylogenetic analyses of Eocene chalicotheres. *Am Mus Novit* **3688**, 1–27 (2010).
39. Hooker JJ. The mammal fauna of the early Eocene Blackheath Formation of Abbey Wood, London. *Monogr Palaeontogr Soc* **164**, 1–157 (2010).
40. Chen SK, Deng T, He W, Chen SQ. A new species of Chalicotheriinae (Perissodactyla, Mammalia) from the late Miocene in the Linxia Basin of Gansu, China. *Vert PalAsiat* **50**, 53–73 (2012).
41. Kondrashov PE, Lucas SG. Nearly complete skeleton of *Tetraclaenodon* (Mammalia, Phenacodontidae) from the Early Paleocene of New Mexico: morpho-functional analysis. *J Paleont* **86**, 25–43 (2012).
42. Danilo L*, et al.* A New Eocene Locality in Southern France Sheds Light on the Basal Radiation of Palaeotheriidae (Mammalia, Perissodactyla, Equoidea). *J Vert Paleont* **33**, 195 – 215 (2013).
43. Rose KD*, et al.* Early Eocene fossils suggest that the mammalian order Perissodactyla originated in India. *Nat Commun* **5**, 1–9 (2014).
44. Robinet C, Remy JA, Laurent Y, Danilo L, Lihoreau F. A new genus of Lophiodontidae (Perissodactyla, Mammalia) from the early Eocene of La Borie (Southern France) and the origin of the genus *Lophiodon* Cuvier, 1822. *Geobios* **48**, 25–38 (2015).
