# Supplementary material for: The origin of Rhinocerotoidea and phylogeny of Ceratomorpha (Mammalia, Perissodactyla)
Source: Commun Biol. 2020 Sep 14;3:509. doi: 10.1038/s42003-020-01205-8 (PMC7490376; doi:10.1038/s42003-020-01205-8)
Supplement: Supplementary file 2 — Reporting Summary [file 42003_2020_1205_MOESM2_ESM.pdf]

nature research

Corresponding author(s): Bin Bai

Last updated by author(s): Jul 26, 2020

Reporting Summary

Nature Research wishes to improve the reproducibility of the work that we publish. This form provides structure for consistency and transparency in reporting. For further information on Nature Research policies, see Authors & Referees and the Editorial Policy Checklist.

Statistics

For all statistical analyses, confirm that the following items are present in the figure legend, table legend, main text, or Methods section.

n/a	Confirmed	
☒	☐	The exact sample size (*n*) for each experimental group/condition, given as a discrete number and unit of measurement
☒	☐	A statement on whether measurements were taken from distinct samples or whether the same sample was measured repeatedly
☒	☐	The statistical test(s) used AND whether they are one- or two-sided<br>*Only common tests should be described solely by name; describe more complex techniques in the Methods section.*
☒	☐	A description of all covariates tested
☒	☐	A description of any assumptions or corrections, such as tests of normality and adjustment for multiple comparisons
☒	☐	A full description of the statistical parameters including central tendency (e.g. means) or other basic estimates (e.g. regression coefficient) AND variation (e.g. standard deviation) or associated estimates of uncertainty (e.g. confidence intervals)
☒	☐	For null hypothesis testing, the test statistic (e.g. *F*, *t*, *r*) with confidence intervals, effect sizes, degrees of freedom and *P* value noted<br>*Give P values as exact values whenever suitable.*
☒	☐	For Bayesian analysis, information on the choice of priors and Markov chain Monte Carlo settings
☒	☐	For hierarchical and complex designs, identification of the appropriate level for tests and full reporting of outcomes
☒	☐	Estimates of effect sizes (e.g. Cohen's *d*, Pearson's *r*), indicating how they were calculated

Our web collection on statistics for biologists contains articles on many of the points above.

Software and code

Policy information about availability of computer code

Data collection	Mesquite 3.6
Data analysis	Mesquite 3.6; TNT 1.5; MrBayes 3.2.8

For manuscripts utilizing custom algorithms or software that are central to the research but not yet described in published literature, software must be made available to editors/reviewers. We strongly encourage code deposition in a community repository (e.g. GitHub). See the Nature Research guidelines for submitting code & software for further information.

Data

Policy information about availability of data

All manuscripts must include a data availability statement. This statement should provide the following information, where applicable:

- Accession codes, unique identifiers, or web links for publicly available datasets
- A list of figures that have associated raw data
- A description of any restrictions on data availability

All specimens are deposited at the Institute of Vertebrate Paleontology and Paleoanthropology, Chinese Academy of Sciences, Beijing, China. Supporting data (character list and data matrix) for phylogenetic analyses for this study are provided in the Supplementary Information. Figure 2-7 are associated with raw data.

Field-specific reporting

Please select the one below that is the best fit for your research. If you are not sure, read the appropriate sections before making your selection.

☐ Life sciences ☐ Behavioural & social sciences ☒ Ecological, evolutionary & environmental sciences

Ecological, evolutionary & environmental sciences study design

All studies must disclose on these points even when the disclosure is negative.

Study description	This study reported some new material of ceratomorph fossils from the early Eocene of China, conducted the phylogenetic analysis of Ceratomorpha, and discussed the origin of rhinoceroses.
Research sample	This study was based on mammalian fossils, including some maxillae, mandibles, and isolated teeth.
Sampling strategy	There was no sampling strategy necessary.
Data collection	The correspond author collected and analyzed the data for the phylogenetic analysis by Mesquite and TNT.
Timing and spatial scale	The fossils were collected from the early and middle Eocene deposits in Inner Mongolia of China.
Data exclusions	No data were excluded.
Reproducibility	The data matrix will be uploaded to Morphobank (Project 3617) repository.
Randomization	The randomization is not applicable.
Blinding	The blinding is not applicable.

Did the study involve field work? ☒ Yes ☐ No

Field work, collection and transport

Field conditions	All fossils were collected from the late early Eocene deposits, which are mainly composed of sandstone and clay.
Location	All fossils were collected from the Erlian Basin of Inner Mongolia, China.
Access and import/export	All fossils were collected by surface prospecting or plaster jacket.
Disturbance	The disturbance is not applicable.

Reporting for specific materials, systems and methods

We require information from authors about some types of materials, experimental systems and methods used in many studies. Here, indicate whether each material, system or method listed is relevant to your study. If you are not sure if a list item applies to your research, read the appropriate section before selecting a response.

Materials & experimental systems

n/a	Involved in the study	
☒	☐	Antibodies
☒	☐	Eukaryotic cell lines
☐	☒	Palaeontology
☒	☐	Animals and other organisms
☒	☐	Human research participants
☒	☐	Clinical data

Methods

n/a	Involved in the study	
☒	☐	ChIP-seq
☒	☐	Flow cytometry
☒	☐	MRI-based neuroimaging

Palaeontology

Specimen provenance	The specimens were collected by crews of IVPP from Inner Mongolia of China.
Specimen deposition	Institute of Vertebrate Paleontology and Paleoanthropology, Chinese Academy of Sciences, Beijing, China
Dating methods	No new dates are provided.

☒ Tick this box to confirm that the raw and calibrated dates are available in the paper or in Supplementary Information.
